# An Integrated Module Performs Selective ‘Online’ Epoxidation in the Biosynthesis of the Antibiotic Mupirocin

**DOI:** 10.1002/anie.202410502

**Published:** 2024-10-16

**Authors:** Ashley J. Winter, Felix de Courcy-Ireland, Annabel P. Phillips, Joseph M. Barker, Nurfarhanim A. Bakar, Nahida Akter, Luoyi Wang, Zhongshu Song, John Crosby, Christopher Williams, Christine L. Willis, Matthew P. Crump

**Affiliations:** School of Chemistry, University of Bristol, BS81TS Bristol (UK); School of Chemistry, University of Bristol, BS81TS Bristol (UK); Department of Engineering and Sciences, School of Liberal Arts and Sciences, Taylor’s University, 47500 Subang Jaya, Selangor (Malaysia); School of Chemistry, University of Bristol, BS81TS Bristol (UK); Department of Chemistry, University of Barisal, 8200 Barisal (Bangladesh); Institute of Microbiology, Chinese Academy of Sciences, 100101 Beijing (China); School of Chemistry, University of Bristol, BS81TS Bristol (UK)

**Keywords:** Flavin monooxygenases, Mupirocin, Natural Products, Polyketides, Pseudomonic Acid

## Abstract

The delineation of the complex biosynthesis of the potent antibiotic mupirocin, which consists of a mixture of pseudomonic acids (PAs) isolated from *Pseudomonas fluorescens* NCIMB 10586, presents significant challenges, and the timing and mechanisms of several key transformations remain elusive. Particularly intriguing are the steps that process the linear backbone from the initial polyketide assembly phase to generate the first cyclic intermediate PA-B. These include epoxidation as well as incorporation of the tetrahydropyran (THP) ring and fatty acid side chain required for biological activity. Herein, we show that the mini-module MmpE performs a rare online (ACP-substrate) epoxidation and is integrated (‘*in-cis*’) into the polyketide synthase via a docking domain. A linear polyketide fragment with six asymmetric centres was synthesised using a convergent approach and used to demonstrate substrate flux via an atypical KS^0^ and a previously unannotated ACP (MmpE_ACP). MmpE_ACP-bound synthetic substrates were critical in demonstrating successful epoxidation in vitro by the purified MmpE oxidoreductase domain. Alongside feeding studies, these results confirm the timing as well as chain length dependence of this selective epoxidation. These mechanistic studies pinpoint the location and nature of the polyketide substrate prior to the key formation of the THP ring and esterification that generate PA-B.

## Introduction

**Figure 1:**
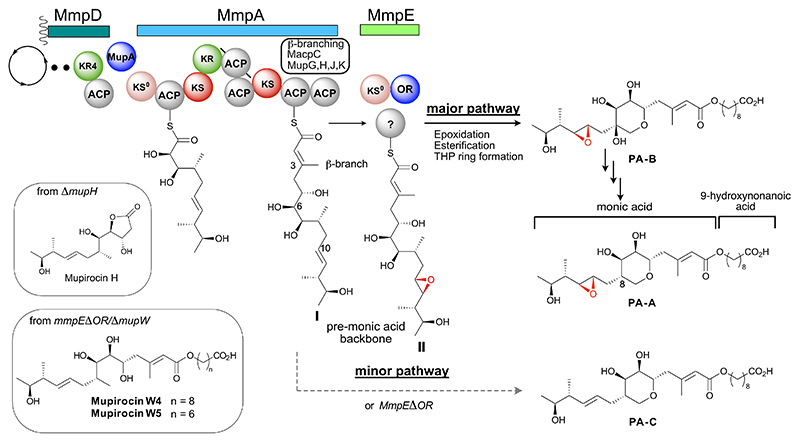
The MmpA/MmpE juncture of the mupirocin biosynthetic pathway. The terminal didomain ACPs of MmpA act as acceptor ACPs for the incorporation of a β-branch at C3. MmpE is arranged next in the biosynthetic sequence (this study) to perform a selective 10,11-epoxidation on the linear β-branched substrate en-route to PA-A. The minor metabolite PA-C lacks the epoxide.

Antibiotics that exhibit targeted efficacy are a powerful weapon in the fight against emergent bacterial resistance. Mupirocin, produced by *Pseudomonas fluorescens* NCIMB 10586, is a topical agent for Gram-positive bacteria with specific activity against methicillin-resistant *Staphylococcus aureus* (MRSA).^[1]^ Mupirocin exists as a mixture of pseudomonic acids (PAs) A-C, where the major component is PA-A (Figure 1). PAs contain a C_17_ monic acid polyketide backbone esterified to a 9-hydroxynonanoic acid (9-HN) moiety^[2]^ and bear several tailoring modifications including epoxidation,^[3]^ β-branching,^[4]^ α-hydroxylation^[5]^ and tetrahydropyran ring (THP) formation.^[6]^

Mupirocin is biosynthesised by a hybrid *trans*-acyltrans-ferase (*trans-*AT) polyketide synthase (PKS) and establishing the non-linear processing of the pathway has proven to be a long-standing challenge, requiring a combination of in vitro and in vivo approaches.^[2–3,5–7]^ The biosynthetic gene cluster contains six open reading frames (ORFs) encoding mupirocin multifunctional proteins *mmpA* to *mmpF* and 30 ORFs (including regulatory enzymes) [*mupA-mupZ* and five *trans*-acting acyl carrier proteins (ACPs), *macpA-macpE*].^[1,8]^ Assembly of the polyketide backbone of mupirocin occurs on the two large multi-modules which run in the order MmpD to MmpA (Figure 1). MupA catalyzed installation of the 6-hydroxyl group (6-OH) and formation of the β-branch (3-CH_3_) both occur within these modules, culminating with a branched intermediate **I** tethered to the functionally equivalent terminal ACPs of MmpA (each ACP is termed MmpA3a and MmpA3b).^[4]^ From the linear polyketide intermediate **I**, the precise sequence of biosynthetic steps leading to the first cyclic intermediate PA-B, is unknown but includes: epoxidation of the 10,11-alkene catalyzed by an oxidoreductase (OR) domain of MmpE (MmpE_OR);^[3]^ formation of the THP ring catalyzed by the joint action of *trans*-acting MupW/MupT and MupZ;^[6]^ and generation of the 9-HN ester (the latter step may involve esterification followed by fatty acid elongation or vice-versa and current evidence can support either mechanism).^[2b]^ In the final series of biosynthetic steps, PA-B is converted to the major product PA-A via the removal of a 8-OH group.^[7c]^ Delineating how PA-B is formed is therefore one of the key remaining questions.

In previous studies, analysis of cultures of the mutant strain *mmpEΔOR* of *P. fluorescens* revealed no metabolites possessing a 10,11-epoxide and the major product was PA-C with the 10,11-alkene (Figure 1).^[3,9]^ Additionally, cultures from the double mutant strain, *mmpEΔOR/ΔmupW* gave two new homologs, mupirocin W4 and W5, again both with the 10,11-alkene.^[3]^ Taken together these results confirm the role, but not the timing, of the MmpE_OR in 10,11-epoxidation (Figure 1).

Interestingly, both mupirocin W4 and W5 lack the THP ring and possess appended fatty acids with differing carbon chain lengths. Hence, the divergence of the biosynthetic pathway into two parallel pathways, one yielding PA-B/PA-A with the 10,11-epoxide and the other producing the minor metabolite PA-C with the 10,11-alkene, suggests epoxidation may occur closely after polyketide backbone assembly.^[3]^

It is firmly established that following assembly of the polyketide backbone by MmpD and MmpA, the polyketide intermediate is tethered to MmpA3a/b.^[4]^ However, the fate of the polyketide chain from this point is unknown and if via epoxidized intermediate **II**, where does this reside? (Figure 1). Here we use a multi-factored approach to confirm how the epoxidizing mini module, MmpE, is structurally and mechanistically incorporated into the terminal phase of mupirocin polyketide backbone assembly, thereby significantly reducing the number of permutations of how the final biosynthetic steps to PA-B are arranged.

## Results and Discussion

MmpA terminates with the MmpA3a/b ACP didomain but lacks an *in-cis* thioesterase (TE) domain. A terminal TE domain would off-load the polyketide, permitting further processing of an ACP-free intermediate as observed in numerous other PKSs. This suggests that downstream processing requires either translocation via a downstream ketosynthase (KS) domain or chain release via a *trans*-acting hydrolase. A further multidomain module, MmpE, contains a C-terminal OR domain known to control epoxidation of the 10,11-alkene, as well as an N-terminal non-elongating ketosynthase (MmpE_KS^0^). *mmpE* is not co-located with either *mmpA* or *mmpD* but does immediately follow the five genes encoding the HMGS cassette, suggesting it may act directly after the β-branching step. Further, PA-C lacks the 10,11-epoxide and is formed by processing of the polyketide chain in a parallel pathway to PA-A (Figure 1).^[3]^ This point of divergence of these two biosynthetic routes is also consistent with epoxidation occurring just after β-branching and prior to THP ring formation and fatty acid esterification. Conversely, the MmpE_KS^0^ has a methionine preceding the cysteine in the active site that is typical of condensing KS domains that reject branched or bulky fatty acids, suggesting this may not be the correct sequence of events.^[10]^ Therefore, we were intrigued as to whether MmpA and MmpE worked in a sequential fashion, with MmpE performing a rare online epoxidation to yield **II** (Figure 1).

**Figure 2:**
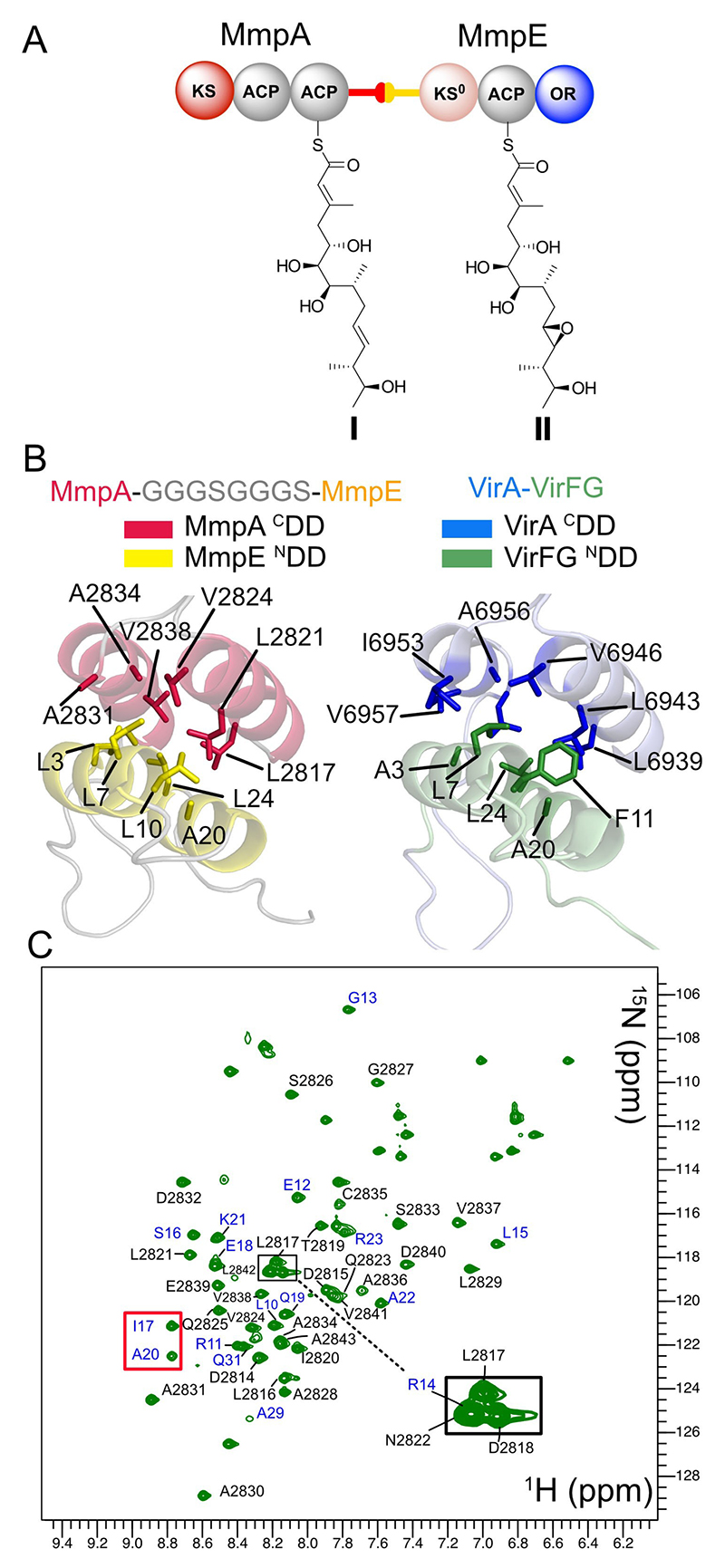
The MmpA/MmpE docking domain. A) Proposed molecular architecture of the C-terminal of MmpA docked with MmpE and predicted processed polyketide intermediates. B) Structural predictions of the MmpA ^C^DD and MmpE ^N^DD and comparison to the virgin-iamycin 4HB. C) ^1^H−^15^N HSQC NMR spectrum of MmpE_ACP.

Initially, the functional annotation of the full-length MmpE module was re-examined. As well as possessing the N-terminal MmpE_KS^0^ and the C-terminal MmpE_OR, the module contains an intervening 148 amino acid sequence as well as an N-terminal extension of about 44 amino acids. Further investigation of this linker region revealed, surprisingly, a previously unannotated ACP sequence (MmpE_ACP) containing a characteristic conserved serine residue within a phosphopantetheine (Ppant) transferase recognition motif (Figure S1). The presence of this motif suggested this ACP might be active as opposed to an evolutionary structural remanent (i.e. not an ACP^0^). Therefore, if confirmed, MmpE might act downstream of MmpA and provide a suitable off-loading mechanism via transfer to MmpE_KS^0^, followed by the MmpE_ACP. With MmpE_ACP bearing the substrate, the adjacent *in-cis* OR would be correctly positioned to rapidly epoxidize the substrate. Once transformed, the MmpE_ACP would become a hitherto previously unknown molecular recognition platform that might direct the next key tailoring step (Figure 2A).

The unannotated C-terminal amino acid sequence of MmpA (31 aa) and the N-terminal of MmpE (44 aa) were therefore examined for potential structural docking domains (Figure S2). This revealed that both terminal regions contained candidate docking domain motifs (referred to as ^C^DD and ^N^DD henceforth), previously shown to be responsible for the transfer between specific PKS modules in both *cis* and *trans*-AT systems,^[11]^ with the latter *trans*-AT ^C^DD/^N^DD forming either four α-helix bundles (4HB) or a dehydratase docking domain.^[12]^

A fusion construct of MmpA-MmpE (MmpAE) was generated where the ^C^DD and ^N^DD were concatenated via a GGGSGGGS linker, with ab initio homology modelling^[13]^ predicting a 4HB arrangement similar to the previously characterised VirA-VirFG intermodular docking domain (Figure 2B, Figure S3) involving a similar ACP-KS juncture.^[12a]^ Recombinantly expressed MmpAE was monomeric and displayed an α helical profile by CD (Figure S3) and was judged to be folded by the well-dispersed resonances in a ^1^H−^15^N HSQC NMR spectrum (Figure 2C) of a ^15^N-enriched sample of the protein. The isolated MmpE ^N^DD domain was prepared as a control and showed a poor one-dimensional NMR profile (eg broad signals, lack of dispersion) compared to MmpAE and lacked several well dispersed amide signals that were observable and assigned in the folded construct (eg I17 and A20 Figure 2C, Figure S3). Both observations were consistent with a lack of independent folding of MmpE ^N^DD. Attempts to prepare MmpA ^C^DD did not yield soluble protein. Overall, this strongly suggested formation of a 4HB-type docking interface and integration of MmpE into the modular PKS assembly.

**Figure 3:**
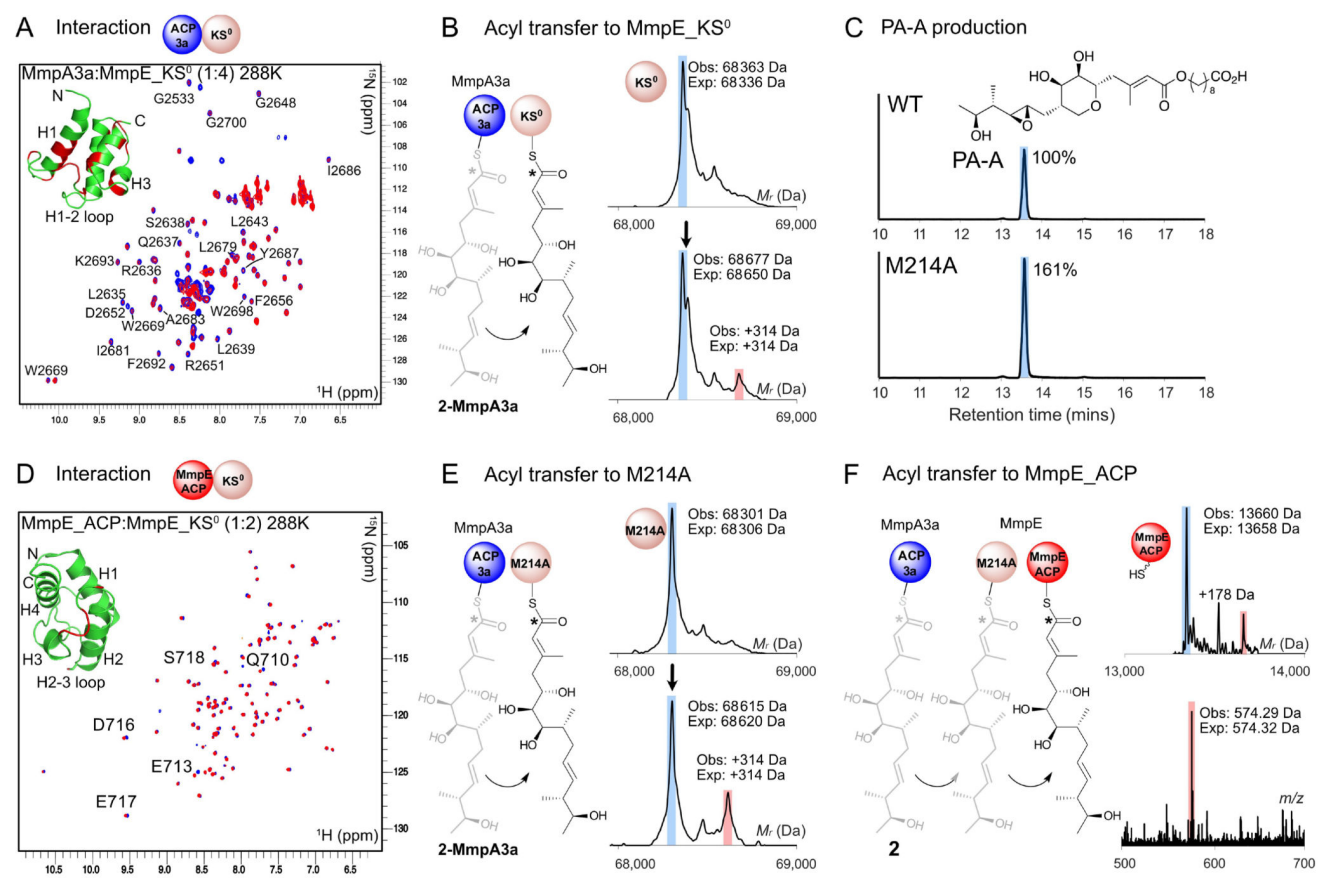
Interaction and acyl transfer between MmpA3a and MmpE. A) ^1^H−^15^N HSQC NMR spectra of ^15^N labelled MmpA3a (‘ACP3a’) before (blue correlations) and after (red) addition of MmpE_KS^0^. Inset – perturbations are shaded in red on MmpA3a (PDB 2L22). B) ESMS of MmpE_KS^0^ showing transfer of **2** (Obs: + 314 Da (from observed mass of MmpE_KS^0^), Exp: + 314 Da). C) Comparison of PA-A titres from WT and MmpE_KS^0^ M214A in *P. fluorescens*. D) ^1^H−^15^N HSQC NMR spectra of ^15^N-MmpE_ACP before (blue correlations) and after (red) addition of MmpE_KS^0^. E) As B) using MmpE_KS^0^_M214A F) ESMS of MmpE_ACP showing transfer of **2** from MmpA3a via MmpE_KS^0^_M214A to holo-MmpE_ACP.* = ^13^C label.

To further explore the function of this interface, MmpE_KS^0^ was cloned, expressed and purified (Figure S4). Purified MmpE_KS^0^ was then titrated with ^15^N-labelled *apo*-MmpA3a^[4]^ and the ACP was monitored by NMR using ^1^H−^15^N HSQC spectra (following backbone chemical shift assignment; Figure S4). Comparison of these two-dimensional spectra of the free ACP versus in the presence of a two-fold excess of KS^0^ revealed clear loss of peak intensities for residues in the core of the ACP structure and the flexible loop connecting helices 1 and 2 (Figure 3A). Unassigned residues from the flexible N-terminal tag (non-native sequence) were visible in the central region of the ^1^H−^15^N HSQC and were largely unperturbed, consistent with interactions with the core ACP structure. Overall, this was indicative of a potential interaction between the two proteins. Note that both constructs lacked their ^C^DD and ^N^DD components but nonetheless were able to form an observable interaction further highlighting that the docking domain plays a role in bringing modules in proximity but is not essential for formation of protein-protein interactions of the constituent domains as observed in class I *cis*-AT PKSs.^[14]^

**Scheme 1:**
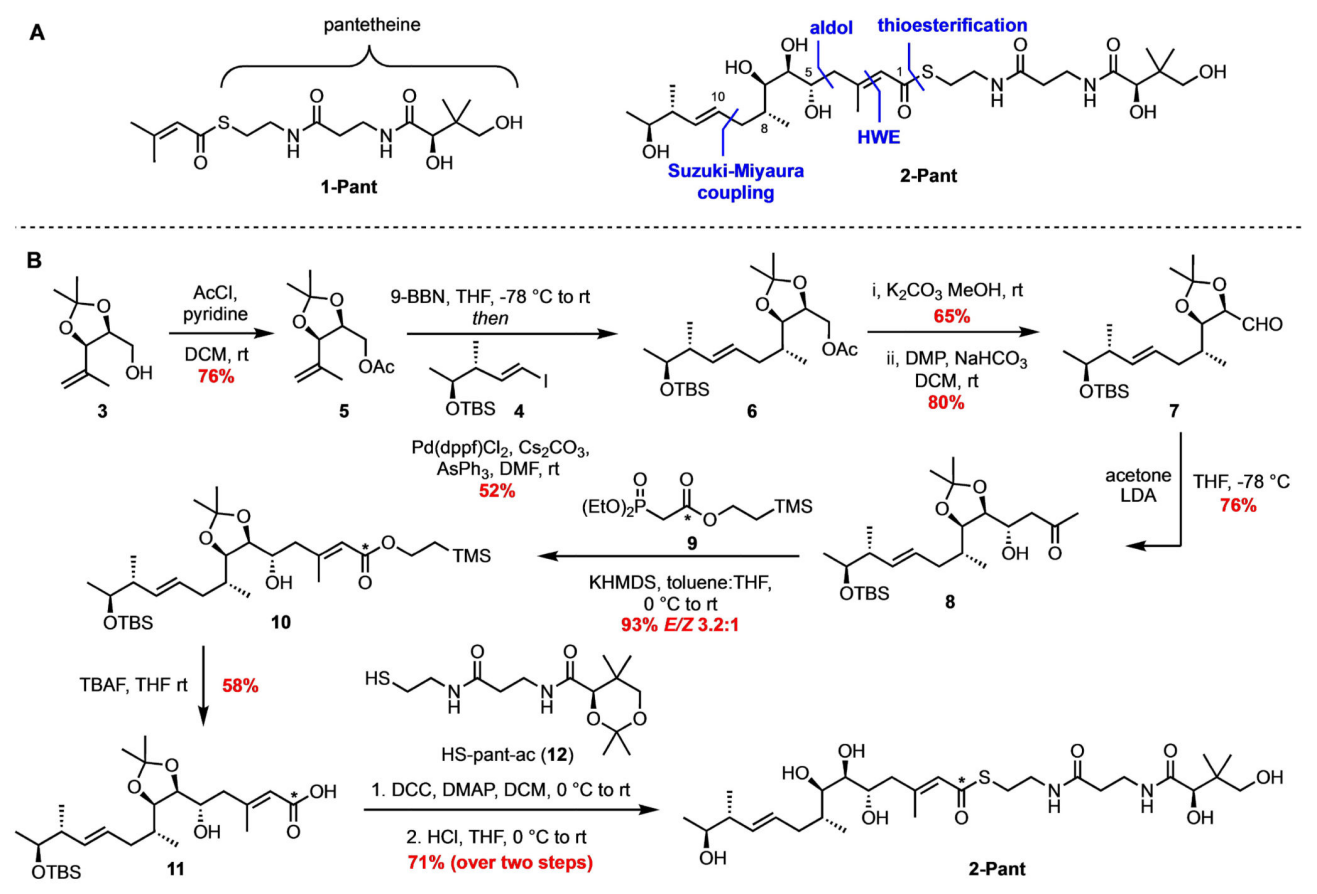
Synthesis of α,β-unsaturated thioester **2-Pant**. * = ^13^C label. DCC=dicyclohexylcarbodiimide, DMAP = 4-dimethylaminopyridine, DMP=Dess–Martin periodinane, dppf = 1,1’-bis(diphenylphosphino)ferrocene, KHMDS = potassium (bistrimethylsilyl)amide, LDA = lithium diisopropylamide, TBAF = tetrabutylammonium fluoride, TBS=tert-butyldimethylsilyl, TMS=trimethylsilyl, 9-BBN = 9-borabicyclo(3.3.1)nonane.

We therefore turned to chemoenzymatic methods to test if thioester substrates could be transferred between MmpA3a and the MmpE_KS^0^. Initially we selected 3-methyl-2-butenoyl (MBE) pantetheine derivative **1** which we reported recently, and which incorporates a β-methyl branch into a truncated thioester mimic (Scheme 1A).^[15]^ *Apo-*MmpA3a was chemoenzymatically loaded with pantetheine-**1** to yield **1**-MmpA3a (observed 14461 Da; expected 14460 Da; Figure S5). When **1**-MmpA3a was combined with KS^0^ (*M*_r_ 68336 Da) and the mixture monitored by ESMS, it was possible to directly detect a mass increase of 80 Da (exp + 82 Da) on the target KS^0^. Concomitant generation of *holo*-MmpA3a was also detected as MBE-MmpA3a was consumed (data not shown). Translocation was not observed when MBE was substituted for a propionyl mimic (Figure S5).

This sequence would suggest polyketide transfer from MmpA3a/b to MmpE_KS^0^ occurs immediately after β-branching. To confirm the specificity of the KS^0^ and examine in greater detail the function of the MmpE ACP and OR a sample of the pantetheine analogue **2-Pant** of intermediate **I** was required (Figure 1 and Scheme 1). In designing a synthetic strategy to thioester **2-Pant**, a modular approach was proposed which could be adapted for the introduction of a carbon-13 label at C-1 with a view to further downstream esterification assays (Scheme 1A). Four of the six stereocentres in the polyketide portion of **2-Pant** were provided from known starting materials, alcohol **3** prepared from D-ribose^[16]^ and vinyl iodide **4** from commercially available methyl (*S*)-3-hydroxybutanoate.^[5]^ Following acetylation of alcohol **3** to ester **5**, an in situ hydroboration with 9-BBN and Suzuki–Miyaura cross-coupling with vinyl iodide **4**^[16]^ gave novel acetate **6** with creation of a new *R*-stereocentre and *E*-alkene (Scheme 1B). Ester hydrolysis and oxidation of the resultant primary alcohol with DMP^[17]^ furnished aldehyde **7** which then was used in a stereo-selective aldol reaction^[18]^ with the lithium enolate of acetone giving β-hydroxyketone **8** in 76 % yield and > 20 : 1 d.r. in favour of the required diastereomer. Phosphonate **9** was prepared in two steps from bromoacetic acid in 72 % overall yield (Supporting Information, Scheme S1) and used in a Horner–Wadsworth–Emmons (HWE) reaction with ketone **8** to generate (*E*)-trisubstituted alkene **10**. Selective deprotection of the trimethylsilylethyl ester with TBAF gave carboxylic acid **11** as the major product. With the polyketide fragment complete, **11** was readily coupled with protected pantetheine **12**^[19]^ and global deprotection with HCl in THF gave the target substrate **2-Pant** in 71 % yield over the final two steps.

Chemoenzymatic loading of MmpA3a with substrate **2-Pant** successfully yielded the modified ACP with > 90 % conversion (observed: 14693 Da; expected: 14691 Da). ESMS Ppant ejection assays were used to fragment the Ppant arm from the ACP whilst leaving the substrate intact, thus enabling accurate detection of low molecular weight intermediates.^[20]^ Ppant ejection yielded the expected ion (observed: 574.33 Da; expected: 574.32 Da; Figure S6). Incubation of MmpE_KS^0^ with derivatised **2**-MmpA3a and analysis via ESMS revealed the presence of a second species corresponding to direct transfer of the entire polyketide substrate (Figure 3B; obs: KS^0^ mass of 68363 Da increases to 68677 Da (obs + 314 Da, expected + 314 Da)). Concurrent hydrolysis of **2**-MmpA3a to form *holo-*MmpA3a was also observed (data not shown). Inspection of the active site cysteine of MmpE_KS^0^ and the preceding amino acid residue (X–Cys) revealed a methionine at position 214. This was somewhat unexpected given bioinformatic analysis of 150 *trans*-AT KS domains and experimental data has previously suggested that this position is occupied by an alanine or glycine in KS domains that accept a β-branched or bulky fatty acid.^[10,21]^ We therefore generated both an *mmpE-KS^0^-M214A* and -*C215A* point mutation in *P. fluorescens* NCIMB 10586 to confirm the role of this KS^0^ in vivo and to test if M214A influenced PA-A production. Analysis of titres from *P. fluorescens mmpE-KS^0^-C215A* showed complete loss of PA-A production and generation instead of mupirocin H (Figure 1 and Figure S7).^[22]^ This provided further evidence for the absolute requirement of the MmpE_KS^0^ in mupirocin biosynthesis. This was also consistent with our hypothesis that the action of MmpE directly follows MmpA as mupirocin H production predominantly occurs when mutations are introduced into enzymatic domains involved in the initial polyketide assembly phase. For the M214A mutant and measurement of comparative yields, *P. fluorescens mmpE-KS^0^-M214A*, was cultured in parallel with the original wild-type (WT) strain under the same fermentation conditions. PA-A was isolated and purified as previously described^[3]^ and when compared to WT, PA-A production in *mmpE-KS^0^-M214A* was observed to increase by about 1.5-fold (Figure 3C, Figure S7) as measured by evaporative light scattering and UV detection.

With the confirmation that MmpA3a was able to transfer the native polyketide substrate to MmpE_KS^0^, we cloned, expressed, and purified the *in-cis* MmpE_ACP domain that lies immediately adjacent to the KS^0^ (Figure S8). Analysis by CD and ^1^H−^15^N HSQC NMR spectroscopy determined this excised domain was folded and backbone amide chemical shift assignments and NOEs confirmed α-helicity over key regions of the protein, i.e. helices 2 and 3 that are the principal protein-protein interaction regions of ACPs (Figure S8). NMR confirmed weak interactions with MmpE_KS^0^ centred on the loop region between helix 2 and 3 although these were less pronounced than those observed with MmpA3a (Figure 3D). Nonetheless along with the generally accepted collinearity of substrate passing between adjacent KS-ACP pairs, these interactions support the canonical transfer of substrate to MmpE_ACP. To support our in vivo observations of increased PA-A production in *mmpE-KS^0^-M214A* fermentations, we cloned, expressed and purified MmpE_KS^0^_M214A as a soluble construct (Figure S4). Incubation of MmpE KS^0^ M214A with **2**-MmpA3a and analysis via ESMS indicated an increased proportion of the polyketide substrate was transferred to MmpE KS^0^ M214A when compared to MmpE_KS^0^ (obs: 68615 Da vs 68301 Da (Obs: + 314 Da, Exp: + 314 Da); Figure 3E).

Finally, we attempted a tri-component assay to observe chain transfer from MmpA3a to MmpE_ACP via MmpE_KS^0^. Initially when this was attempted by incubating **2**-MmpA3a, MmpE_KS^0^ and *holo*-MmpE_ACP together, transfer to MmpE_KS^0^ was again observed but in insufficient quantity to observe subsequent transfer to *holo*-MmpE_ACP. However, incubation of **2**-MmpA3a, *holo*-MmpE_ACP and M214A (to take advantage of the increased transfer efficiency of the KS^0^ mutant) resulted in direct transfer of the polyketide substrate from MmpA3a to MmpE_ACP via MmpE_KS^0^_M214A (obs: 13,660 Da, exp: 13,658 Da). Ppant ejection yielded the expected ion (obs: 574.29 Da, exp: 574.32 Da; Figure 3F), providing definitive evidence of transfer from modules MmpA to MmpE.

Having shown transfer of the polyketide chain to MmpE_ACP, we turned to the epoxidation step and MmpE_OR. MmpE_OR is predicted to be a flavin-dependent monooxygenase (FMO) which are grouped into eight subclasses (A-H) based on their amino acid sequence, structural fold and activity.^[23]^ Sequence analysis and ab initio modelling^[13]^ of MmpE_OR revealed it resembled other class A FMOs, with high sequence similarity to the archetypal class A FMO *P. fluorescens p-*hydroxy-benzoate hydroxylase (PHBH; PDB: 1PBE; see Figure S9 for further details).^[24]^ Class A FMOs lack a NAD(P)H reductase domain and utilise FAD and NAD(P)H directly to catalyse hydroxylation, sulfoxidation, epoxidation and Baeyer–Villiger reactions.^[25]^

**Figure 4:**
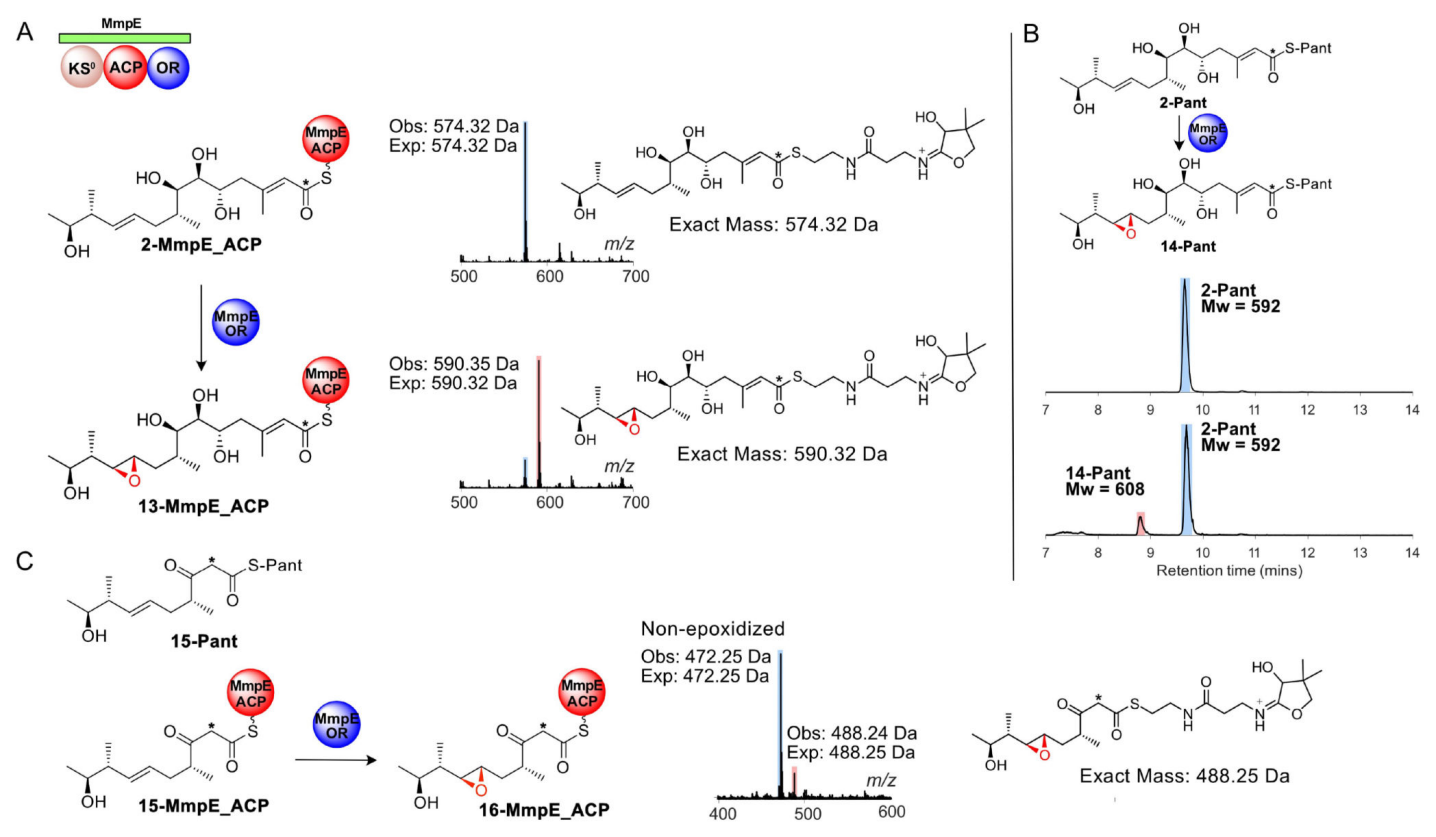
**2**-MmpE_ACP epoxidation. A) ESMS assay demonstrating 10,11-epoxidation of **2**-MmpE_ACP. B) Partial conversion of **2**-Pant in the absence of MmpE_ACP. C) Partial conversion (18 %) of **15**-MmpE_ACP. * indicates a ^13^C label.

To demonstrate specific epoxidation of **2**-MmpE_ACP, the gene encoding MmpE_OR was cloned, over-expressed and purified to homogeneity (Figure S4). MmpE_OR was soluble and monomeric by size exclusion chromatography (SEC) and when purified, the protein solution had a yellow colour, confirming the presence of a flavin cofactor. **2-Pant** was chemoenzymatically loaded onto *apo*-MmpE_ACP to yield **2**-MmpE_ACP (observed: 13659 Da; expected: 13658 Da), with Ppant ejection yielding the expected ion (observed: 574.32 Da, expected: 574.32 Da; Figure 4A and Figure S6). **2**-MmpE_ACP was then incubated with MmpE_OR alongside excess FAD and NADH. After 15 minutes, ESMS analysis revealed consumption of **2**-MmpE_ACP and 80 % conversion to a new MmpE_ACP species (Figure 4A and Figure S10). This species showed an addition of 16 Da, consistent with epoxidation to give **13**-MmpE_ACP (observed: 13,675 Da, expected 13,674 Da). Ppant ejection of this species revealed a fragment at 590.35 Da, a species consistent with an addition of 16.03 Da (*cf* 574.32 Da). This, alongside non-productive assays that substituted **2**-MmpE_ACP for *holo*-MmpE_ACP, confirmed that epoxidation occurred on the conjugated substrate and not elsewhere on the ACP (Figure S6 and Figure S11). A further control experiment using denatured MmpE_OR gave no turnover, confirming the catalytic role of the enzyme (Figure S11). Finally, addition of *E. coli* NAD(P)H:flavin oxidoreductase: Fre, previously used to catalyse α-hydroxylation of sub-strates by MupA via the generation of reduced flavin, resulted in a slower conversion of **2**-MmpE_ACP to **13**-MmpE_ACP confirming MmpE_OR only requires FAD and NADH for epoxidation and its classification as a class A FMO (Figure S11). Incubation of the free pantetheine substrate **2** with MmpE_OR, FAD and NADH in the absence of MmpE_ACP was monitored by HPLC (Figure 4B). After incubation, approximately 15 % of **2-pant** (m/z 592.59 [M+H]^+^) was converted to a new species with + 16 Da (m/z 608.55 [M+H]^+^) corresponding to **14-pant**, suggesting less efficient epoxidation when uncoupled from MmpE_ACP (Figure 4B).

To detect whether MmpE_OR displayed a rigid substrate specificity towards **2**, we selected a shorter pantetheine derivative **15-Pant** (Figure 4C). **15-Pant** bears the equivalent 10,11-alkene and was previously used in our 6-hydroxylation assays using MupA.^[5]^ **15-Pant** was converted to the CoA derivative and successfully loaded onto MmpE_ACP (observed: 13556 Da, expected: 13556 Da) with a characteristic Ppant ejection ion (observed: 472.27 Da, expected: 472.25 Da; Figure S6). Addition of MmpE_OR, FAD and NADH to **15**-MmpE_ACP resulted in significant hydrolysis to *holo*-MmpE_ACP, alongside a small proportion of epoxidation (ca. 18 %) to **16**-MmpE_ACP (observed: 13571 Da, expected 13572 Da). Ppant ejection of this species confirmed the addition of 16 Da (observed: 488.24 Da compared to 472.25 Da; Figure 4C and Figure S10).

**Figure 5:**
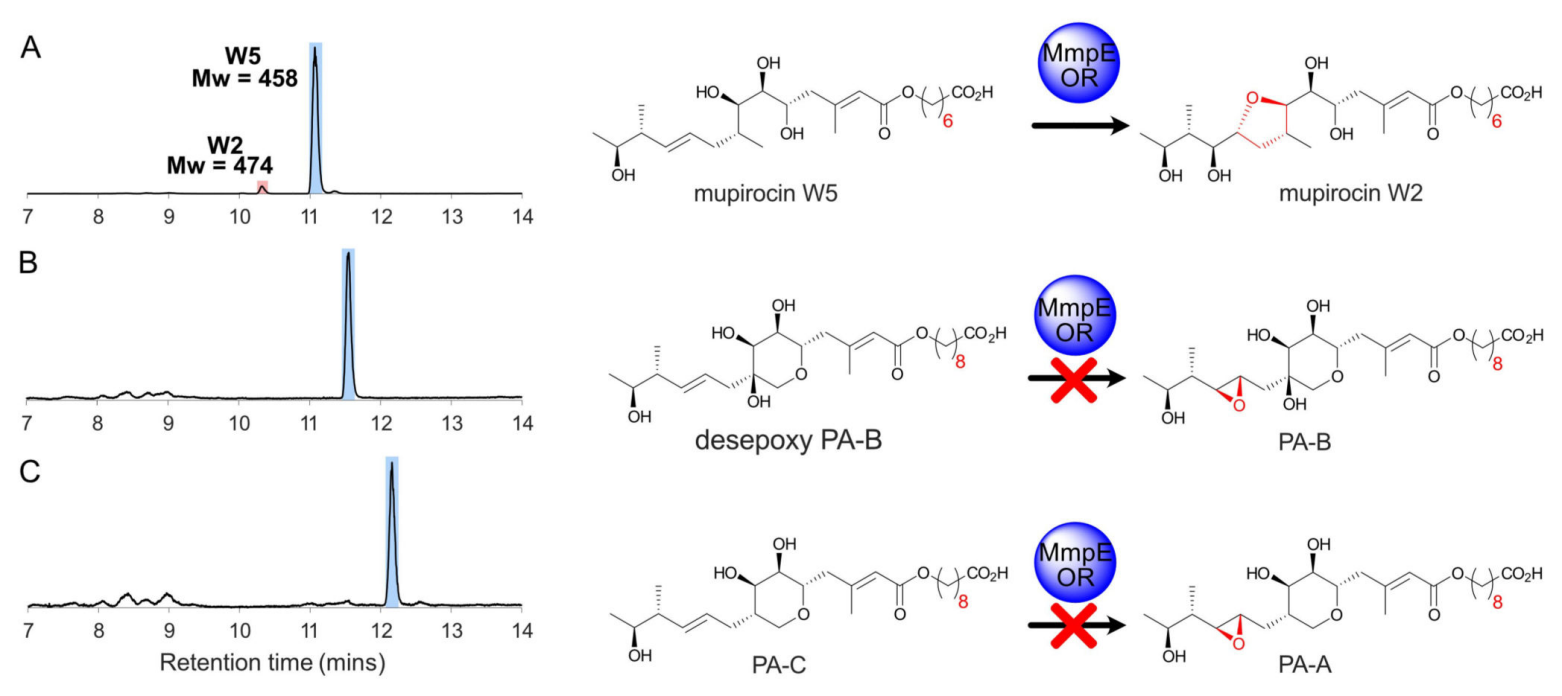
Feeding studies with *E. coli* overexpressing MmpE_OR. HPLC traces of extracted metabolites post-feeding for A) mupirocin W5 (blue) and mupirocin W2 (red) which were also characterised by NMR. B) Desepoxy PA-B. C) PA-C.

To further explore the substrate specificity and timing of action of MmpE_OR, we utilised in vivo biotransformations using *E. coli* overexpressing MmpE_OR. A series of substrates including mupirocin W5 as well as desepoxy PA-B and PA-C were selected to test whether more elaborate structures were accepted, and specifically whether the presence of the THP ring or fatty acid side chain would influence the reaction. After incubation, approximately 5 % of mupirocin W5 (*m/z* 457.28 [M −H]^−^) was converted into a species with + 16 Da (Figure 5A). Isolation and NMR characterisation of the resulting product confirmed this to be mupirocin W2 (*m/z* 473.29 [M −H]^−^), previously isolated as a minor component from *P. fluorescens* WT and knockout strains (eg Δ*mupW*; Figure S12).^[3]^ Mupirocin W2 arises from opening of the 10,11-epoxide following attack from the 7-OH group which is facilitated by the conformational freedom arising from the lack of the THP ring. Conversely both desepoxy-PA-B and PA-C failed to show detectable turnover (Figure 5B, C).

**Figure 6:**
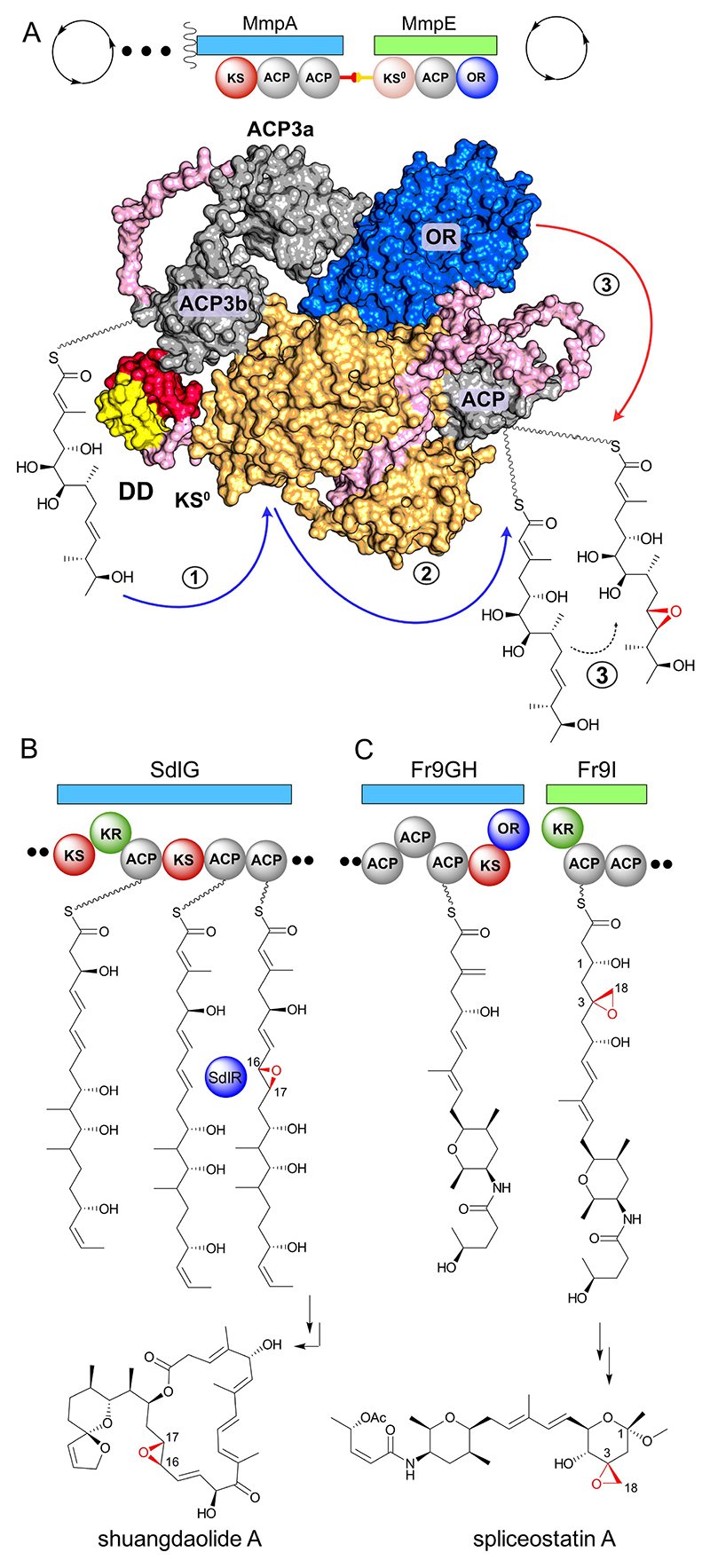
A) Cartoon representation of AlphaFold model for MmpA and MmpE.^[13]^ The model includes the terminal tandem ACPs, ACP3a and 3b (gray), the ^C^DD and ^N^DD docking domains (red and yellow respectively), MmpE_KS^0^ (orange), MmpE_ACP (gray), MmpE_OR (blue) and linker regions (pink). B) The epoxidation step in shuangdaolide biosynthesis. The FMO is integrated into SdlR which is comprised of four domains, including a KR, ACP, FMO and FSH1 (Serine Hydrolase), but only the FMO domain is active. C) The epoxidation step in spliceostatin biosynthesis.

The above results firmly establish that the mupirocin multi-module assembly is arranged as MmpD-MmpA-MmpE. MmpA and MmpE are linked by a 4HB docking domain confirming MmpE to be an integral module acting *in-cis* and not *in-trans* as previously speculated.^[26]^ The β-branched intermediate **I** (Figure 1) passes from MmpA to the non-elongating KS^0^ of MmpE. For further polyketide processing to occur, collinearity would predict transfer to an adjacent ACP, which we have now identified as the MmpE_ACP that independently folds and is functional. We have demonstrated transfer of substrate to MmpE_ACP which can undergo efficient MmpE_OR catalyzed epoxidation (Figure 6A). Whilst these observations now define specific steps in the mupirocin biosynthetic pathway, they also highlight several important features of broader significance.

KS^0^ domains are proposed to be important for substrate selectivity and exchanging a substrate between two ACPs, thereby refreshing the protein-protein interactions and biosynthetic transformations at the disposal of the second ACP-substrate conjugate. Initially, the KS^0^ was not expected to accept a β-branched substrate based on amino acid residue sequence analysis.^[12b]^ Previous experimental observations around β-branch selectivity were based on elegant ESMS experiments using a range of acyl-SNACs that explored the selectivity of both elongating and non-elongating KSs. The non-elongating KS^0^ from module D of the psymberin (Psy) biosynthetic pathway, PsyD KS3^0^, possesses a methionine at position 237 and did not accept a β-branched MBE-SNAC substrate.^[27]^ More elaborate ACP-bound intermediates were not available which was presented as a limitation of the study at the time. When we repeated these experiments using the loaded ACPs **2**-MmpA3a, MBE-MmpA3a, propionyl-MmpA3a and MmpE KS^0^, we found that **2** and MBE were transferred and, surprisingly, the propionyl group was not. It therefore appears that in the context of more authentic protein-protein interactions and substrates, the selectivity, certainly of KS^0^s, can be finetuned. Sequence analysis of 64 *trans*-AT KS domains immediately downstream of acceptor ACP domains within β-branching modules of 44 biosynthetic pathways revealed that only MmpE_KS and TmpE_KS contained a Met-Cys motif (Figure S13). 94 % contained Ala/Gly-Cys motifs, indicating the previously reported trend largely holds. In contrast, CalH_KS1 (Val-Cys) and BonD_KS3 (His-Ser) are exceptions to this rule, although BonD_KS3 may be involved in substrate offloading as opposed to functioning as a strict KS^0^.^[28]^ We also determined that, surprisingly, an in vivo M214A mutation in the KS^0^ increased PA-A production. Although this indirect in vivo assay did not directly probe the underlying mechanism of increased PA-A titre, one possibility is the action of the Met-Cys motif may modulate transfer efficiency between MmpA and MmpE. Subsequent in vitro experiments with **2**-MmpA3a and M214A appeared to corroborate this hypothesis as transfer occurred more readily. Although it is not clear why metabolite flux has been modulated, it does suggest that the non-elongating KS^0^ could be further engineered to increase metabolite yields.

Interestingly, the closely related thiomarinol class of hybrid antibiotics possess a marinolic acid polyketide backbone esterified to a C8 fatty acid that is in turn fused to a pyrrothine subunit. The thiomarinols lack the 10,11-epoxide, but nonetheless, the biosynthetic pathway possesses an equivalent module to MmpE, termed TmpE.^[29]^ Although the TmpE_OR domain is non-functional (and therefore the 10,11 alkene remains intact), the module still possesses a KS^0^ domain and integral ACP. Transfer of the equivalent pre-marinolic acid intermediate to the TmpE_ACP is therefore not to facilitate interaction with the OR but instead to set-up the next biosynthetic step, which may be common in nature and timing to both pathways. In addition, the N-terminal of TmpE is fused to TmlK, the ECH_2_ domain from the β-branching cassette (thiomarinol contains an equivalent C3 β-branch) and sequence analysis of the N-terminal revealed that there is no equivalent docking domain (Figure S2). The C-terminal of TmpA that follows an ACP tri-domain has a short stretch of amino acids that more closely resembles a C-terminal docking domain, but nonetheless is still predicted to be unstructured. Therefore, the homologous thiomarinol PKS likely follows a similar set of ACP transfers at this point, but is driven by a divergent set of protein-protein interactions and synthase assembly that remain to be delineated.

The discovery and characterisation of the MmpE_ACP and successful demonstration of epoxidation of **2**-MmpE_ACP by the class A FMO, MmpE_OR, has determined the timing of this key mechanistic step. Epoxidations are common biosynthetic events catalyzed as shown here, by FMOs, or cytochrome P450 monoxygenases. However, most often these intermediates are further processed to yield oxygen-containing heterocycles such as pyran rings and polyethers in polyketide biosynthesis.^[30]^ Epoxidations by class A FMOs have been reported but the biosynthetic examples remain limited.^[22]^ Epoxidation by *trans*-acting class A FMOs are involved in the assembly of the polyether antibiotics lasacolid (Lsd18)^[31]^ and monensin (MonCI),^[32]^ where epoxidation and subsequent epoxide-opening cascades generate polycyclic ethers. Further examples include the fungal indole alkaloids paraherquamide (PhqK)^[33]^ and fumiquinazolines (FqzB)^[34]^ where epoxidation precedes rearrangements that generate spiro-carbon centres that are critical for function. In each of these cases, the FMO function has been demonstrated using free substrate mimics alongside gene-knockouts although in the case of monensin the epoxidase substrate pre-monensin is proposed to be ACP-bound.^[32b]^

Persistent epoxides, as found in PA-A/B, where the epoxide ring is not lost from the polyketide, most often arise post-assembly and through the action of a *trans*-acting cytochrome P450, as in the examples of oleandomycin and epothilone.^[35]^ Online epoxidation, where the incomplete intermediate remains attached to an ACP and the epoxide group persists, is less common and even more challenging to study in vitro due to difficulties in accessing the true ACP-bound substrate. In this study we provide the first demonstration of efficient ‘online’ epoxidation of a complex native substrate attached to a specific ACP in vitro, with a single oxidation event and approximately 90 % conversion. The epoxidation occurs remotely (9 carbon atoms) from the thioester, an unusual feature that highlights the need for an authentic substrate to demonstrate efficient turnover. In fact, we demonstrate that the conversion efficiency is substrate length specific and a shorter curtailed 10-carbon backbone polyketide intermediate **15** was only partially modified, alongside which there was considerable back-ground substrate hydrolysis. Furthermore, our previous knockout studies of MmpD KR4 and C6 α-hydroxylase, MupA, all yielded a subset of products that lacked an epoxide, implying the presence of both the 6, 7-hydroxyl groups might promote efficient epoxidation by MmpE_OR. Given these difficulties, it is not surprising that to date, the role of online FMOs in biosynthetic pathways have relied on in vivo knockouts/feeding studies. For example this approach was applied to the study of SdlR, a single-component class B FMO from the shuangdaolide biosynthetic pathway that performs a remote 16,17-epoxidation (that subsequently induces formation of the key 2-hydroxycyclopentenone moiety; Figure 6B).^[26]^ However, this same feature confounded studies to demonstrate SdlR-catalyzed epoxidation in vitro as no authentic synthetic substrate was available. A further example of online epoxidation was demonstrated using in vivo knockout studies of the spliceostatin pathway from *Burkholderia sp*. FERM BP-3421 (Figure 6C).^[36]^ Here an *exo*-β-methylene is epoxidized via an *in-cis* FMO (sequence analysis indicated this is a class A FMO) with the substrate presumably tethered to the tandem ACP array of split module Fr9GH-Fr9I.^[37]^

The potential for the class A FMOs as epoxidase biocatalysts remains unexplored (versus class E), but this study has highlighted several interesting features that warrant further exploration. Initial work using feeding studies showed both desepoxy-PA-B and PA-C, which contain the THP ring and an esterified C_9_ fatty acid chain, failed to turnover. Mupirocin W5, which also incorporates an esterified C_7_ fatty acid chain, was epoxidized to a small degree (ca. 5 %) and in vitro the shortened polyketide chain **15** lacking the 6-OH also gave a similar degree of conversion. Therefore, although there is clearly tight control around the nature of the polyketide portion of the chain and a preference for linear polyketide substrates, the wild-type enzyme does show a degree of tolerance that could be exploited by e.g. directed evolution.

## Conclusions

MmpE_OR is therefore an interesting example of an epoxidase that acts online, is integrated into the modular PKS structure and modifies a site on the substrate nine carbon atoms away from the thioester. The confirmation of the MmpA-MmpE biosynthetic sequence and the timing of the epoxidation of the 10,11-alkene has now pinpointed the location of the polyketide chain prior to the final two modification steps on pathway to PA-B, namely formation of the THP ring and esterification with the hydroxylated fatty acid. In a series of in vitro studies, we have recently elucidated the mechanism for formation of the THP ring and assembly of the 9-HN moiety. Our in vitro evidence to date favours a mechanism by which the 9-HN is fully elongated and then esterified, with ring closure happening just prior or after this event. Knockout experiments and a re-feeding experiment have however also shown a mechanism by which shorter esterified chains are subsequently elongated is possible. Nonetheless, MmpE_ACP bearing the linear, epoxidized (or 10,11-alkene in PA-C) polyketide must be the correct partner enzyme for either the esterase (potentially MupB) or MupW/MupT/MupZ that form the THP ring. MmpE lacks a TE that might otherwise release the polyketide, suggesting it is off-loaded by an alternate mechanism, possibly during the esterification reaction and priming of MupB. The TE present on MmpB might then be responsible for release of PA-B (if cyclisation occurs prior to esterification) or the linear ester which is then ring closed as the free substrate.

## Supplementary Material

Supporting Information

## Data Availability

The data that support the findings of this study are available in the supplementary material of this article.
