# Supplementary material for: An Integrated Module Performs Selective ‘Online’ Epoxidation in the Biosynthesis of the Antibiotic Mupirocin
Source: Angew Chem Int Ed Engl. Author manuscript; Available in PMC 2026 May 6. (PMC7619045; doi:10.1002/anie.202410502)
Supplement: Supporting Information [file EMS213462-supplement-Supporting_Information.pdf]

Supporting Information

An integrated module performs selective 'on-line' epoxidation in the biosynthesis of the antibiotic mupirocin.

Ashley J Winter[a], Felix de Courcy-Ireland[a], Annabel P Phillips[a], Joseph M Barker[a], Nurfarhanim A Bakar[b], Nahida Akter[a,c], Luoyi Wang[d], Zhongshu Song[a], John Crosby[a], Christopher Williams[a], Christine L Willis[a], Matthew P Crump*[a]

[a] School of Chemistry, University of Bristol, Cantock's Close, Bristol, BS8 1TS, UK.

[b] Department of Engineering and Sciences, School of Liberal Arts and Sciences, Taylor's University, Subang Jaya, 47500 Selangor, Malaysia.

[c] Department of Chemistry, University of Barisal, Barisal-8200, Bangladesh.

[d] Institute of Microbiology, Chinese Academy of Sciences, NO. 1 Beichen West Road, Chaoyang District, Beijing 100101, China.

1. General Synthetic Experimental 3
   Scheme S1 4
2. Chemical Synthesis Experimental leading to **2**-pant 5
3. Plasmid generation, protein expression and purification. 21
4. Whole cell biotransformation with MmpE_OR 22
5. *In vitro* LCMS assays 22
6. ESMS Assays. 22
7. NMR Parameters 23
8. Generation of the mmpE M214A and C215A point mutations. 24

Figure S1 Identification of an ACP domain within MmpE 26

Figure S2 Bioinformatic analysis of candidate ^{C}DD and ^{N}DD docking domain regions with the Mupirocin and Thiomarinol gene clusters. 27

Figure S3 Characterisation of a docking domain between MmpA and MmpE. 30

Figure S4 Protein purification and characterisation. 31

Figure S5 Translocation of **1** and propionyl between MmpA3a and MmpE_KS. 33

Figure S6 MmpE_ACP upgrades for epoxidation assays 34

Figure S7 Metabolite isolation from P. fluorescens M214A and C215A 35

Figure S8 Characterisation of MmpE_ACP. 36

Figure S9 Multiple sequence alignment of MmpE_OR (uniprot: Q8RL60) with Class A Flavin dependent monooxygenases. 37

Figure S10 Epoxidation assays utilising MmpE_OR 38

Figure S11 Control reactions of the epoxidation reaction 2-MmpE_ACP by MmpE_OR 39

Figure S12 ^{1}H NMR spectrum of Mupirocin W2 40

Figure S13 Analysis of the X-Cys motif in Trans-AT KS domains immediately downstream of acceptor ACPs. 41

NMR spectra to support synthesis 42

Constructs used in this study 67

1. General Synthetic Experimental:

All reagents were sourced from commercial suppliers and were used without further purification unless stated otherwise. Anhydrous THF, Et_2O, hexane, and DCM were dried by passing through a modified Grubbs system of alumina columns, manufactured by Anhydrous Engineering. All stated temperatures below ambient are the temperatures of the cooling baths, unless otherwise stated. Flash column chromatography was performed using silica gel 60 (Fisher Scientific or Aldrich) and a suitable eluent. TLC analysis was performed with aluminium-backed silica TLC plates (Merck-Kieselgel 60 F254) with a suitable solvent system and was visualised using UV fluorescence (254 & 366 nm) and/or developed with potassium permanganate solution. Infrared (IR) spectra were recorded on a Perkin Elmer Spectrum Two FT-IR spectrometer; signals are reported as broad (br), strong (s), medium (m) and weak (w) relative to the most intense peak; frequencies are reported in wavenumbers (cm^{-1}). ^{1}H and ^{13}C NMR spectra were recorded using Jeol ECS 400 MHz, Varian 400-MR (400 MHz), Bruker Advance III HD 500 MHz (5mm cryo ^{13}C optimised TXO), Bruker *NEO* 600 MHz (custom built 5mm cryo ^{13}C, ^{15}N optimised TXO-$^{1}H^{19}F$) or Bruker *AVANCE* III HD 700 MHz (1.7 mm micro-cryo TCI probe) spectrometers. Chemical shifts (δ) are quoted in parts per million (ppm) and coupling constants (*J*) are in Hertz (Hz). Proton NMR multiplicities are reported as follows; s = singlet, d = doublet, t = triplet, q = quartet, m = multiplet, br = broad, or combinations thereof. Residual solvent peaks were used as the internal reference for proton and carbon chemical shifts. HRMS ESI were performed on either a Bruker Daltonics Apex 4, 7 Tesla FTICR, or microTOF II. Samples were submitted in DCM. Optical rotation was measured on a Bellingham and Stanley Ltd. ADP220 polarimeter and is quoted in (° ml) $(g\ dm)^{-1}$.

2. Scheme S1. Synthesis of Substrate (2-Pant):

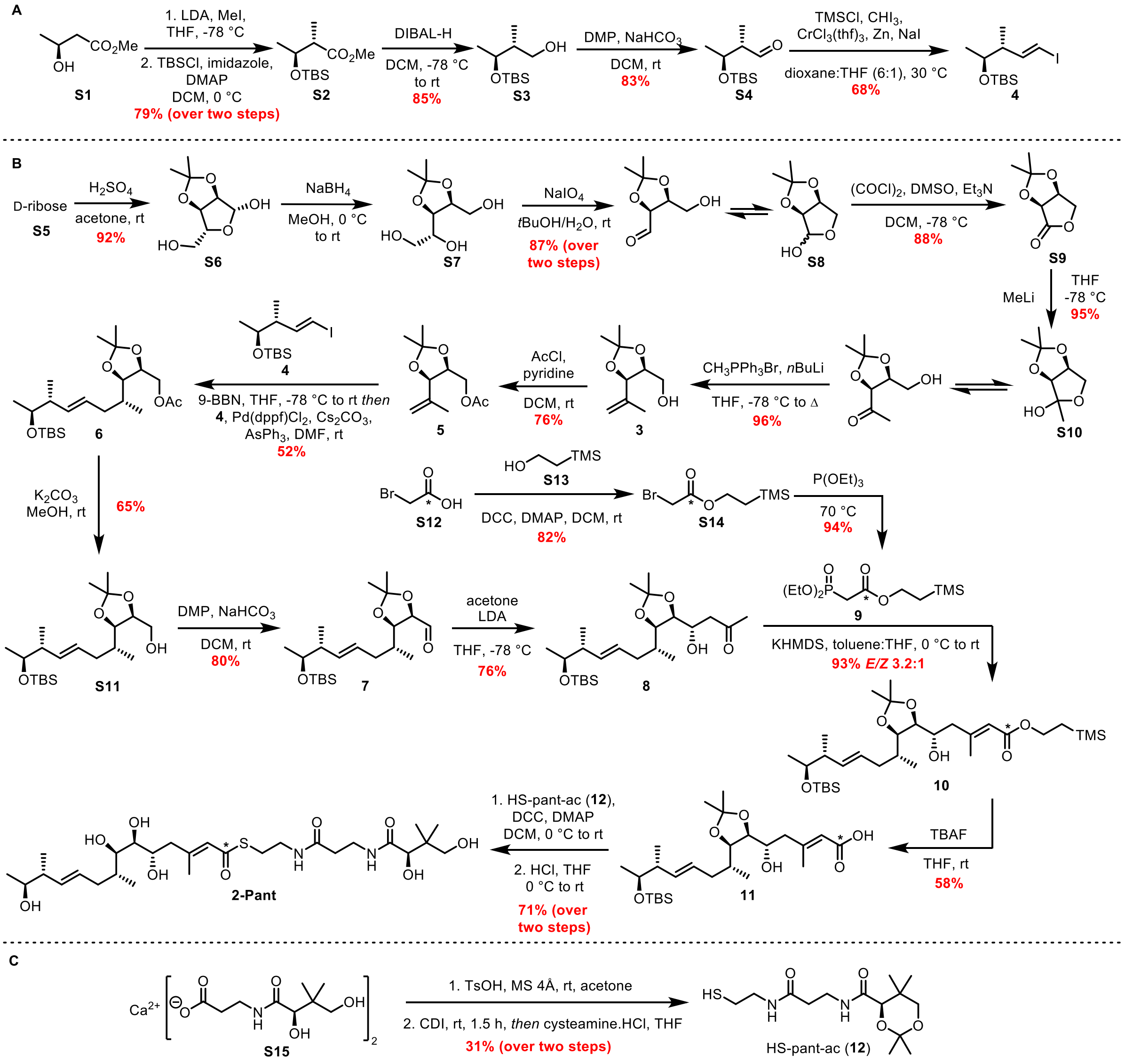

Scheme S1: The entire synthetic sequence leading to substrate 2-Pant. *(A) synthesis of vinyl iodide **4**; (B) the longest linear sequence starting from D-ribose (**S5**) including end-game manipulations leading to substrate **2**; (C) formation of protected pantetheine (**12**). ***** denotes a ^{13}C label that has been incorporated for future downstream esterification assays. 9-BBN = 9-borabicyclo[3.3.1]nonane, DCC = N,N'-dicyclohexylcarbodiimide, DIBAL-H = diisobutylaluminium hydride, DMAP = 4-(dimethylamino)pyridine, DMP = Dess-Martin periodinane, DMSO = dimethyl sulfoxide, KHMDS = potassium bis(trimethylsilyl)amide, LDA = lithium diisopropylamide, TBAF = tetrabutylammonium fluoride.*

Methyl (2*S*,3*S*)-3-(*tert*-butyldimethylsilyloxy)-2-methylbutanoate (**S2**):

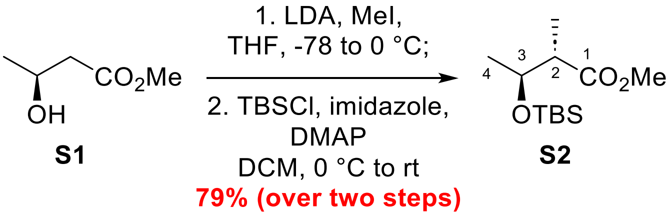

Methyl (*S*)-3-hydroxybutyrate (**S1**) (3.00 g, 25.4 mmol) in anhydrous THF (90 mL) was added dropwise to freshly prepared LDA solution [*n*BuLi (1.3 M in hexane, 39.9 mL, 53.1 mmol) was added to diisopropylamine (7.44 mL, 53.1 mmol) in anhydrous THF (30 mL) and stirred at 0 °C for 30 min] at 0 °C under nitrogen. The mixture was allowed to warm to rt and stirred for 20 min, after which it was cooled to –78 °C and iodomethane (1.95 mL, 31.4 mmol) was added dropwise. The reaction was stirred at 0 °C for 3 h and then quenched with aq. HCl (6 M, 30 mL). The layers were separated, and the aqueous layer washed with Et_2O (3 × 30 mL). The organic phases were combined, dried ($MgSO_4$), filtered, and concentrated *in vacuo*. The resulting residue was dissolved in anhydrous DCM (60 mL) and imidazole (3.00 g, 44.1 mmol) and DMAP (312 mg, 2.6 mmol) were added under nitrogen. The mixture was cooled to 0 °C and *tert*-butyldimethylsilyl chloride (4.58 g, 30.4 mmol) was added, after which the mixture was stirred at rt for 16 h. The reaction was quenched with sat. aq. $NaHCO_3$ solution (30 mL) and extracted with DCM (3 × 30 mL). The combined organic layers were dried ($MgSO_4$), filtered, and concentrated *in vacuo*. Purification by column chromatography (5% EtOAc in petroleum ether 40-60 °C) provided ester **S2** (4.92 g, 79% over two steps) as a yellow oil.

$[\alpha]_D^{24}$ +47 (*c* 1.25, $CHCl_3$), lit. $[\alpha]_D^{24}$ +42 (*c* 1.25, $CHCl_3$); δ_H (400 MHz, $CDCl_3$) 4.01 (1H, dq, *J* 7.2, 6.1, 3-H), 3.66 (3H, s, CO_2CH_3), 2.50 (1H, p, *J* 7.2, 2-H), 1.15 (3H, d, *J* 6.1, 4-H_3), 1.09 (3H, d, *J* 7.2, 2-CH_3), 0.86 (9H, s, $SiC(CH_3)_3$), 0.06 (3H, s, $SiCH_3$), 0.03 (3H, s, $SiCH_3$); δ_C (101 MHz, $CDCl_3$) 175.7 (C-1), 70.3 (C-3), 51.5 (OCH_3), 48.2 (C-2), 25.8 ($SiC(CH_3)_3$), 20.7 (C-4), 18.0 ($SiC(CH_3)_3$), 12.8 (2-CH_3), -4.2 ($SiCH_3$), -5.1 ($SiCH_3$).

Data are consistent with literature values.[1]

(2*R*,3*S*)-3-(*tert*-Butyldimethylsilyloxy)-2-methylbutan-1-ol (**S3**):

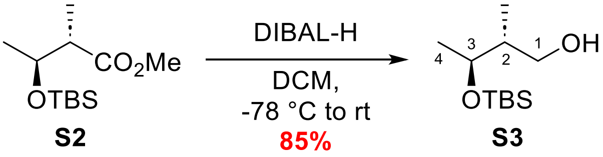

Ester **S2** (4.90 g, 19.9 mmol) was dissolved in anhydrous DCM (50 mL) and cooled to -78 °C under nitrogen. DIBAL-H (1.0 M in THF, 41.8 mL, 41.8 mmol) was added dropwise and the mixture was stirred at rt for 2 h. The reaction was quenched with sat. aq. Rochelle salt solution (80 mL) and H_2O (80

mL) at 0 °C and stirred vigorously for 2 h at rt until the solution separated into two layers. The aqueous layer was extracted with DCM (3 × 100 mL) and the combined organic layers were dried ($MgSO_4$), filtered, and concentrated *in vacuo*. Purification by column chromatography (20% EtOAc in petroleum ether 40-60 °C) provided alcohol **S3** (3.69 g, 85%) as a colourless oil.

$[\alpha]_D^{24}$ +19 (*c* 1.0, $CHCl_3$), lit. $[\alpha]_D^{24}$ +18 (*c* 1.0, $CHCl_3$); δ_H (400 MHz, $CDCl_3$) 3.84 – 3.73 (2H, m, 1-H*H* & 3-H), 3.55 (1H, dt, *J* 11.1, 5.7, 1-*H*H), 2.86 (1H, t, *J* 5.7, OH), 1.59 (1H, m, 2-H), 1.22 (3H, d, *J* 6.1, 4-H_3), 0.97 (3H, d, *J* 7.0, 2-CH_3), 0.90 (9H, s, $SiC(CH_3)_3$), 0.10 (3H, s, $SiCH_3$), 0.09 (3H, s, $SiCH_3$); δ_C (101 MHz, $CDCl_3$) 74.1 (C-3), 66.0 (C-1), 41.8 (C-2), 25.9 (SiC(*C*$H_3)_3$), 22.3 (C-4), 18.0 (Si*C*$(CH_3)_3$), 14.8 (2-CH_3), -4.1 ($SiCH_3$), -4.9 ($SiCH_3$).

Data are consistent with literature values.[1]

(2*S*,3*S*)-3-(*tert*-Butyldimethylsilyloxy)-2-methylbutanal (**S4**):

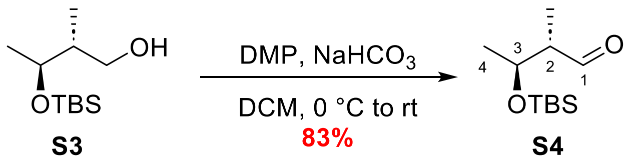

Alcohol **S3** (3.69 g, 16.9 mmol) was dissolved in DCM (80 mL) and DMP (9.29 g, 21.9 mmol) and $NaHCO_3$ (7.10 g, 84.5 mmol) were added at 0 °C. The reaction was warmed to rt, stirred for 2 h, and then quenched by addition of sat. aq. $Na_2S_2O_3$ solution (30 mL) and the aqueous layer was extracted with Et_2O (3 x 30 mL). The combined organic layers were dried ($MgSO_4$), filtered, and concentrated *in vacuo*. Purification by column chromatography (10% EtOAc in petroleum ether 40-60 °C) yielded aldehyde **S4** (3.03 g, 83%) as a colourless oil.

$[\alpha]_D^{24}$ +45 (*c* 1.0, $CHCl_3$), lit. $[\alpha]_D^{24}$ +47 (*c* 1.0, $CHCl_3$); δ_H (500 MHz, $CDCl_3$) 9.75 (1H, d, *J* 2.6, CHO), 4.03 (1H, m, 3-H), 2.37 (1H, qdd, *J* 7.0, 5.8, 2.6, 2-H), 1.22 (3H, d, *J* 6.3, 4-H_3), 1.07 (3H, d, *J* 7.0, 2-CH_3), 0.87 (9H, s, $C(CH_3)_3$), 0.07 (3H, s, $SiCH_3$), 0.05 (3H, s, $SiCH_3$); δ_C (126 MHz, $CDCl_3$) 205.4 (CHO), 70.0 (C-3), 53.8 (C-2), 25.9 (SiC(*C*$H_3)_3$), 21.9 (C-4), 18.1 (Si*C*$(CH_3)_3$), 10.8 (2-CH_3), -4.1 ($SiCH_3$), -4.9 ($SiCH_3$).

Data are consistent with literature values.[1]

(4*S*,3*R*,1*E*)-4-(*tert*-Butyldimethylsilyloxy)-1-iodo-3-methylpent-1-ene (**4**):

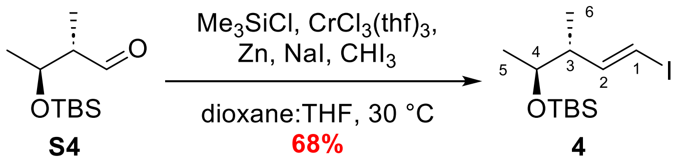

Me_3SiCl (3.0 mL, 23.6 mmol) was added to a well-stirred suspension of $CrCl_3(thf)_3$ (0.30 g, 0.80 mmol), freshly activated zinc* (1.56 g, 23.9 mmol) and dried NaI** (0.6 g, 4.0 mmol) in anhydrous

dioxane***:THF (40 mL; 6:1 dioxane:THF), the mixture was heated to 30 °C and stirred for 40 min under nitrogen. A solution of aldehyde **S4** (866 mg, 4.0 mmol) and CHI_3 (3.2 g, 8.1 mmol) in anhydrous dioxane:THF (40 mL; 6:1 dioxane:THF) was added to this mixture over 4 h under nitrogen. The resulting solution was stirred for a further 14 h during which the suspension turned red/brown and ^1H NMR showed complete consumption of starting material. The reaction was diluted with H_2O (100 mL), the aqueous layer was extracted with hexane (3 x 80 mL), and the combined organic layers were washed with sat. aq. $Na_2S_2O_3$ (150 mL), brine (100 mL), dried ($MgSO_4$), filtered, and concentrated *in vacuo*. Purification by column chromatography (hexane) gave iodide **4** (921 mg, 68%) as a yellow oil.

$[\alpha]_D^{22}$ +10 (*c* 1.0, $CHCl_3$), lit. $[\alpha]_D^{25}$ +17 (*c* 1.0, $CHCl_3$); δ_H ($CDCl_3$, 400 MHz) 6.48 (1H, dd, *J* 14.5, 8.6, 2-H), 5.99 (1H, dd, *J* 14.5, 0.9, 1-H), 3.65 (1H, qd, *J* 6.2, 4.9, 4-H), 2.18 (1H, m, 3-H), 1.07 (3H, d, *J* 6.2, 5-H_3), 0.98 (3H, d, *J* 6.8, 3-CH_3), 0.89 (9H, s, $SiC(CH_3)_3$), 0.04 (6H, s, ($Si(CH_3)_2$); δ_C ($CDCl_3$, 101 MHz) 149.2 (C-2), 74.9 (C-1), 71.3 (C-4), 48.5 (C-3), 26.0 ($SiC(\mathit{C}H_3)_3$), 21.4 (C-5), 18.2 ($Si\mathit{C}(CH_3)_3$), 15.9 (C-6), -4.2 ($Si(CH_3)_2$), -4.6 ($Si(CH_3)_2$); HRMS (EI) m/z: $[M - {}^tBu]^+$ calc for $C_{12}H_{25}OISi$ 283.0010, found 283.0008.

Data are consistent with literature values.[1]

* Zinc (10 g) was washed consecutively with 2 M HCl (3 x 30 mL), H_2O (30 mL), EtOH (30 mL), and Et_2O (30 mL), and then dried *in vacuo* for 2 h.
** NaI (10 g) was heated to 120 °C under reduced pressure overnight.
*** The dioxane was degassed prior to use by vigorously bubbling nitrogen through the solvent for 20 min.

Acetonide of (3*R*,4*S*,5*R*)-5-(hydroxymethyl)tetrahydrofuran-2,3,4-triol (**S6**):

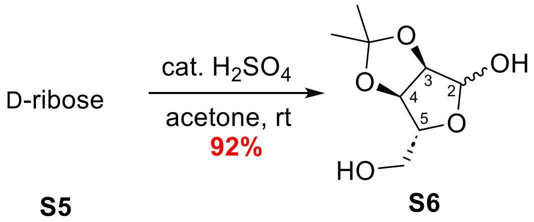

D-ribose (**S5**) (5.003 g, 33.3 mmol) was dissolved* in acetone (100 mL), conc. H_2SO_4 (0.15 mL) was added at rt and the reaction mixture stirred for 2 h until TLC showed complete conversion of the starting material. The reaction mixture was neutralised by addition of solid $NaHCO_3$ (15 g) and the mixture was filtered. The filtrate was concentrated** *in vacuo* to give a thick colourless oil. Purification by column chromatography (30% EtOAc: 70% petroleum ether 40-60 °C to 100 % EtOAc) provided acetonide **S6** (5.801 g, 92%) as a thick colourless oil.

δ_H (400 MHz, $CDCl_3$) 5.43 (1H, d, *J* 6.4, 2-H), 4.86 (1H, d, *J* 5.9, 4-H), 4.60 (1H, d, *J* 5.9, 3-H), 4.42 (1H, t, *J* 2.9, 5-H), 4.04 (1H, br m, 2-OH), 3.84 – 3.67 (2H, m, 6-H_2), 3.08 (1H, br m, 6-OH), 1.49 (3H, s, CH_3), 1.33 (3H, s, CH_3); δ_C (101 MHz, $CDCl_3$) 112.2 ($\mathit{C}(CH_3)_2$), 103.0 (C-2), 88.0 (C-5), 87.0 (C-3), 81.9 (C-4), 63.7 (C-6), 26.5 (CH_3), 24.9 (CH_3).

Data are consistent with literature values.[2]

* D-Ribose is sparingly soluble in acetone and gradually dissolved as the reaction proceeded.

** Care was taken not to remove all of the acetone to prevent the viscous crude product from becoming intractable and difficult to transfer.

Acetonide of (3*S*,4*S*)-tetrahydrofuran-3,4,5-triol (**S8**):

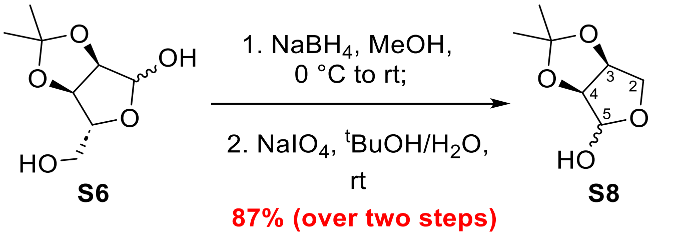

Acetonide **S6** (6.279 g, 33.0 mmol) was dissolved in dry MeOH (42 mL) and $NaBH_4$ (1.837 g, 49.5 mmol) was added in small portions at 0 °C under nitrogen and the resulting reaction mixture was then stirred at rt for 1 h. The solvent was removed *in vacuo*, *t*BuOH/H_2O (50/35 mL) and $NaIO_4$ (28.23 g, 132.0 mmol) were added, and the resulting reaction mixture was stirred at rt for 16 h. The reaction mixture was diluted with DCM (100 mL), neutralised by addition of aq. HCl (1 M), and filtered. The filtrate was extracted with DCM (3 x 100 mL), then the combined organic layers were dried ($MgSO_4$), filtered, and concentrated *in vacuo*. Purification by column chromatography (30% to 50% EtOAc in petroleum ether 40-60 °C) provided lactol **S8** (4.620 g, 87% over two steps) as a yellow oil.

δ_H (500 MHz, $CDCl_3$) 5.43 (1H, d, *J* 2.3, 5-H), 4.84 (1H, dd, *J* 5.9, 3.6, 3-H), 4.59 (1H, d, *J* 5.9, 4-H), 4.08 (1H, dd, *J* 10.4, 3.6, 2-*H*H), 4.03 (1H, d, *J* 10.4, 2-H*H*), 2.24 (1H, d, *J* 2.3, OH), 1.48 (3H, s, CH_3), 1.33 (3H, s, CH_3); δ_C (126 MHz, $CDCl_3$) 112.3 (*C*(CH_3)$_2$), 101.9 (C-5), 85.2 (C-4), 80.0 (C-3), 72.1 (C-2), 26.2 (CH_3), 24.8 (CH_3).

Data are consistent with literature values.[2]

Acetonide of (3*S*,4*S*)-3,4-dihydroxydihydrofuran-2(3H)-one (**S9**):

Method 1:

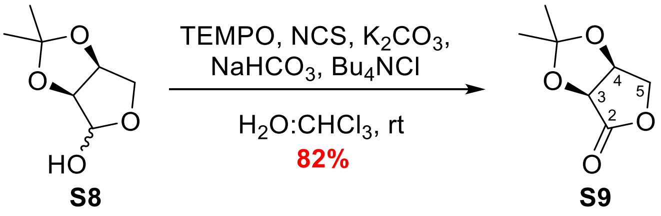

Lactol **S8** (4.293 g, 26.8 mmol) was dissolved in $CHCl_3$ (110 mL) and Bu_4NCl (745 mg, 2.7 mmol), TEMPO (419 mg, 2.7 mmol), $NaHCO_3$ (3.377 g, 40.2 mmol), a solution of K_2CO_3 (556 mg, 4.0 mmol) in H_2O (65 mL), and NCS (6.441 g, 48.2 mmol) were added, and the mixture stirred for 24 h. The reaction was quenched with sat. aq. $Na_2S_2O_3$ (100 mL) and the aqueous layer was extracted with Et_2O (3 × 100 mL). The combined organic layers were washed with brine (300 mL), dried ($MgSO_4$), filtered,

and concentrated *in vacuo*. Purification by column chromatography (30% EtOAc in petroleum ether 40-60 °C) gave lactone **S9** (3.486 g, 82%) as white solid.

$[\alpha]_D^{24}$ +110 (*c* 1.0, $CHCl_3$), lit. $[\alpha]_D^{24}$ +118 (*c* 1.0, $CHCl_3$); m.p. (from EtOH) 62 – 63 °C, lit. 64 °C; δ_H (400 MHz, $CDCl_3$) 4.87 (1H, ddd, *J* 5.7, 3.6, 0.9, 4-H), 4.74 (1H, d, *J* 5.7, 3-H), 4.45 (1H, dd, *J* 11.1, 0.9, 5-*H*H), 4.40 (1H, dd, *J* 11.1, 3.6, 5-H*H*), 1.47 (3H, d, *J* 0.8, CH_3), 1.38 (3H, d, *J* 0.8, CH_3); δ_C (101 MHz, $CDCl_3$) 174.3 (C-2), 114.1 (*C*(CH_3)$_2$), 75.6 (C-4), 74.7 (C-3), 70.3 (C-5), 26.9 (CH_3), 25.7 (CH_3).

Data are consistent with literature values.[3]

Method 2:

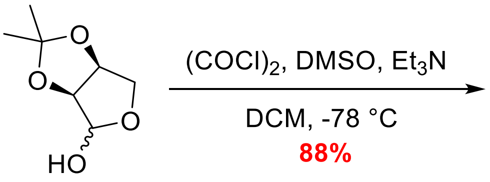

S8 **S9**

Oxalyl chloride (2.634 g, 1.78 mL, 20.8 mmol) was dissolved in DCM (20 mL) under nitrogen and cooled to –78 °C, DMSO (3.15 g, 2.86 mL, 40.3 mmol) was added dropwise and the mixture was stirred for 15 min. Lactol **S8** (2.151 g, 13.4 mmol) was dissolved in DCM (20 mL) and added to the mixture dropwise. After 1 h, triethylamine (4.07 g, 5.60 mL, 40.2 mmol) was added and the solution was warmed to rt. Sat. aq. $NaHCO_3$ (20 mL) was added, and the aqueous layer was extracted with Et_2O (3 x 20 mL), the combined organic layers were dried ($MgSO_4$), filtered, and concentrated *in vacuo*. Purification by column chromatography (30% EtOAc in petroleum ether 40-60 °C) gave lactone **S9** (1.861 g, 88%) as white solid.

Data as previously.

Acetonide of (3*S*,4*S*)-2-methyltetrahydrofuran-2,3,4-triol (**S10**):

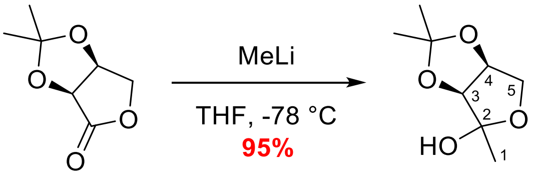

S9 **S10**

Lactone **S9** (3.265 g, 20.6 mmol) was dissolved in anhydrous THF (60 mL) and MeLi (15.4 mL, 24.6 mmol, 1.6 M) was added dropwise at -78 °C under nitrogen. The mixture was stirred for 3 h at -78 °C, then quenched with sat. aq. NH_4Cl (50 mL) and warmed to rt. The solvent was removed *in vacuo*, and the aqueous layer was extracted with Et_2O (3 x 30 mL), the combined organic layers were washed with brine (100 mL), dried ($MgSO_4$), filtered, and concentrated *in vacuo* to provide alcohol **S10** (3.398 g, 95%) as a white solid.

$[\alpha]_D^{24}$ +57 (*c* 1.00, $CHCl_3$), lit. $[\alpha]_D^{24}$ +57 (*c* 1.00, $CHCl_3$); m.p. 86 – 88 °C (from EtOH), lit. m.p. 82 – 85 °C; δ_H (500 MHz, $CDCl_3$) 4.86 (1H, dd, *J* 5.9, 4.0, 4-H), 4.41 (1H, d, *J* 5.9, 3-H), 4.02 (1H, dd, *J* 10.3, 4.0, 5-*H*H), 3.93 (1H, d, *J* 10.3, 5-H*H*), 1.95 (1H, br m, OH), 1.54 (3H, s, 2-CH_3), 1.49 (3H, s, C(C*H*$_3$)$_2$), 1.33 (3H, s, C(C*H*$_3$)$_2$); δ_C (126 MHz, $CDCl_3$) 112.6 (*C*(CH_3)$_2$), 106.2 (C-2), 85.1 (C-3), 81.0 (C-4), 71.2 (C-5), 26.5 (C(*C*H$_3$)$_2$), 25.1 (C(*C*H$_3$)$_2$), 22.6 (2-CH_3).

Data are consistent with literature values.[3a]

Acetonide of (2*S*,3*R*)-4-methylpent-4-ene-1,2,3-triol (**3**):

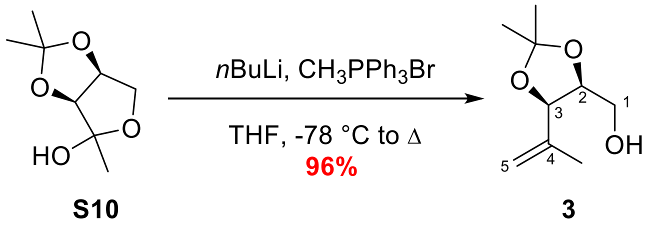

Methyltriphenylphosphonium bromide (28.29 g, 79.2 mmol) was dissolved in anhydrous THF (100 mL) and *n*BuLi (2.0 M, 39.6 mL, 79.2 mmol) was added dropwise at −78 °C. The reaction was warmed to 0 °C and stirred for 30 min, then cooled back down to −78 °C. Lactol **S10** (4.60 g, 26.4 mmol) in anhydrous THF (30 mL) was added dropwise, and the reaction was stirred at the same temperature for 1 h, then heated to reflux for 24 h. The reaction was then cooled to rt and quenched with H_2O (40 mL). The phases were separated, and the aqueous layer was extracted with EtOAc (3 × 40 mL). The combined organic layers were washed with brine (100 mL), dried ($MgSO_4$), filtered, and concentrated *in vacuo*. Purification by column chromatography (40% EtOAc in petroleum ether 40-60 °C) afforded alkene **3** (4.383 g, 96%) as a colourless oil.

$[\alpha]_D^{22}$ -95 (*c* 1.0, $CHCl_3$), lit. $[\alpha]_D^{24}$ -86 (*c* 1.0, $CHCl_3$); δ_H (500 MHz, $CDCl_3$) 5.13 (1H, m, 5-*H*H), 4.97 (1H, m, 5-H*H*), 4.62 (1H, d, *J* 6.6, 3-H), 4.28 (1H, ddd, *J* 7.9, 6.6, 4.6, 2-H), 3.52-3.42 (2H, m, 1-H_2), 1.86 (1H, m, OH), 1.75 (3H, s, 4-CH_3), 1.52 (3H, s, C(C*H*$_3$)$_2$), 1.40 (3H, s, C(C*H*$_3$)$_2$); δ_C (126 MHz, $CDCl_3$) 139.6 (C-4), 112.3 (C-5), 108.9 (*C*(CH_3)$_2$), 79.3 (C-3), 77.8 (C-2), 62.3 (C-1), 27.9 (C(*C*H$_3$)$_2$), 25.6 (C(*C*H$_3$)$_2$), 20.3 (4-CH_3).

Data are consistent with literature values.[3a]

Acetonide of (2*S*,3*R*)-1-acetoxy-2,3-dihydroxy-4-methylpent-4-ene (**5**):

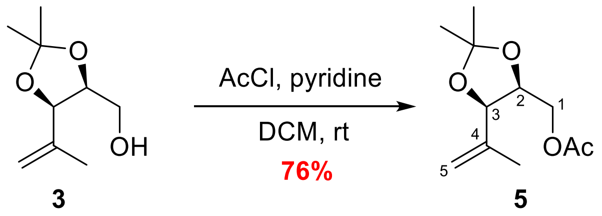

Alcohol **3** (4.46 g, 25.9 mmol) was dissolved in anhydrous DCM (100 mL) and stirred under nitrogen. Ethanoyl chloride (3.47 g, 3.15 mL, 44.1 mmol) and pyridine (2.72 g, 2.77 mL, 34.4 mmol) were added,

and the mixture was stirred for 2 h. The reaction mixture was quenched with addition of aq. HCl (1 M, 50 mL) and stirred for 10 min. The aqueous layer was washed with DCM (3 x 50 mL), dried ($MgSO_4$), filtered, and concentrated *in vacuo* to afford alkene **5** (4.213 g, 76%) as a colourless oil.

$[\alpha]_D^{24}$ -69 (*c* 1.0, $CHCl_3$); δ_H (400 MHz, $CDCl_3$) 5.14 (1H, m, 5-*H*H), 4.98 (1H, m, 5-H*H*), 4.63 (1H, d, *J* 6.6, 3-H), 4.40 (1H, ddd, *J* 8.0, 6.6, 4.2, 2-H), 4.05 (1H, dd, *J* 11.7, 4.2, 1-H*H*), 3.88 (1H, dd, *J* 11.7, 8.0, 1-*H*H), 2.07 (3H, s, C(=O)CH_3), 1.76 (3H, m, 4-CH_3), 1.53 (3H, s, C(CH_3)$_2$), 1.40 (3H, s, C(CH_3)$_2$); δ_C (126 MHz, $CDCl_3$) 171.0 (*C*(=O)CH_3), 139.3 (*C*(CH_3)$_2$), 113.0 (C-5), 109.2 (C-4), 79.5 (C-3), 75.4 (C-2), 64.2 (C-1), 27.8 (C(*C*H$_3$)$_2$), 25.6 (C(*C*H$_3$)$_2$), 21.1 (C(=O)*C*H$_3$), 20.3 (4-CH_3); IR (ν_{max}/cm^{-1}) (neat): 2986, 2941, 1710; HRMS (ESI/TOF) m/z: [M + Na]$^+$ calc for $C_{11}H_{18}O_4Na$ 237.1097, found 237.1085.

<u>Acetonide of (2*S*,3*R*,4*R*,8*R*,9*S*,*E*)-9-((*tert*-butyldimethylsilyl)oxy)-2,3-dihydroxy-4,8-dimethyldec-6-ene (**6**):</u>

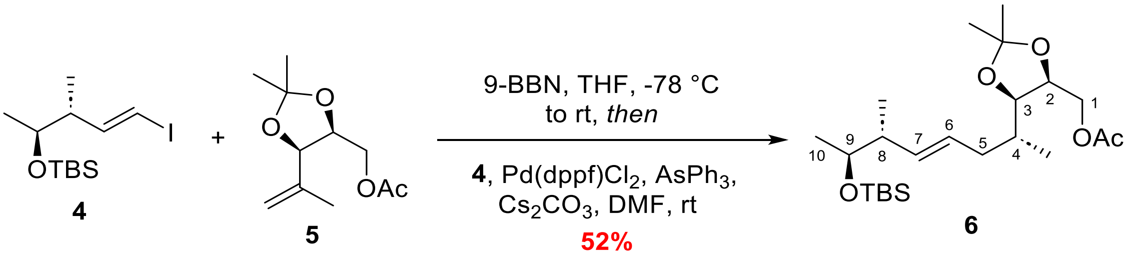

Alkene **5** (428 mg, 2.00 mmol) was dissolved in anhydrous THF (10 mL) and a solution of 9-BBN (8 mL, 4.0 mmol, 0.5 M in THF) was added dropwise at -78 °C under nitrogen. The reaction was stirred at rt for 16 h and was then quenched with degassed H_2O (10 mL) and stirred for 1 h. Iodide **4** (564 mg, 1.66 mmol), Cs_2CO_3 (1.95 g, 6.0 mmol), Pd(dppf)Cl_2 (292 mg, 0.4 mmol), $AsPh_3$ (122 mg, 0.4 mmol) were dissolved in degassed DMF (14 mL) under nitrogen and stirred for 10 min. The organoborane solution was then added dropwise to this mixture and the mixture turned black. The reaction was stirred for 16 h and then H_2O (15 mL) was added. The aqueous layer was extracted with Et_2O (3 × 10 mL), the combined organic layers were dried ($MgSO_4$), filtered, and concentrated *in vacuo*. Purification by column chromatography (0 - 15% EtOAc in petroleum ether 40-60 °C) gave alkene **6** (368 mg, 52%) as a yellow oil.

$[\alpha]_D^{24}$ -23 (*c* 1.0, $CHCl_3$); δ_H (500 MHz, $CDCl_3$) 5.43 (1H, dd, *J* 15.6, 7.7, 6-H), 5.38 (1H, m, 7-H), 4.26 – 4.16 (2H, m, 2-H & 1-*H*H), 4.00 (1H, dd, *J* 10.9, 7.7, 1-H*H*), 3.84 (1H, dd, *J* 10.4, 5.0, 3-H), 3.70 (1H, qd, *J* 6.2, 4.0, 9-H), 2.38 (1H, m, 5-*H*H), 2.15 (1H, qd, *J* 6.9, 4.0, 8-H), 2.10 (3H, s, C(=O)CH_3), 1.94 (1H, dt, *J* 13.4, 7.7, 5-H*H*), 1.70 (1H, m, 4-H), 1.46 (3H, s, C(CH_3)$_2$), 1.35 (3H, s, C(CH_3)$_2$), 1.03 (3H, d, *J* 6.2, 10-H_3), 0.97 (3H, d, *J* 6.9, 8-CH_3), 0.90 (3H, d, *J* 6.6, 4-CH_3), 0.88 (9H, s, SiC(CH_3)$_3$), 0.03 (6H, s, Si(CH_3)$_2$); δ_C (126 MHz, $CDCl_3$) 171.2 (*C*(=O)CH_3), 135.5 (C-7), 126.6 (C-6), 108.5 (*C*(CH_3)$_2$), 81.2 (C-3), 75.4 (C-2), 72.1 (C-9), 63.9 (C-1), 44.4 (C-8), 37.1 (C-5), 32.4 (C-4), 28.5 (C(*C*H$_3$)$_2$), 26.03 (SiC(*C*H$_3$)$_3$), 25.97 (C(*C*H$_3$)$_2$), 21.1 (C(=O)*C*H$_3$), 20.8 (C-10), 18.3 (Si*C*(CH_3)$_3$), 16.2 (8-CH_3), 15.8 (4-CH_3), -4.2 (Si(CH_3)$_2$), -4.7 (Si(CH_3)$_2$); IR (ν_{max}/cm^{-1}) (neat): 2960, 2934, 2856, 1744, 1700; HRMS (ESI/TOF) m/z: [M + Na]$^+$ calc for $C_{23}H_{44}O_5NaSi$ 451.2850, found 451.2869.

Acetonide of (2*S*,3*R*,4*R*,8*R*,9*S*,*E*)-9-((*tert*-butyldimethylsilyl)oxy)-4,8-dimethyldec-6-ene-1,2,3-triol (**S11**):

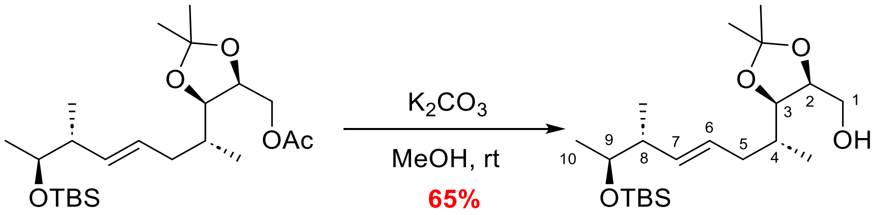

6 **S11**

Acetate **6** (86 mg, 0.20 mmol) was dissolved in MeOH (10 mL) and K_2CO_3 (276 mg, 2.0 mmol) was added at rt. After 2 h, H_2O (25 mL) was added and the aqueous layer was washed with EtOAc (3 x 25 mL), the combined organic layers were dried ($MgSO_4$), filtered, and the solvent removed *in vacuo*. Purification by column chromatography (15% EtOAc in petroleum ether 40-60 °C) gave alcohol **S11** (50 mg, 65%) as a colourless oil.

$[\alpha]_D^{24}$ -16 (*c* 1.0, $CHCl_3$); δ_H (500 MHz, $CDCl_3$) 5.43 (1H, m, 7-H), 5.30 (1H, m, 6-H), 4.12 (1H, m, 2-H), 3.84 (1H, dd, *J* 10.5, 5.4, 3-H), 3.69 (1H, qd, *J* 6.2, 4.0, 9-H), 3.64 – 3.54 (2H, m, 1-H_2), 2.37 (1H, m, 5-*H*H), 2.13 (1H, m, 8-H), 1.92 (2H, m, 5-H*H* & OH), 1.68 (1H, m, 4-H), 1.48 (3H, s, $C(CH_3)_2$), 1.36 (3H, s, $C(CH_3)_2$), 1.03 (3H, d, *J* 6.2, 10-H_3), 0.96 (3H, d, *J* 6.9, 8-CH_3), 0.88 (9H, s, $C(CH_3)_3$), 0.87 (3H, d, *J* 6.7, 4-CH_3), 0.03 (6H, s, $Si(CH_3)_2$); δ_C (126 MHz, $CDCl_3$) 135.4 (C-7), 126.7 (C-6), 108.3 ($C(CH_3)_2$), 81.1 (C-2), 77.9 (C-3), 72.1 (C-9), 61.7 (C-1), 44.4 (C-8), 37.1 (C-5), 32.0 (C-4), 28.8 ($C(CH_3)_2$), 26.0 ($SiC(CH_3)_3$), 26.0 ($C(CH_3)_2$), 20.8 (C-10), 18.3 ($SiC(CH_3)_3$), 16.2 (8-CH_3), 15.8 (4-CH_3), -4.2 ($SiCH_3$), -4.7 ($SiCH_3$).

Data are consistent with literature values.[3c]

Acetonide of (2*R*,3*R*,4*R*,8*R*,9*S*,*E*)-9-((*tert*-butyldimethylsilyl)oxy)-2,3-dihydroxy-4,8-dimethyldec-6-enal (7):

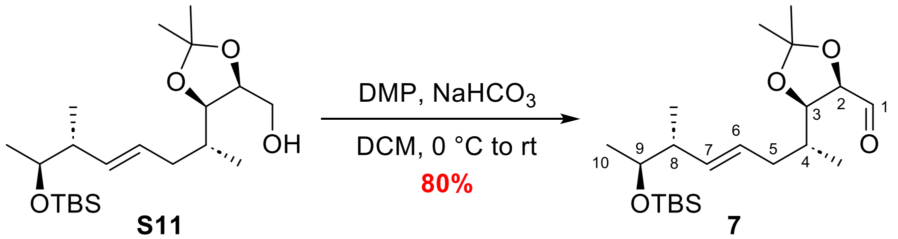

S11 **7**

Alcohol **S11** (50 mg, 0.13 mmol) was dissolved in DCM (8 mL) and DMP (64 mg, 0.15 mmol) and $NaHCO_3$ (54 mg, 0.64 mmol) were added at 0 °C. The reaction was stirred for 2 h and then warmed to rt, after which sat. aq. NaS_2O_3 solution (10 mL) was added and the aqueous layer was washed with DCM (3 x 15 mL), dried ($MgSO_4$), filtered, and concentrated *in vacuo*. Purification by column chromatography (30% EtOAc in petroleum ether 40-60 °C) gave aldehyde **7** (40 mg, 80%) as a clear, colourless oil.

$[\alpha]_D^{24}$ -18 (*c* 1.0, $CHCl_3$); δ_H (500 MHz, $CDCl_3$) 9.64 (1H, d, *J* 4.2, 1-H), 5.43 (1H, m, 7-H), 5.32 (1H, m, 6-H), 4.23 (1H, dd, *J* 6.4, 4.2, 2-H), 4.03 (1H, dd, *J* 10.1, 6.4, 3-H), 3.68 (1H, qd, *J* 6.2, 4.0, 9-H), 2.33 (1H, m, 5-H*H*), 2.12 (1H, m, 8-H), 1.95 (1H, m, 5-*H*H), 1.74 (1H, m, 4-H), 1.59 (3H, s, $C(CH_3)_2$), 1.41 (3H, s, $C(CH_3)_2$), 1.03 (3H, d, *J* 6.2, 10-H_3), 0.96 (3H, d, *J* 6.9, 8-CH_3), 0.91 (3H, d, *J* 6.7, 4-CH_3), 0.88 (9H, s, $SiC(CH_3)_3$), 0.03 (6H, s, Si(C*H*$_3$)$_2$); δ_C (126 MHz, $CDCl_3$) 201.2 (C-1), 135.7 (C-7), 126.3 (C-6), 110.6 (*C*(CH_3)$_2$), 83.0 (C-3), 82.0 (C-2), 72.0 (C-9), 44.4 (C-8), 36.9 (C-5), 32.7 (C-4), 28.0 (C(*C*H$_3$)$_2$), 26.0 (C(*C*H$_3$)$_3$), 25.6 (C(*C*H$_3$)$_2$), 20.8 (C-10), 18.3 (*C*(CH_3)$_3$), 16.3 (8-CH_3), 15.0 (4-CH_3), -4.2 ($SiCH_3$), -4.7 ($SiCH_3$); IR (ν_{max}/cm^{-1}) (neat): 2961, 2935, 2891, 2861, 1739, 1466, 1385; HRMS (ESI/TOF) m/z: $[M + Na]^+$ calc for $C_{21}H_{40}O_4NaSi$ 407.2594, found 407.2592.

Acetonide of (4*S*,5*S*,6*R*,7*R*,11*R*,12*S*,*E*)-12-((*tert*-butyldimethylsilyl)oxy)-4,5,6-trihydroxy-7,11-dimethyltridec-9-en-2-one (**8**):

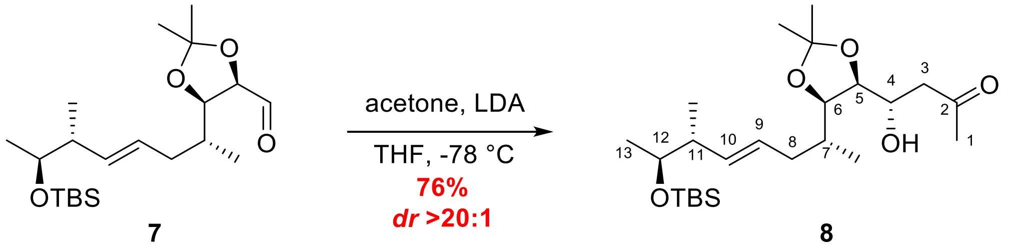

7 **8**

LDA solution made following general procedure A [*n*BuLi (1.11 M in anhydrous THF, 1.09 mL, 1.21 mmol) was added to diisopropylamine (178 μL, 1.28 mmol) in anhydrous THF (10 mL)] except the mixture was stirred for 30 min at -78 °C instead of 0 °C. Acetone (82 μL, 1.11 mmol) was added and the mixture stirred for 20 min. Aldehyde **7** (330 mg, 0.86 mmol) was dissolved in anhydrous THF (~5 mL) under nitrogen and added to the reaction mixture and stirred for 1 h at -78 °C. The reaction mixture was quenched by addition of sat. aq. NH_4Cl (10 mL), stirred for 10 min, and warmed to rt, before being washed with Et_2O (4 x 25 mL), and the combined organic layers were dried ($MgSO_4$), filtered, and concentrated *in vacuo*. The crude material was purified by column chromatography (20 to 50% EtOAc in petroleum ether 40-60 °C) to afford methyl ketone **8** (290 mg, 76%) as a clear oil.

$[\alpha]_D^{23}$ -64 (*c* 1.0, $CHCl_3$); δ_H (500 MHz, $CDCl_3$) 5.46 – 5.32 (2H, m, 10 & 9-H), 4.15 (1H, m, 4-H), 3.90 (1H, dd, *J* 8.3, 5.2, 5-H), 3.85 (1H, dd, *J* 9.0, 5.2, 6-H), 3.70 (qd, *J* 6.2, 4.1, 12-H), 3.14 (1H, d, *J* 4.7 Hz, OH), 2.91 (1H, dd, *J* 18.2, 2.4, 3-*H*H), 2.68 (1H, dd, *J* 18.2, 8.8, 3-H*H*), 2.34 (1H, m, 8-*H*H), 2.20 (3H, s, 1-H_3), 2.14 (1H, m, 11-H), 1.99 – 1.90 (2H, m, 7-H & 8-H*H*), 1.38 (3H, s, $C(CH_3)_2$), 1.32 (3H, s, $C(CH_3)_2$), 1.03 (3H, d, *J* 6.2, 13-H_3), 0.97 (3H, d, *J* 3.4, 7-CH_3), 0.96 (3H, d, *J* 4.2, 11-CH_3), 0.88 (9H, s, $SiC(CH_3)_3$)), 0.03 (6H, s, $Si(CH_3)_2$); δ_C (126 MHz, $CDCl_3$) 211.0 (C-2), 134.9 (C-10), 127.4 (C-9), 107.4 (*C*(CH_3)$_2$), 82.3 (C-6), 78.9 (C-5), 72.1 (C-12), 66.5 (C-4), 46.9 (C-3), 44.4 (C-11), 37.0 (C-8), 32.0 (C-7), 31.1 (C-1), 27.9 (C(*C*H$_3$)$_2$), 26.0 (SiC(*C*H$_3$)$_3$), 25.6 (C(*C*H$_3$)$_2$), 20.6 (C-13), 18.3 (Si*C*(CH_3)$_3$), 16.8 (7-CH_3), 16.1 (11-CH_3), -4.2 ($Si(CH_3)_2$), -4.7 ($Si(CH_3)_2$); IR (ν_{max}/cm^{-1}) ($CHCl_3$): 3480, 2959, 2932, 2860, 1711; HRMS (ESI/TOF) m/z: $[M + Na]^+$ calc for $C_{24}H_{46}O_5Si$ 465.3007, found 465.3001.

(2'-trimethylsilyl)ethyl [1-^{13}C]-2-bromoacetate (**S14**):

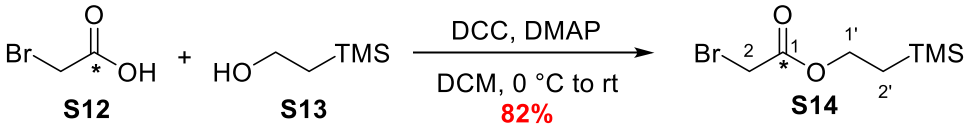

Acid **S12** (1.00 g, 7.2 mmol) was dissolved in anhydrous DCM (60 mL) and cooled to 0 °C under nitrogen. Alcohol **S13** (2.06 mL, 1.703 g, 14.4 mmol) and DMAP (86 mg, 0.7 mmol) were added, followed by portion-wise addition of DCC (1.568 g, 7.6 mmol). The reaction mixture was stirred for 24 h before being filtered through celite and washed (100 mL of 20% EtOAc: 80% petroleum ether 40-60 °C). The filtrate was then washed with sat. aq. $NaHCO_3$ (150 mL), H_2O (150 mL), brine (150 mL), dried ($MgSO_4$), filtered, and concentrated *in vacuo*. The crude material was purified by column chromatography (neat petroleum ether 40-60 °C to 50% EtOAc: 50% petroleum ether 40-60 °C) to afford ester **S14** (1.406 g, 82%) as a clear oil.

δ_H (600 MHz, $CDCl_3$) 4.30 – 4.24 (2H, m, 1'-H_2), 3.81 (2H, d, *J* 4.6, 2-H_2), 1.06 – 1.01 (2H, m, 2'-H_2), 0.05 (9H, s, $Si(CH_3)_3$); δ_C (151 MHz, $CDCl_3$) 167.5 (C-1), 64.9 (d, *J* 2.5, C-1'), 26.2 (d, *J* 65.0, C-2), 17.4 (d, *J* 1.8, C-2'), -1.4 ($Si(CH_3)_3$); IR (ν_{max}/cm^{-1}) ($CHCl_3$): 2956, 2120, 1693; HRMS was not obtained for this compound – it did not spray sufficiently to obtain any data.

(2'-trimethylsilyl)ethyl [1-^{13}C]-2-diethoxyphosphonoacetate (**9**):

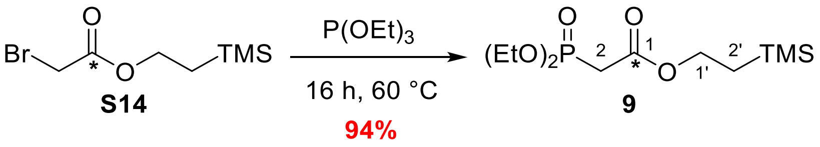

Ester **S14** (1.406 g, 5.9 mmol) and triethylphosphite (1.16 mL, 6.8 mmol) were heated to 60 °C under nitrogen for 16 h. The reaction mixture was then purified by column chromatography (neat EtOAc – neat acetone) to afford phosphonate **9** (1.641 g, 94%) as a clear oil.

δ_H (600 MHz, $CDCl_3$) 4.24 – 4.11 (6H, m, $(CH_3CH_2)_2$ & 1'-H_2), 2.98 – 2.90 (2H, dd, *J* 21.5, 7.4, 2-H_2), 1.34 (6H, t, *J* 7.1, $(CH_3CH_2)_2$), 1.05 – 0.98 (2H, m, 2'-H_2), 0.04 (9H, s, $Si(CH_3)_3$); δ_C (151 MHz, $CDCl_3$) 166.1 (d, *J* 6.2, C-1), 64.1 (d, *J* 2.5, C-1'), 62.8 (d, *J* 6.2, OCH_2CH_3), 34.6 (dd, *J* 134.4, 58.4, C-2), 17.5 (d, *J* 1.8, C-2'), 16.5 (d, *J* 6.2, OCH_2CH_3), -1.4 ($Si(CH_3)_3$); δ_P (162 MHz, $CDCl_3$, P(=O)Cy_3 reference = 50.20 ppm)) 19.4 (d, *J* 6.2); IR (ν_{max}/cm^{-1}) ($CHCl_3$) 2985, 2955, 2902, 1693, 1250; HRMS (ESI/TOF) m/z: $[M + Na]^+$ calc for $^{13}CC_{10}H_{25}O_5NaSiP$ 320.1141, found 320.1126.

Acetonide of 2'-(trimethylsilyl)ethyl ([1-^{13}C]-2*E*,5*S*,6*S*,7*R*,8*R*,10*E*,12*R*,13*S*)-13-((*tert*-butyldimethylsilyl)oxy)-5,6,7-trihydroxy-3,8,12-trimethyltetradeca-2,10-dienoate (**10**):

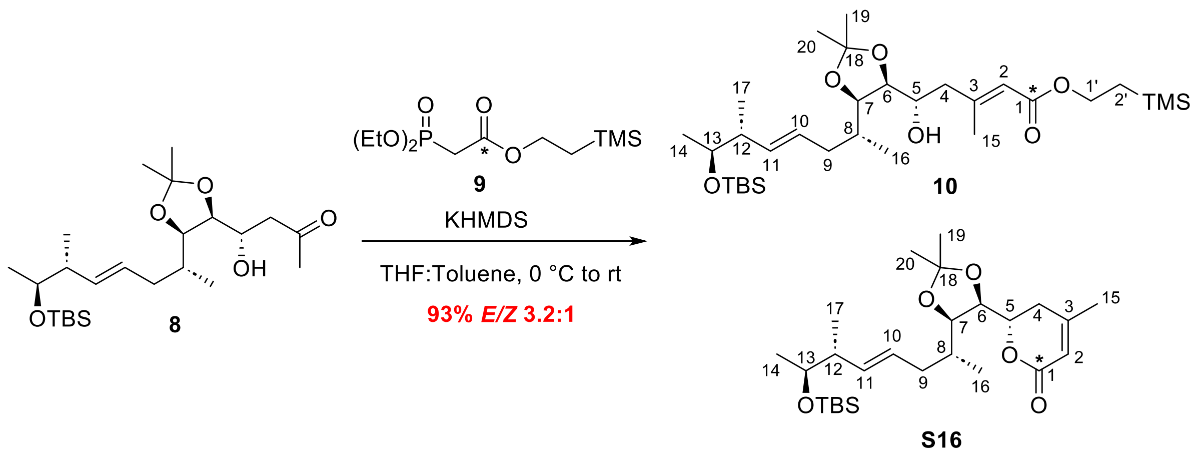

Phosphonate **9** (312 mg, 1.05 mmol) was stirred in anhydrous THF (3 mL) at 0 °C under nitrogen, KHMDS in toluene (0.5 M, 2.1 mL, 1.05 mmol) was added and the mixture was stirred for 1 h. Ketone **8** (136 mg, 0.31 mmol) was dissolved in anhydrous THF (3 mL) and added to the reaction mixture, which was warmed to rt and was left to stir for 16 h. The reaction mixture was quenched with H_2O (10 mL), and the THF was removed *in vacuo*. The aqueous layer was extracted with EtOAc (3 x 15 mL), the combined organic layers were washed with brine (45 mL), dried ($MgSO_4$), filtered, and concentrated *in vacuo*. The crude material was purified by column chromatography (10% EtOAc: 90% petroleum ether 40-60 °C to neat EtOAc) to afford *E* alkene **10** (126 mg, 0.22 mmol, 70%) as a clear oil.

$[\alpha]_D^{19}$ -4.0 (*c* 1.0, $CHCl_3$); δ_H (600 MHz, $CDCl_3$) 5.74 (1H, s, 2-H), 5.43 (1H, m, 11-H), 5.37 (1H, m, 10-H), 4.19 (2H, m, 1'-H_2), 3.90 – 3.84 (3H, m, 5-H, 6-H & 7-H), 3.70 (1H, qd, *J* 6.1, 4.1, 13-H), 2.72 (1H, d, *J* 13.8, 4-H*H*), 2.36 (1H, dd, *J* 10.8, 6.3, 9-H*H*), 2.21 (3H, s, 3-CH_3), 2.18 – 2.11 (2H, m, 4-*H*H & 12-H), 2.00 – 1.91 (2H, m, 9-*H*H & 8-H), 1.43 (3H, s, $C(CH_3)_2$), 1.34 (3H, s, $C(CH_3)_2$), 1.03 (3H, d, *J* 6.1, 14-H_3), 1.03 – 0.99 (2H, m, OCH_2CH_2Si), 0.99 – 0.95 (6H, m, 8-CH_3 & 12-CH_3), 0.04 (9H, s, $C(CH_3)_3$), 0.03 (6H, s, $Si(CH_3)_2$); δ_C (151 MHz, $CDCl_3$) 166.5 (C-1), 156.1 (d, *J* 1.9, C-3), 135.0 (C-11), 127.2 (C-10), 119.1 (d, *J* 75.0, C-2), 107.6 (*C*$(CH_3)_2$), 82.3 (C-7), 80.0 (C-5), 72.1 (C-13), 67.8 (C-6), 62.0 (d, *J* 2.5, OCH_2CH_2Si), 46.0 (d, *J* 7.1, C-4), 44.4 (C-12), 37.1 (C-9), 32.1 (C-8), 27.9 (C-19 or C-20), 25.9 (SiC(CH_3)$_3$), 25.5 (C-19 or C-20), 20.7 (C-14), 19.0 (C-15), 18.3 (Si*C*$(CH_3)_3$), 17.5 (d, *J* 1.8, OCH_2CH_2Si), 16.7 (C-16), 16.1 (C-17), -1.3 ($Si(CH_3)_3$), -4.2 ($Si(CH_3)_2$), -4.7 ($Si(CH_3)_2$); IR (ν_{max}/cm^{-1}) ($CHCl_3$) 3493, 2958, 2934, 2860, 1673; HRMS (ESI/TOF) m/z: $[M + Na]^+$ calculated for $^{13}CC_{30}H_{60}O_6NaSi_2$ 608.3860, found 608.4980.

The *Z* isomer of the alkene was not isolated. Under the reaction conditions, it cyclised to form lactone **S16** (33 mg, 23%) as a clear oil, the data for which is presented below:

$[\alpha]_D^{23}$ -32.0 (*c* 1.0, $CHCl_3$); δ_H (400 MHz, $CDCl_3$) 5.79 (1H, m, 2-H), 5.47 – 5.31 (2H, m, 10-H & 11-H), 4.48 (1H, m, 5-H), 4.12 (1H, m, 6-H), 3.89 (1H, dd, *J* 9.5, 5.0, 7-H), 3.69 (1H, qd,

J 6.2, 4.0, 13-H), 2.50 (1H, dd, J 18.3, 10.3, 4-*H*H), 2.38 (1H, m, J 4.4, 4-H*H*), 2.37 – 2.26 (2H, m, 9-*H*H), 2.13 (1H, m, 12-H), 1.99 (3H, s, 15-H_3), 1.95 (1H, m, 9-H*H*), 1.86 (1H, m, 8-H), 1.40 (3H, s, 19-H_3 or 20-H_3), 1.34 (3H, s, 19-H_3 or 20-H_3), 1.02 (3H, d, J 6.2, 14-H_3), 1.02 (3H, d, J 6.3, 16-H_3), 0.95 (3H, d, J 6.8, 17-H_3), 0.88 (9H, s, $C(CH_3)_3$), 0.02 (6H, s, $Si(CH_3)_2$); δ_C (101 MHz, $CDCl_3$) 164.1 (C-1), 157.5 (C-3), 135.2 (C-10), 126.9 (C-11), 116.4 (d, J 68.8, C-2), 107.9 (C-18), 82.1 (C-7), 77.5 (C-6), 74.8 (C-5), 72.1 (C-13), 44.4 (C-12), 37.0 (C-9), 31.8 (C-8), 31.6 (d, J 4.4, C-4), 27.9 (C-19 or C-20), 26.0 (C-19 or C-20), 25.4 ($C(CH_3)_3$), 23.3 (d, J 5.8, C-15), 20.6 (C-14), 18.2 ($C(CH_3)_3$), 16.6 (C-16), 16.0 (C-17), -4.3 ($Si(CH_3)_2$), -4.7 ($Si(CH_3)_2$); IR (ν_{max}/cm^{-1}) ($CHCl_3$) 3404 (weak), 3017, 2963, 2934, 2855, 1688; HRMS (ESI/TOF) m/z: $[M + Na]^+$ calculated for $^{13}CC_{25}H_{46}O_5NaSi$ 490.3046, found 490.3031.

Acetonide of [1-^{13}C]-(2*E*,5*S*,6*S*,7*R*,8*R*,10*E*,12*R*,13*S*)-13-((*tert*-butyldimethylsilyl)oxy)-5,6,7-trihydroxy-3,8,12-trimethyltetradeca-2,10-dienoic acid (**11**):

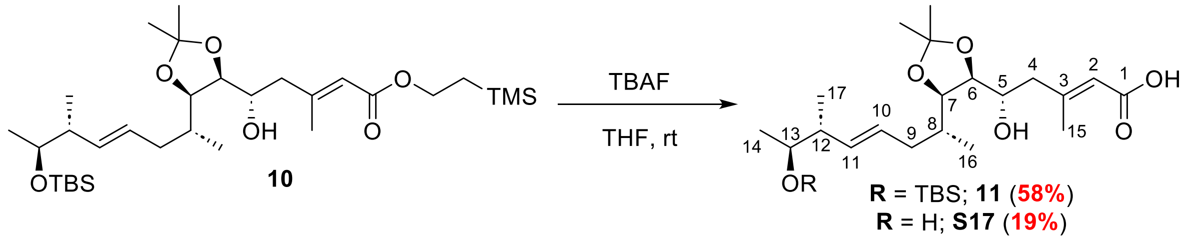

Ester **10** (126 mg, 0.22 mmol) was dissolved in anhydrous THF (3 mL) and stirred under nitrogen. TBAF (0.24 mmol, 0.24 mL, 1 M in THF) was added dropwise and the mixture was stirred for 3 h. TLC showed the presence of substantial amounts of starting material, and further TBAF (0.48 mL, 1 M in THF) was added and the mixture stirred for 16 h. The reaction mixture was quenched with HCl (10 mL, 0.5 M) and diluted with EtOAc (10 mL), and the layers separated. The aqueous layer was extracted with EtOAc (3 x 15 mL), the combined organics were washed with brine (50 mL), dried ($MgSO_4$), filtered, and concentrated *in vacuo*. The crude material was purified by column chromatography (40% to 60% EtOAc in petroleum ether 40-60 °C) to afford carboxylic acid **11** (61 mg, 58%) as a clear oil:

$[\alpha]_D^{23}$ +8.0 (*c* 1.0, $CHCl_3$); δ_H (600 MHz, $CDCl_3$) 5.80 (1H, s, 2-H), 5.44 (1H, m, 11-H), 5.37 (1H, m, 10-H), 3.89 (3H, m, 5-H, 6-H & 7-H), 3.70 (1H, qd, J 6.2, 4.1, 13-H), 2.75 (1H, d, J 13.9, 4-H*H*), 2.36 (1H, m, 9-H*H*), 2.23 (3H, s, 15-H_3), 2.19 (1H, m, 4-*H*H), 2.13 (1H, m, 12-H), 1.98 (1H, m, 9-*H*H), 1.93 (1H, m, 8-H), 1.44 (3H, s, $C(CH_3)_2$), 1.34 (3H, s, $C(CH_3)_2$), 1.03 (3H, d, J 6.2, 14-H_3), 0.98 (3H, m, 16-H_3), 0.97 (3H, m, 17-H_3), 0.88 (9H, s, $C(CH_3)_3$), 0.03 (6H, s, $Si(CH_3)_2$); δ_C (151 MHz, $CDCl_3$) 169.7 (C-1), 159.7 (d, J 1.9, C-3), 135.1 (C-11), 127.1 (C-10), 117.7 (d, J 73.1, C-2), 107.6 (C-18), 82.2 (C-7), 80.0 (C-6), 72.1 (C-13), 68.0 (C-5), 46.2 (d, J 6.8, C-4), 44.4 (C-12), 37.1 (C-9), 32.2 (C-8), 27.9 ($C(CH_3)_2$), 26.0 ($C(CH_3)_3$), 25.6 ($C(CH_3)_2$), 20.7 (C-14), 19.4 (C-15), 18.3 ($C(CH_3)_3$), 16.7 (C-16), 16.1

(C-17), -4.2 ($Si(CH_3)_2$), -4.7 ($Si(CH_3)_2$); IR (ν_{max}/cm^{-1}) ($CHCl_3$) 3462, 2963, 2963, 2930, 2856, 1663, 1634; HRMS (ESI/TOF) m/z: $[M + Na]^+$ calculated for $^{13}CC_{25}H_{48}O_6NaSi$ 508.3118, found 508.3134

and carboxylic acid **S17** (15 mg, 19%) as a clear oil

$[\alpha]_D^{20}$ -2.0 (*c* 1.0, $CHCl_3$); δ_H (600 MHz, $CDCl_3$) 5.78 (1H, s, 2-H), 5.54 (1H, m, 10-H), 5.37 (1H, dd, *J* 15.4, 8.5, 11-H), 3.95 – 3.85 (3H, m, 5-H, 6-H & 7-H), 3.52 (1H, m, 13-H), 2.73 (1H, d, *J* 13.9, 4-H*H*), 2.37 (1H, m, 9-H*H*), 2.21 (3H, s, 15-H_3), 2.18 (1H, m, 4-*H*H), 2.09 (1H, m, 12-H), 2.00 (1H, m, 9-*H*H), 1.98 (1H, m, 8-H), 1.43 (3H, s, 19-H_3 or 20-H_3), 1.33 (3H, s, 19-H_3 or 20-H_3), 1.16 (3H, d, *J* 6.2, 14-H), 0.99 (3H, d, *J* 6.7, 17-H_3), 0.99 (3H, d, *J* 6.1, 16-H_3); δ_C (151 MHz, $CDCl_3$) 170.7 (C-1), 159.4 (d, *J* 1.9, C-3), 134.3 (C-11), 130.2 (C-10), 118.1 (d, *J* 73.0, C-2), 107.7 (C-18), 82.3 (C-7), 80.0 (C-6), 71.3 (C-13), 67.9 (C-5), 46.2 (d, *J* 6.7, C-4), 45.3 (C-12), 37.1 (C-9), 32.0 (C-8), 27.9 (C-19 or C-20), 25.6 (C-19 or C-20), 20.2 (C-14), 19.4 (C-15), 17.0 (C-16), 16.8 (C-17); IR (ν_{max}/cm^{-1}) ($CHCl_3$) 3397, 2960, 2921, 2853, 1661, 1636; HRMS (ESI/TOF) m/z: $[M+H]^+$ calculated for $^{13}CC_{19}H_{35}O_6$ 372.2467, found 372.2421.

Diacetonide of [[1-^{13}C]-(2*E*,5*S*,6*S*,7*R*,8*R*,10*E*,12*R*,13*S*)-13-((*tert*-butyldimethylsilyl)oxy)-5,6,7-trihydroxy-3,8,12-trimethyltetradeca-2,10-dienoic acid][pantetheine] (**S18**):

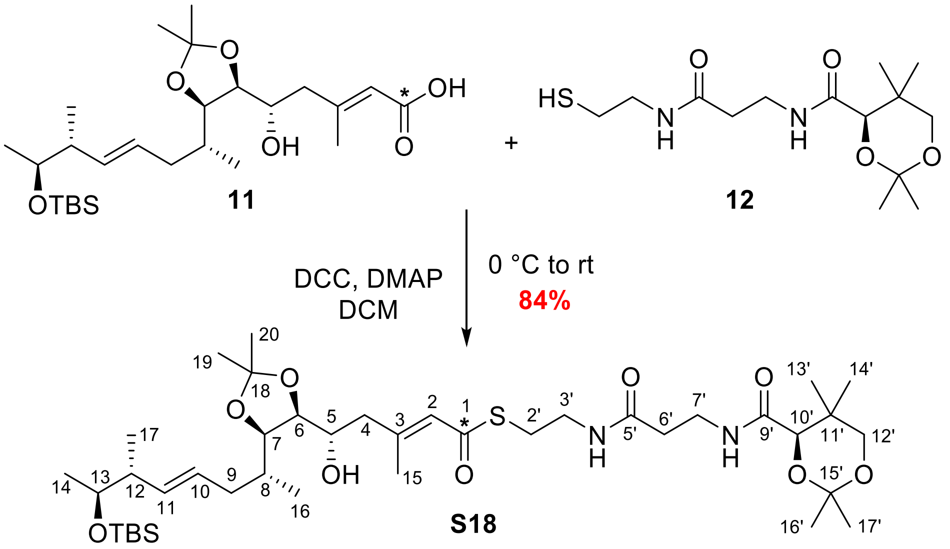

Carboxylic acid **11** (34 mg, 0.07 mmol) was dissolved in anhydrous DCM (2 mL), cooled to 0 °C and stirred under nitrogen. DCC (24 mg, 0.116 mmol) was added and the mixture was stirred for 5 min. Thiol **12** (27 mg, 0.085 mmol) and DMAP (26 mg, 0.213 mmol) were added and the mixture was warmed to rt and stirred for 16 h. The reaction mixture was quenched with sat. aq. NH_4Cl (10 mL), and the mixture was extracted with EtOAc (4 x 15 mL). The combined organic layers were dried (Na_2SO_4), filtered, and concentrated *in vacuo*. The crude material was purified by column chromatography (25%

EtOAc: 75% petroleum ether 40-60 °C to 10% MeOH: 90% EtOAc) to afford thioester **S18** (46 mg, 84%) as a clear oil.

$[\alpha]_D^{21}$ +24 (*c* 1.0, $CHCl_3$); δ_H (600 MHz, $CDCl_3$) 6.96 (1H, t, *J* 6.0, 8'-NH), 6.13 (1H, t, *J* 5.6, 4'-NH), 6.09 (1H, d, *J* 6.2, 2-H), 5.43 (1H, dd, *J* 15.5, 7.4, 11-H), 5.37 (1H, m, 10-H), 4.05 (1H, s, 10'-H), 3.92 – 3.85 (3H, m, 5-H, 6-H & 7-H), 3.70 (1H, m, 13-H), 3.67 (1H, d, *J* 11.7, 12'-H*H*), 3.59 (1H, m, 7'-H*H*), 3.52 (1H, dq, *J* 11.8, 5.8, 3'-H*H*), 3.45 (1H, m, 7'-*H*H), 3.41 (1H, m, 3'-*H*H), 3.27 (1H, d, *J* 11.7, 12'-*H*H), 3.11 (1H, m, 2'-H*H*), 3.02 (1H, m, 2'-*H*H), 2.72 (1H, d, *J* 13.6, 4-H*H*), 2.41 (2H, t, *J* 6.3, 6'-H_2), 2.34 (1H, m, 9-H*H*), 2.25 (1H, d, *J* 4.6, 5-OH), 2.22 (3H, s, 15-H_3), 2.15 (1H, m, 12-H), 2.12 (1H, m, 4-*H*H), 2.00 (1H, m, 9-*H*H), 1.95 (1H, m, 8-H), 1.46 (3H, s, 16'/17'-H_3), 1.43 (3H, s, 19'/20'-H_3), 1.41 (3H, s, 16'/17'-H_3), 1.34 (3H, s, 19'/20'-H_3), 1.03 (3H, s, 13'/14'-H_3), 1.02 (3H, s, 14-H_3), 0.98 (3H, d, *J* 6.2, 16-H_3), 0.96 (6H, m, 17-H_3 & 13'/14'-H_3), 0.88 (9H, s, $C(CH_3)_3$), 0.03 (6H, s, $Si(CH_3)_2$); δ_C (151 MHz, $CDCl_3$) 189.6 (C-1), 171.2 (C-5'), 170.3 (C-9'), 155.4 (C-3), 135.2 (C-11), 127.0 (C-10), 125.0 (d, *J* 62.8, C-2), 107.5 (C-18), 99.2 (C-15'), 82.3 (C-5, C-6 or C-7), 80.0 (C-5, C-6 or C-7), 77.4 (C-10'), 72.1 (C-13), 71.6 (C-12'), 67.9 (C-5, C-6 or C-7), 46.2 (d, *J* 6.9, C-4), 44.4 (C-12), 40.1 (C-3'), 37.1 (C-9), 36.3 (C-6'), 35.1 (C-7'), 33.1 (C-11'), 32.1 (C-8), 29.6 (C-16'/17'), 28.5 (C-2'), 28.0 (C-19/20), 26.0 ($SiC(CH_3)_3$), 25.6 (C-19/20), 22.3 (C-14), 20.7 (C-13'/14'), 20.3 (C-15'), 19.1 (C-13'/14'), 18.9 (C-16'/17'), 18.3 (Si*C*$(CH_3)_3$), 16.7 (C-16), 16.2 (C-17), -4.2 ($Si(CH_3)_2$), -4.7 ($Si(CH_3)_2$); IR (ν_{max}/cm^{-1}) ($CHCl_3$) 3312, 2962, 2931, 2859, 1649; HRMS (ESI/TOF) m/z: $[M + H]^+$ calculated for $^{13}CC_{39}H_{73}N_2O_9SSi$ 786.4840, found 786.6464.

[[1-^{13}C]-(2*E*,5*S*,6*S*,7*R*,8*R*,10*E*,12*R*,13*S*)-5,6,7,13-tetrahydroxy-3,8,12-trimethyltetradeca-2,10-dienoic acid] [pantetheine] (**2-Pant**):

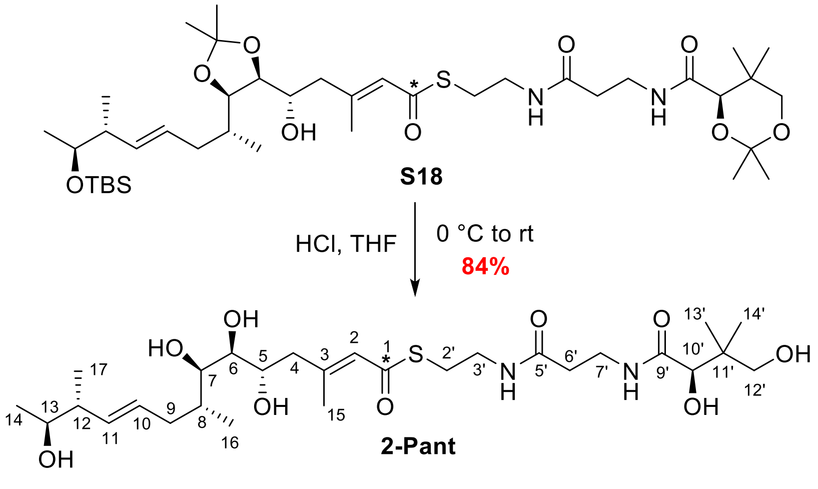

Diacetonide **S18** (40.0 mg, 0.051 mmol) was dissolved in THF (8 mL) and cooled to 0 °C under nitrogen. Aq. HCl (1 M, 2 mL) was added dropwise and the mixture was stirred and warmed to rt. After 24 h, TLC indicated the absence of starting material, but the presence of partially deprotected intermediates, therefore the mixture was re-cooled to 0 °C and further HCl (1 mL, 2 M) was added, the mixture stirred and allowed to warm to rt. After a further 24 h, TLC indicated product formation, but

the presence of partially protected material. The mixture was re-cooled to 0 °C, further HCl (2 mL, 2 M) was added, and the mixture was stirred and allowed to warm to rt. The mixture was quenched (54 h after the start of the reaction) by addition of sat. aq. $NaHCO_3$ (10 mL), EtOAc (10 mL) was added, and the two layers separated. The aqueous layer was extracted with EtOAc (5 x 10 mL), dried ($MgSO_4$), filtered, and concentrated *in vacuo*. The crude material was purified by column chromatography (10% MeOH:90% EtOAc to 15% MeOH:85% acetone with ~10 drops of AcOH/100 mL of eluent) to afford hexol **2-Pant** (25.1 mg, 0.042 mmol, 84%) as a clear oil.

$[\alpha]_D^{24}$ +6.0 (*c* 1.0, MeOH); δ_H (600 MHz, CD_3OD) 6.13 (1H, d, *J* 6.1, 2-H), 5.47 (1H, ddd, *J* 15.4, 7.6, 5.7, 10-H), 5.40 (1H, dd, *J* 15.4, 7.8, 11-H), 3.99 (1H, ddd, *J* 10.3, 4.9, 2.5, 5-H), 3.89 (1H, s, 10’-H), 3.62 (1H, m, 13-H), 3.56 (1H, dd, *J* 8.6, 4.9, 6-H), 3.50 (1H, m, 7’-H*H*), 3.48 – 3.44 (2H, m, 7’-*H*H & 12’-*H*H), 3.43 (1H, m, 7-H), 3.39 (1H, d, *J* 11.0, 12’-H*H*), 3.34 (1H, t, *J* 6.7, 3’-H_2), 3.05 – 3.00 (2H, m, 2’-H_2), 2.53 (1H, d, *J* 14.0, 4-H*H*), 2.41 (2H, t, *J* 6.7, 6’-H_2), 2.30 (1H, m, 9-H*H*), 2.23 (1H, dd, *J* 14.0, 10.3, 4-*H*H), 2.21 (3H, s, 15-H_3), 2.14 (1H, m, 12-H), 1.9 (1H, m, 8-H), 1.87 (1H, m, 9-*H*H), 1.10 (3H, d, *J* 6.4, 17-H_3), 0.99 (3H, d, *J* 6.9, 14-H_3), 0.98 (3H, d, *J* 6.8, 16-H_3), 0.92 (6H, s, 13’-H_3 and 14’-H_3); δ_C (151 MHz, CD_3OD) 190.0 (C-1), 176.1 (C-9’), 173.9 (C-5’), 157.3 (C-3), 134.6 (C-11), 131.3 (C-10), 125.4 (d, *J* 63.3, C-2), 78.3 (C-7), 77.3 (C-10’), 75.1 (C-6), 72.5 (C-5), 72.3 (C-13), 70.4 (C-12’), 45.3 (C-12), 43.8 (d, *J* 6.6, C-4), 40.4 (C-3’), 40.3 (C-11’), 36.43 (C-6’), 36.37 (C-7’), 36.2 (C-8), 34.3 (C-9), 29.0 (C-2’), 21.3 (C-13’/14’), 20.9 (C-13’/14’), 20.22 (C-17), 20.19 (C-15), 17.2 (C-16), 16.6 (C-14);

δ_H (700 MHz, CD_3OH)* 8.21 (1H, t, *J* 4.9 Hz, 8’-NH), 7.96 (1H, t, *J* 4.9 Hz, 4’-NH), 6.13 (1H, d, *J* 6.1, 2-H), 5.47 (1H, ddd, *J* 15.4, 7.6, 5.7, 10-H), 5.40 (1H, dd, *J* 15.4, 7.8, 11-H), 3.99 (1H, ddd, *J* 10.3, 4.9, 2.5, 5-H), 3.89 (1H, s, 10’-H), 3.62 (1H, m, 13-H), 3.56 (1H, dd, *J* 8.6, 4.9, 6-H), 3.50 (1H, m, 7’-H*H*), 3.48 – 3.44 (2H, m, 7’-*H*H & 12’-*H*H), 3.43 (1H, m, 7-H), 3.39 (1H, d, *J* 11.0, 12’-H*H*), 3.34 (1H, m, 3’-H_2), 3.05 – 3.00 (2H, m, 2’-H_2), 2.53 (1H, d, *J* 14.0, 4-H*H*), 2.41 (2H, t, *J* 6.7, 6’-H_2), 2.30 (1H, m, 9-H*H*), 2.23 (1H, dd, *J* 14.0, 10.3, 4-*H*H), 2.21 (3H, s, 15-H_3), 2.14 (1H, m, 12-H), 1.9 (1H, m, 8-H), 1.87 (1H, m, 9-*H*H), 1.10 (3H, d, *J* 6.4, 17-H_3), 0.99 (3H, d, *J* 6.9, 14-H_3), 0.98 (3H, d, *J* 6.8, 16-H_3), 0.92 (6H, s, 13’-H_3 and 14’-H_3)

*Underlined are differences to 600 MHz CD_3OD.

IR (ν_{max}/cm^{-1}) (MeOH) 3529, 3085, 2974, 2872, 1643; HRMS (ESI/TOF) m/z: $[M + Na]^+$ calculated for $^{13}CC_{27}H_{50}N_2O_9NaS$ 614.3169, found 614.3150.

<u>*O, O'* – Isopropylidene-D-pantothenate (**12**):</u>

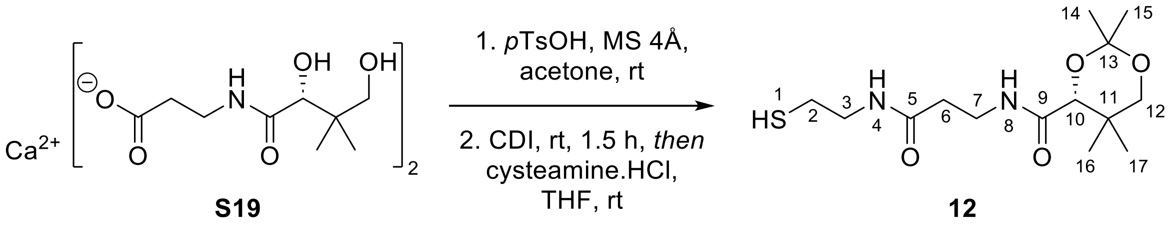

Calcium D-pantothenate **S19** (5.00 g, 21.0 mmol) was dissolved in acetone (250 mL) and *p*TsOH (4.79 g, 25.2 mmol) and molecular sieves (~5 g, 4 Å) were added and the mixture stirred at rt for 16 h under nitrogen. The reaction mixture formed a thick, white paste, to which EtOAc (50 mL) was added, and the mixture was filtered and washed with EtOAc (100 mL). The filtrate was concentrated *in vacuo* to a volume of ~30 mL. EtOAc (100 mL) and hexane (50 mL) were added and the mixture left to stand for 1 h. The solution was decanted and concentrated *in vacuo* to provide crude *O, O'* – isopropylidene-D-pantothenic acid as a white solid (4.15 g) which was used without further purification.

Crude *O, O'* – isopropylidene-D-pantothenic acid (4.150 g, 16.0 mmol) was dissolved in anhydrous THF (80 mL), and CDI (3.390 g, 21.9 mmol) was added, and the mixture stirred at rt for 30 min. Cysteamine hydrochloride (2.746 g, 24.2 mmol) was added and the mixture stirred for 16 h. The solvent was removed *in vacuo* and the resulting solid dissolved in DCM (200 mL). The solution was washed with sat. aq. NH_4Cl (2 x 100 mL), before being extracted with DCM (2 x 80 mL). The combined organic layers were washed with brine (100 mL), dried ($MgSO_4$), and filtered. The solvent was removed *in vacuo*, and the resultant oil was recrystallized (hexane/EtOAc) to afford *O, O'* – isopropylidene-D-pantothenate **12** (1.699 g, 5.3 mmol, 31% over two steps) as white crystals.

$[\alpha]_D^{23}$ +38 (*c* 1.00, $CHCl_3$), lit. $[\alpha]_D^{24}$ +48 (*c* 1.00, $CHCl_3$); δ_H (400 MHz, $CDCl_3$) 7.00 (1H, br m, 8-NH), 6.19 (1H, br m, 4-NH), 4.08 (1H, s, 10-H), 3.68 (1H, d, *J* 11.7, 12-H*H*), 3.65 – 3.50 (2H, m, 7-H_2), 3.50 – 3.34 (2H, m, 3-H_2), 3.28 (1H, d, *J* 11.7, 12-*H*H), 2.66 (2H, m, 2-H_2), 2.47 (2H, td, *J* 6.1, 1.7, 6-H_2), 1.46 (3H, s, 14-H_3 or 15-H_3), 1.42 (3H, s, 14-H_3 or 15-H_3), 1.36 (1H, t, *J* 8.5, SH), 1.04 (3H, s, 16-H_3 or 17-H_3), 0.97 (3H, s, 16-H_3 or 17-H_3); δ_C (101 MHz, $CDCl_3$) 171.2 (C-5), 170.4 (C-9), 99.2 (C-13), 77.3 (C-10), 71.5 (C-12), 42.5 (C-3), 36.2 (C-6), 34.9 (C-7), 33.1 (C-11), 29.6 (C-14/15), 24.6 (C-2), 22.2 (C-16/17), 19.0 (C-16/17), 18.8 (C-14/C15).

Data are consistent with literature values.[1]

3. Plasmid generation, protein expression and purification.

Reagents were purchased from Sigma-Aldrich, Thermo Fisher or Merck Millipore. E. coli competent cells were purchased from New England Biolabs (T7 Express and 5-α),Merck Millipore (Novagen BL21 (DE3) or Thermo Fisher Scientific (DH10β). All plasmids encoding the enzymes used in this study were purchased from Thermo Fisher Scientific.

The gene encoding MmpE_OR was amplified from *P. fluorescens* NCIMB 10856 genomic DNA with

OR_For:
5' ACGAATTCGAGCTCG TACGCCTTCGATTTTGCCC-3'
And OR_Rev:
5'- GGCCAGTGCCAAGCT TCATGTACCGGTTTGTCGCT-3'

primers and subcloned into the pET28a vector (pET28a-MmpE_OR), bearing a N-terminal His_6 tag and a thrombin cleavage site (LVPRGS). The nucleotide sequence for MmpAE, MmpA3a, MmpE_KS and MmpE_ACP were synthesized and sub-cloned into a pET151-D/TOPO vector bearing an N-terminal His_6 tag and a tobacco etch virus (TEV) cleavage site (ENLYFQ) by Thermo Fisher.

The pET151-MmpE_KS vector was used to generate MmpE_KS_M214A via the following mutagenic primers:

M214A_For
5'-AGCCTGGCACTGGATACCGCGTGTAGCAGC-3'
And M214A_Rev
5'-GCTGCTACACGCGGTATCCAGTGCCAGGCT-3'

Both MupN and CoaA/CoaD/CoaE were expressed and purified as described previously.[4]
Individual plasmids were transformed into E. coli T7 Express cells. Cultures were grown to OD600 = 0.7 in LB media (37 °C) supplemented with carbenicillin (100 µg/mL) (or 100 µg/mL kanamycin for pET28a-MmpE_OR) and induced (0.25 mM isopropyl β-D1-thiogalactopyranoside) at 16 °C (16 h) before cell pellets were harvested by centrifugation (6000 rpm, 10 mins) and resuspended in buffer A (50 mM Tris-HCl, 500 mM NaCl, 10% (v/v) glycerol, pH 8.0).

Harvested cells were sonicated and the soluble fraction was purified by immobilized metal affinity chromatography (IMAC) via a HiTrap 5 ml HP Ni column (GE Healthcare). Protein was eluted using a linear gradient from 6-100% of Buffer B (50 mM Tris-HCl, 500 mM NaCl, 10% (v/v) glycerol, 800

mM imidazole, pH 8.0). Eluted protein was further purified by size exclusion chromatography (SEC) using either a HiPrep 26/60 Sephacryl S100 or S200 column (GE Healthcare) in Buffer C (25 mM Tris-HCl, 150 mM NaCl, pH 7.5, 1 mM DTT) for MmpA, MmpA3a and MmpE_ACP or Buffer A (MmpE_KS, MmpE_KS_M214A and MmpE_OR) before protein concentration. Proteins were either immediately used or stored at -20 °C. Purified protein (50 µM) was analysed by analytical size exclusion chromatography using either a Superdex 75 10/300 or Superdex 200 increase 10/300 GL column (GE Healthcare) calibrated with molecular weight standards (GE Healthcare).[5]

For NMR studies ^{15}N labelled protein (MmpAE and MmpE_ACP) was produced from cells grown to OD_{600} = 2.0 in LB media supplemented with carbenicillin (100 µg/ml) at 37°C. Cells were then pelleted by centrifugation (6000 rpm, 10 mins), washed twice with sterile M9 media and then exchanged into M9 minimal media at a 4:1 volumetric ratio. Cells were supplemented with 1 gL-1 $^{15}NH_4Cl$, 0.5% (v/v) glycerol and 0.05% (w/v) glucose and induced with 0.25 mM IPTG, then harvested after 16 hrs at 16 °C. Cells were resuspended into buffer A and purified as described above for the unlabelled protein. Both MmpAE and MmpE_ACP were were cleaved overnight using in house TEV protease after IMAC prior to SEC.

4. Whole cell biotransformation with MmpE_OR

For in vivo biotransformations 50 ml of overnight culture of *E. coli* (BL21 DE3) cells overexpressing pET28a-MmpE_OR were harvested and resuspended in 2 ml 0.1 M K_2HPO4 pH 7.2 buffer supplemented with 20 mM glucose and 0.5 mg of Mupirocin W5, desepoxy PA-B, or PA-C, dissolved in MeOH. Cells were incubated at 30 °C, 180 rpm for 16 hrs. Reactions were quenched by adding equal volume acetonitrile, then vortexed and centrifuged. The acetonitrile layer was injected for LC-MS analysis.

5. *In-vitro* LCMS assays

5 mM of **2** dissolved in DMSO was incubated with 20 µM MmpE_OR, 1 mM FAD, 5 mM NADH in Buffer C for 1 h. Protein were then isolated by methanol chloroform precipitation and the resultant organic layer was injected for LC-MS analysis.

6. ESMS Assays

Samples were desalted for ESMS analysis using a C_4 ZipTip (Merck) per the manufacturer's instructions. Denatured samples were analysed on a Synapt G2-Si (Waters) fitted with a TriVersa NanoMate (Advion) using the following parameters: sample cone, 10 V; capillary voltage 1.5 kV; trap collision energy, 10 V. The source as set to positive mode and spectra were acquired over 200-3000 m/z and analysed using MassLynx 4.1 software. For Ppant ejection assays, an appropriate charge state

was isolated using the MSMS functionality. The transfer collision energy was increased until fragmentation was observed (typically 5 V to 20 V) and spectra were collected from 200-1000 m/z.

MmpE_KS ESMS translocation assay

100 μM MmpA3a was upgraded with pantetheine substrates: MBE pantetheine **(1-Pant)**, authentic substrate **2-Pant,** or propionyl CoA (100 μL reactions) as previously described[1] and desalted using a Zeba column (Thermo fisher) equilibrated with Buffer A.

Loaded ACP (100 μM) and MmpE_KS^0 or MmpE_KS^0_M214A (20 μM) were mixed in buffer A and incubated at room temperature for 1-3 h and analysed by MS (10 μl aliquot) at regular intervals. Interrogation of the acylated KS intermediates and Ppant ejection of ACP species were used to determine the outcome of the reaction.

To determine transfer to MmpE_ACP, 100 μM MmpE_ACP was converted to the holo- form using CoA (100 μL reaction) and desalted with a Zeba column (Thermo fisher) and equilibrated with buffer A. **2**-MmpA3a (50 μM) was incubated with holo-MmpE_ACP (50 μM) and MmpE_KS or MmpE_KS_M214A (20 μM) in buffer A and incubated at room temperature for 1-3 h and analysed by MS (10 μl aliquot) at regular intervals. Interrogation of the acylated KS intermediates and Ppant ejection of MmpE_ACP were used to determine the outcome of the reaction.

MmpE_OR ESMS assay

100 μM MmpE_ACP was upgraded with authentic substrate **2-Pant,** CoA, or the shorter mimic **15-Pant** (100 μL reactions) and desalted using a Zeba column (Thermo fisher) equilibrated with Buffer A. Loaded ACP (100 μM), FAD (1 mM), NADH (5 mM) MmpE_OR (20 μM) were mixed in buffer A and incubated at room temperature for 0-1 h and analysed by MS (10 μl aliquot) at regular intervals. Ppant ejection of ACP species were used to determine the outcome of the reaction. Addition of Fre (20 μM) and the additional substitution of NADH for NAD(P)H were used to determine the flavin specificity of MmpE_OR.

7. NMR Parameters

All NMR spectroscopy experiments were performed on a Bruker Avance III HD 700 MHz spectrometer (Bruker, Billerica, MA, USA) equipped with a 1.7 mm triple-resonance micro-cryoprobe using standard pulse sequences from the Bruker pulse library. For titrations of MmpA3a and MmpE_ACP with MmpE_KS^0, ^{1}H-^{15}N TROSY-HSQC spectra were recorded in (20 mM NaP, 0.5 M NaCl, 10% D_2O, pH8.0) at 288K.

The backbone of MmpAE and MmpE_ACP was assigned using standard 2D and 3D ^{15}N edited spectra (^{1}H-^{15}N TROSY-HSQC, ^{1}H-^{15}N HSQC-TOCSY and ^{1}H-^{15}N NOESY-HSQC (120ms mixing time)) acquired on a ^{15}N labelled sample at 298K. Spectra were referenced using 4,4-dimethyl-4-silapentane-1-sulfonic acid (DSS). All NMR data were processed in TopSpin 3.6.1 and analyzed using CCPNMR analysis v 2.4.2.[6]

8. Generation of the mmpE M214A point mutation.

Generation of mutation fragments. The mutation primers were designed as below. The codon ATG for methionine in the wild type sequence of KS9 was changed to CGC for alanine. The lower case sequences were homologous sequences from pEX18Tc vector. According to the primers designed the left hand and right-hand fragments amplified were 428 bp and 433 bp respectively. The two PCR products obtained were purified by column for cloning into the pEX18Tc vector.

mmpE-KS^{0}-M214A-FLF: 5'-acgaattcgagctcgGCATTACGCAAGTGCCGGCGAC-3'

mmpE-KS^{0}-M214A -FLR: 5'-CGAGGAACACGCGGTGTCGAGC-3'

mmpE-KS^{0}-M214A -FRF:5'- GCTCGACACCGCGTGTTCCTCG-3'

mmpE-KS^{0}-M214A -FRR: 5'-ggccagtgccaagctGGTTGCACCTGAGGCCGAGGTC-3'

Preparation of the E. coli donor strain. The vector pEX18Tc was linearised by double digestion with restriction enzymes *Kpn*1 and *Hind*III. The linearised vector pEX18Tc and the two mutation fragments were cloned together by homologous recombination (In-Fusion HD cloning kit, 5μl reaction). 2.5 μl reaction mixture was transformed into *E. coli* cloning strain DH10β to mini-prepare the mutation plasmid DNA pEX18Tc-KS9-M214A. The Plasmid DNA was then transformed into *E. coli* S17.1 (λ pir+) to obtain the conjugation donor strain.

Conjugation. A single *E. coli* S17.1-pEX18Tc-KS9-M214A colony was inoculated into a 3 ml of LB medium with 30 μg/ml tetracycline and incubate at 37°C overnight. 1.5 ml of the culture was taken, spun for 1 min and the cell pellet resuspended in 750 ml PBS (NaCl 8.0 g/L, KCl 0.2 g/L, $NaHPO_4$ 1.44/L, KH_2PO_4 0.24 g/L). The suspension was vortexed and spun for 1 min to obtain the *E. coli* doner cell pellet. At the same time, a single colony of *Pseudomonas fluorescence* NCIMB 10586 was inoculated into 3 ml of LB medium with 50 μg/ml ampicillin and incubate at 30°C overnight. 0.5 ml of culture was taken, spun for 1 min, resuspended in 750 ml PBS then vortexed and spin for a further 1 min. The cell pellet was resuspended in 1 ml LB medium (without antibiotic) and 100 μl of the cell suspension was added to the *E. coli* doner cell pellet prepared above. The cells were mixed by vortexing and spun for 1 min. The cell pellet mixture was resuspended in 20 μL LB medium and transfered onto an LB agar plate (without antibiotic). The agar plate was incubated at 30°C overnight.

The mating cells were collected in 1 ml PBS, vortexed and spun for 1 min. The cell pellet was resuspended in 500 µL LB medium then the cells spread on LB agar plates containing 50 µg/ml ampicillin and 30 µg/ml tetracycline. The plates were incubated at 30°C for 2-3 days for single colony selection.

Selection of 2nd recombination. Single colonies from above agar plates were inoculated into 3 ml LB medium without antibiotic and incubated at 30°C overnight to allow the 2nd recombination. The culture was then streaked onto No-Salt LB agar medium containing 20% sucrose (60 ml No-Salt LB and 40 ml 50% sucrose) for single colony selection. 3 to 5 colonies were picked for colony PCR using proper primer. The PCR products were sequenced for confirmation of correct mutation.

PA-A production. The KS9-M214A mutant strains together with the wild type strain (control) were incubated on LB agar plates and incubated at 30°C for 2 days to generate single colonies. A single colony was picked for each transformant and inoculated into 15 mL of LB medium in a 30 mL universal tube containing 30 µg/ml tetracycline. These seed cultures were incubated at 200 rpm, 25°C for 20 hours. The fermentation medium was based on LB medium. A glucose solution (40%) was autoclaved separately and added during inoculation to a concentration of 4% as the carbon source. The fermentation was performed in triplicate for each strain in 500 mL baffled flasks which were inoculated with 5 mL seed culture. The flasks were incubated at 200 rpm at 22°C for 50 hours.

Extraction. The whole cultures were acidified to pH4-5 with 2M HCl before extracted with ethyl acetate (1 v/v) twice. separated from the medium by centrifugation (8500 x g for 15 mins). The supernatant was extracted with ethyl acetate (1 v/v) once. The ethyl acetate phase from the two extractions was combined and dried over MgSO4 followed by evaporation *in vacuo*. The extract residue was resuspended in 2 ml MeOH for LC-MS analysis.

LC-MS analysis. The samples for LC-MS analysis were prepared by 10-fold dilution from the above samples. Waters LCMS system was equipped with a 2998 photodiode array detector, 2424 evaporative light scattering detector (ELSD) and Quattro Micro API mass spectrum. The analytical HPLC column (Phenomenex) used was Kinetex, 5µm, C18, 100A, 250 x 4.60 mm. The 20 min gradient HPLC program in water/acetonitrile (A/B) used was 0 min, 5%B; 1 min, 5%B; 2 min, 15%B; 15 min, 60%B; 16 min, 95%B; 17 min, 5%B; 20 min, 5%B. The flow rate was 1 mL/min. The PA-A production was calculated according to the target peak area via ELSD or UV peak area.

mmpE-KS^0-C215A was generated in an identical fashion to M214A with the following PCR primer pairs.

mmpE-KS^0-C215A-FLF: 5'-acgaattcgagctcg-GCATTACGCAAGTGCCGGCGAC-3'
mmpE-KS^0-C215A-FLR: 5'-CGCCGAGGAAGCCATGGTGTCG-3'
mmpE-KS^0-C215A-FRF: 5'-CGACACCATGGCTTCCTCGGCG-3'
mmpE-KS^0-C215A-FRR: 5'-ggccagtgccaagct-GGTTGCACCTGAGGCCGAGGTC-3'

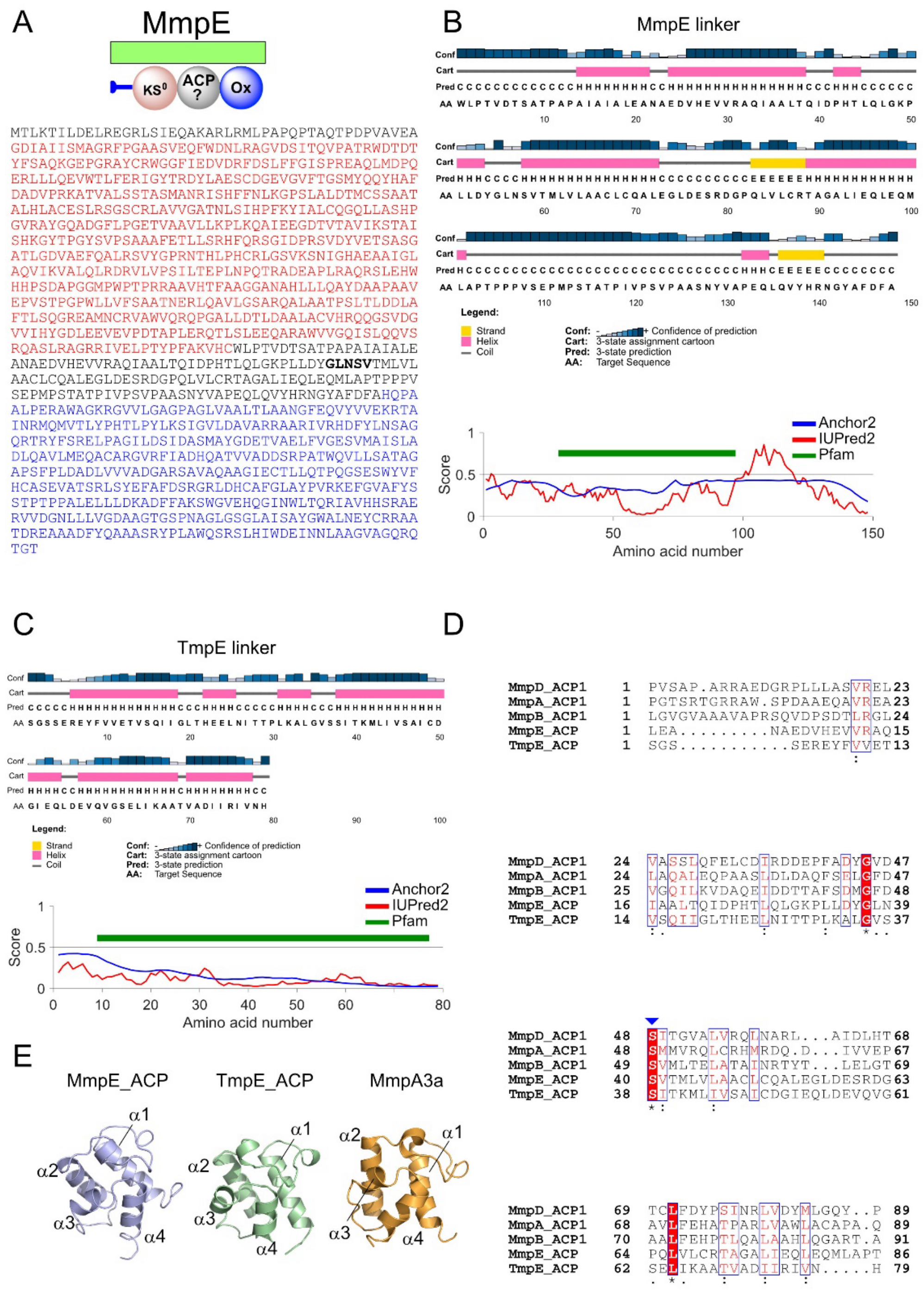

Figure S1. Identification of a ACP within the MmpE module. *A) Sequence of MmpE with KS^{0} and OR highlighted in red and blue respectively. The presence of a ACP motif (GLNSF) is highlighted. B) PSIPRED profile of the linker between MmpE_KS^{0} and the OR domain. IUPRED2 analysis indicated the contiguous 4 α-helix sequence within this linker region was not disordered. C) PSIPRED and IUPRED2 profiles of an equivalent linker in TmpE between the annotated TmpE_KS^{0} and TmpE_OR domains, indicating this 4 α-helix sequence is conserved. D) Sequence alignment of the putative ACP sequences with MmpE and TmpE with characterised ACPs within MmpD and MmpA (MmpD_ACP1 and MmpA_ACP1). E) Ab initio homology models of putative MmpE_ACP and TmpE_ACP sequences, compared to the structure of MmpA3a indicating the structural arrangement of a 4 helix bundle.*[4b, 7]

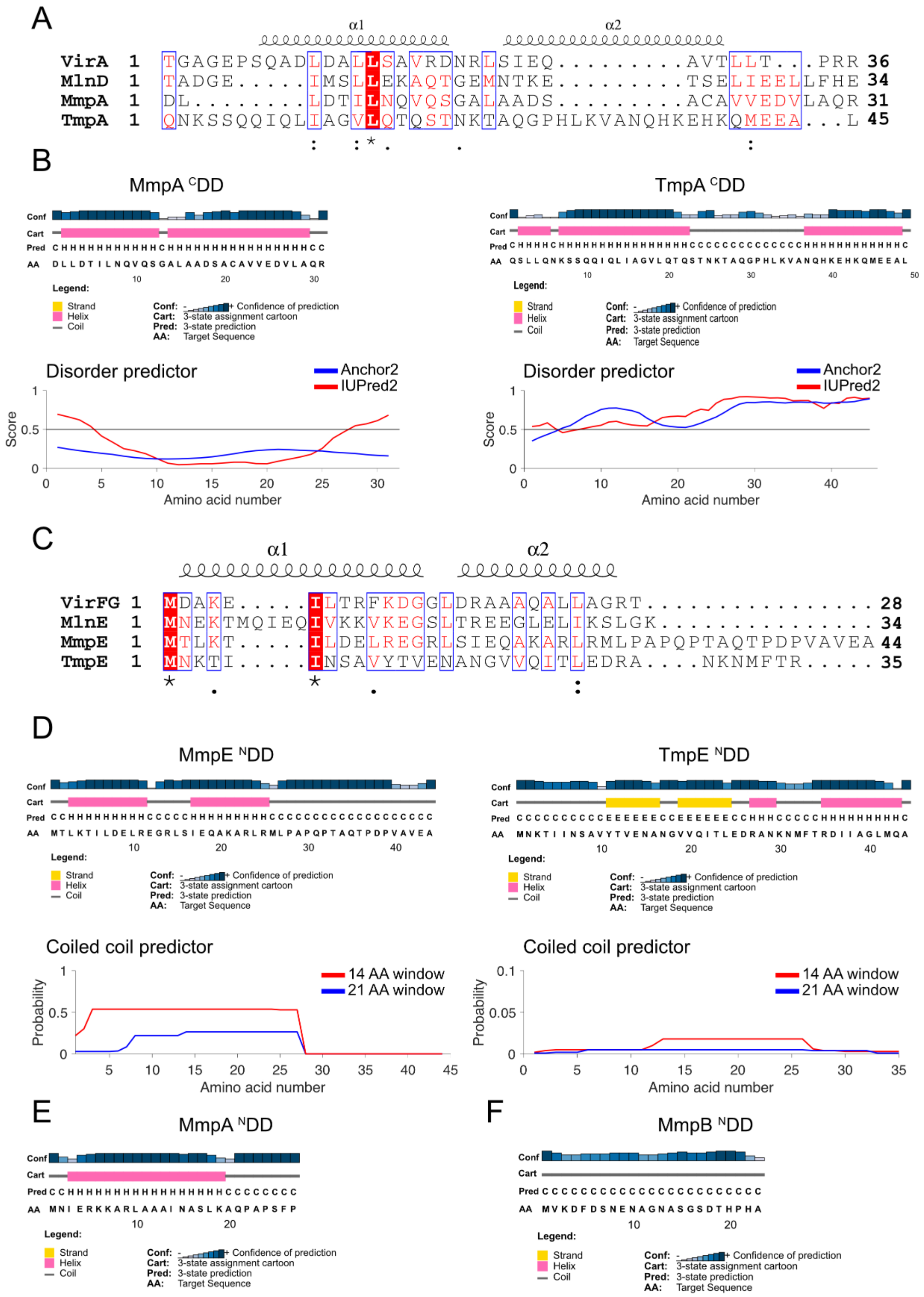
A
α1
α2
VirA 1 TGAGEPSQADLDALLSAVRDNRLSIEQ.........AVTLLT...PRR 36
MlnD 1 TADGE.....IMSLLEKAQTGEMNTKE.........TSELIEELLFHE 34
MmpA 1 DL........LDTILNQVQSGALAADS.........ACAVVEDVLAQR 31
TmpA 1 QNKSSQQIQLIAGVLQTQSTNKTAQGPHLKVANQHKEHKQMEEA...L 45
B
MmpA CDD
Conf
Cart
Pred CHHHHHHHHHHCHHHHHHHHHHHHHHHHHCC
AA DLLDTILNQVQSGALAADSACAVVEDVLAQR
Legend:
Strand
Helix
Coil
Conf: - + Confidence of prediction
Cart: 3-state assignment cartoon
Pred: 3-state prediction
AA: Target Sequence
TmpA CDD
Pred CHHHHCHHHHHHHHHHHHHHHHCCCCCCCCCCCCCCHHHHHHHHHHHHC
AA QSLLQNKSSQQIQLIAGVLQTQSTNKTAQGPHLKVANQHKEHKQMEEAL
Disorder predictor
Anchor2
IUPred2
Score
Amino acid number
C
α1
α2
VirFG 1 MDAKE.....ILTRFKDGGLDRAAAQALLAGRT................ 28
MlnE 1 MNEKTMQIEQIVKKVKEGSLTREEGLELIKSLGK.............. 34
MmpE 1 MTLKT.....ILDELREGRLSIEQAKARLRMLPAPQPTAQTPDPVAVEA 44
TmpE 1 MNKTI.....INSAVYTVENANGVVQITLEDRA....NKNMFTR..... 35
D
MmpE NDD
Pred CCHHHHHHHHHCCCCHHHHHHHHHCCCCCCCCCCCCCCCCC
AA MTLKTILDELREGRLSIEQAKARLRMLPAPQPTAQTPDPVAVEA
TmpE NDD
Pred CCCCCCCCCEEEEEECCEEEEEECCHHHCCCCCHHHHHHHHHC
AA MNKTIINSAVYTVENANGVVQITLEDRANKNMFTRDIIAGLMQA
Coiled coil predictor
14 AA window
21 AA window
Probability
Amino acid number
E
MmpA NDD
Pred CCHHHHHHHHHHHHHHHHHHCCCCCCCC
AA MNIERKKARLAAAINASLKAQPAPSFP
F
MmpB NDD
Pred CCCCCCCCCCCCCCCCCCCCCCCCC
AA MVKDFDSNENAGNASGSDTHPHA

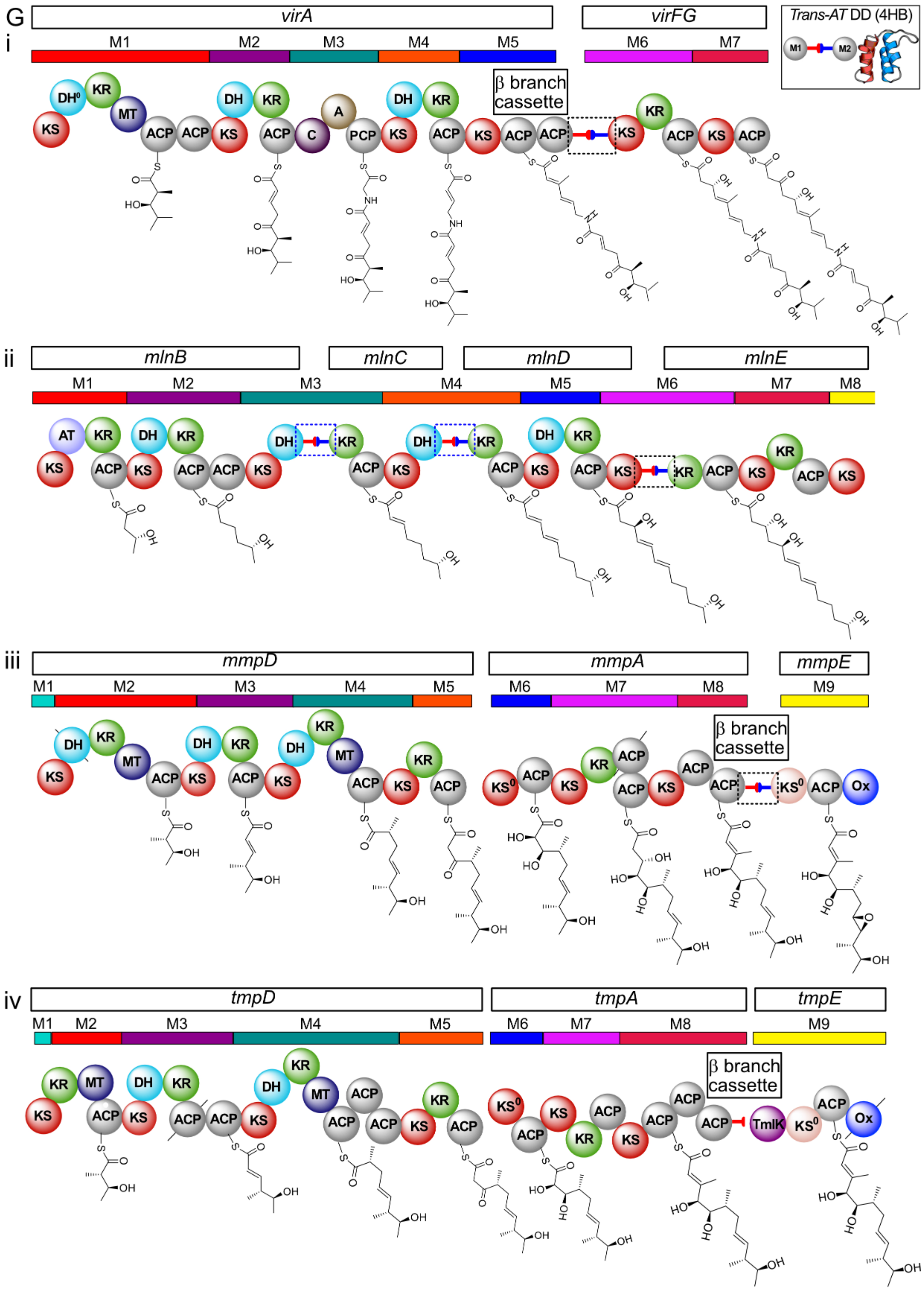

G
i
virA
virFG
Trans-AT DD (4HB)
M1
M2
M3
M4
M5
M6
M7
β branch cassette
ii
mlnB
mlnC
mlnD
mlnE
M8
iii
mmpD
mmpA
mmpE
M9
iv
tmpD
tmpA
tmpE

***Figure S2**. **Bioinformatic analysis of candidate ^{C}DD and ^{N}DD docking domain regions with the Mupirocin and Thiomarinol gene clusters**. A) Sequence alignment of the C-terminal residues downstream of MmpA3b and TmpA3c with experimentally determined ^{C}DD sequences from the virginamycin biosynthetic pathway within Streptomyces virginiae (VirA) and the Macrolactin biosynthetic pathway (MlnD) within Bacillus amyloliquefaciens. The α-helices encoded within the VirA ^{C}DD are highlighted. B) Secondary structure prediction via PSIPRED and intrinsic disorder/interaction propensity via IUPRED2/Anchor2 was predicted for both candidate ^{C}DD within MmpA and TmpA.[8] C) Sequence alignment of the N-terminal residues upstream of MmpE_KS and TmlK with experimentally determine ^{N}DD sequences from VirFG and MlnE. The α-helices encoded within the VirFG N_{DD} are highlighted. D) Secondary structure prediction via PSIPRED and intrinsic disorder/interaction propensity via IUPRED2/Anchor2 was predicted for both candidate ^{N}DD within MmpE and TmpE. E) PSIPRED profile of MmpA_^{N}DD which displayed a 27 amino acid unannotated N-terminus. F) PSIPRED profile of MmpB_^{N}DD which displayed a 23 amino acid unannotated N-terminus. G) 4HB docking domains within Trans-AT PKS – (i) 4HB across VirA-VirFG modules between (ACP-KS) immediately after the β-branch cassette on VirA-5a/b. ii) 4HB across MlnD-MlnE modules between (KS-KR) in black. Putative additional 4HB domains are highlighted in blue. iii) 4HB across MmpA and MmpE (ACP-KS) immediately after the β-branch cassette on MmpA_ACP3a/3b. iv) Lack of 4HB across TmpA and TmpE due to the presence of TmlK fused to the N-terminus of TmpE.*

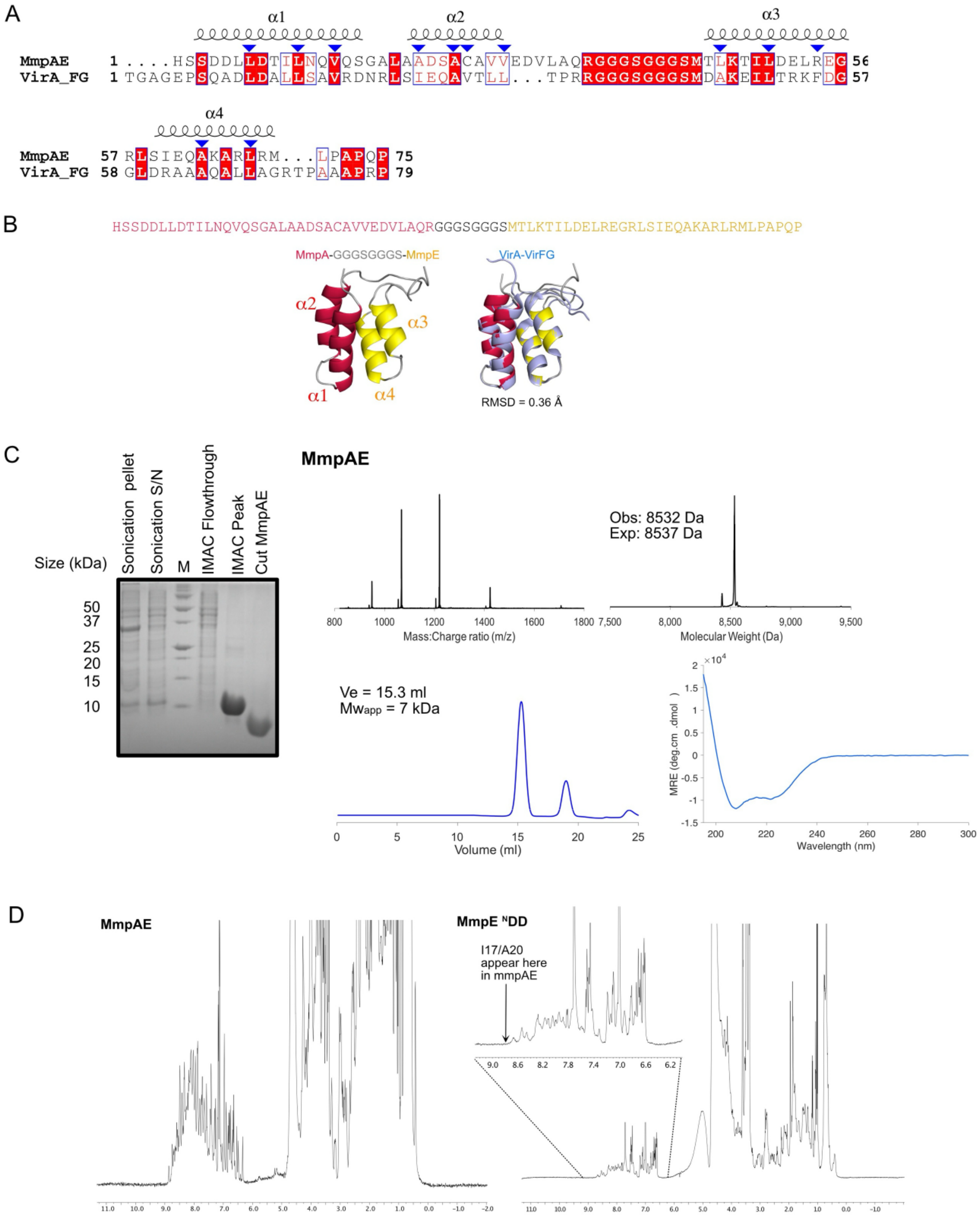

Figure S3. Characterisation of a docking domain between MmpA and MmpE. *A) Sequence alignment between a candidate MmpA_MmpE (MmpAE) docking domain and the experimentally characterised VirA-FG docking domain. The α-helices encoded within the VirA-FG DD are highlighted. Blue arrows indicate residues critical for the hydrophobic interface within the four helix bundle of VirA-FG. B) Sequence of MmpAE with regions corresponding to the MmpA_^{C}DD, glycine rich linker and MmpE_^{N}DD highlighted in red, grey and yellow respectively. Ab initio homology modelling of MmpAE predicted a high structural similarity with VirA-FG (RMSD = 0.36 Å). C) Protein purification and characterisation of MmpAE. SDS-PAGE (cut indicates removal of the His$_6$ tag), analytical SEC showing cut MmpAE eluting as a monomer, ESMS confirming the expected mass of cleaved uniformly ^{15}N labelled MmpAE and CD indicated this construct displayed an α-helical profile. D) 1D NMR spectra of unlabelled MmpAE (left) and MmpE ^{N}DD (right). Well-dispersed signals I17 and A20 (See Figure 3) in MmpAE are not present in the spectrum of just the N-terminal docking domain due to lack of structuring.*

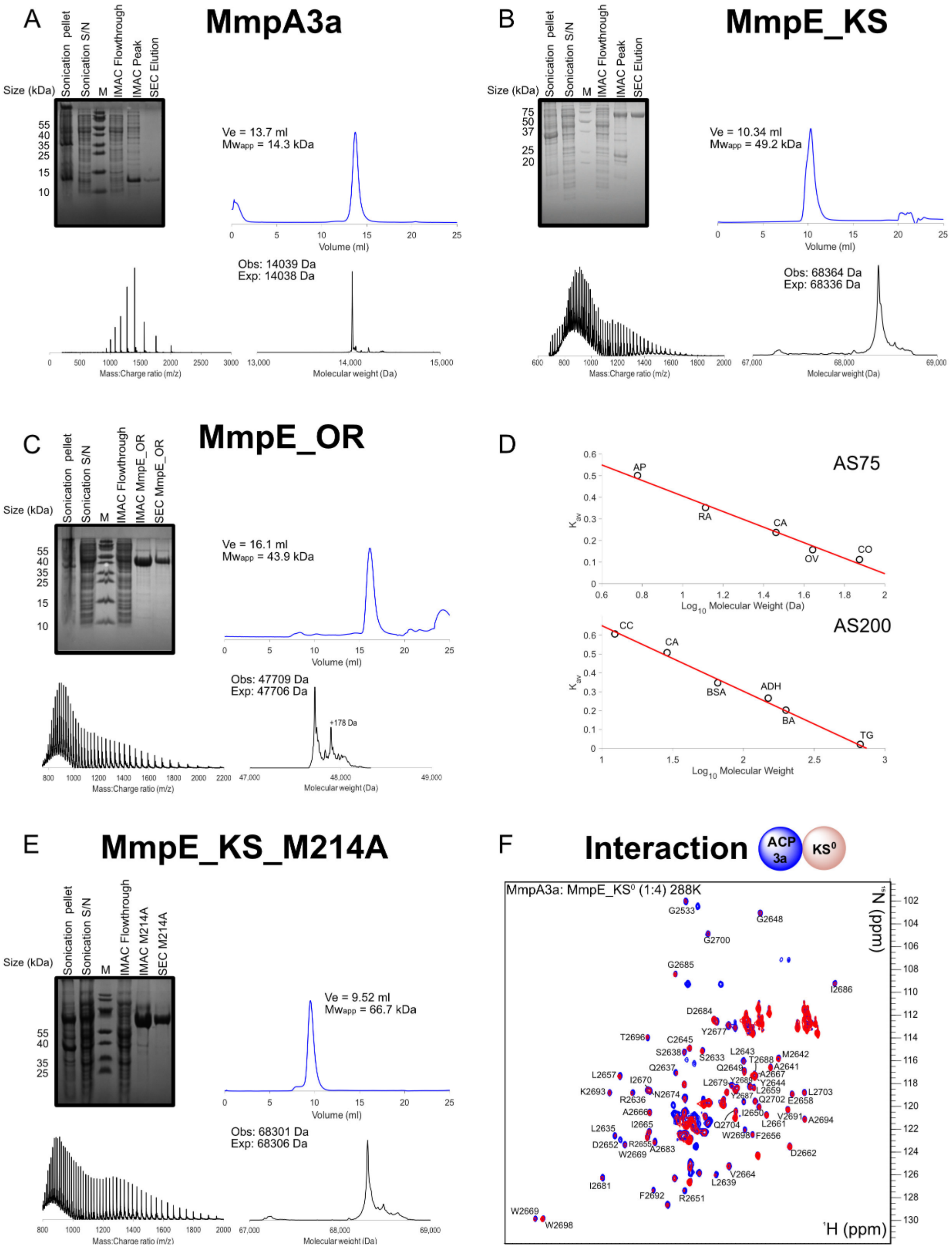

A
MmpA3a
Size (kDa)
Sonication pellet
Sonication S/N
M
IMAC Flowthrough
IMAC Peak
SEC Elution
Ve = 13.7 ml
Mwapp = 14.3 kDa
Volume (ml)
Obs: 14039 Da
Exp: 14038 Da
Mass:Charge ratio (m/z)
Molecular weight (Da)
B
MmpE_KS
Ve = 10.34 ml
Mwapp = 49.2 kDa
Obs: 68364 Da
Exp: 68336 Da
C
MmpE_OR
IMAC MmpE_OR
SEC MmpE_OR
Ve = 16.1 ml
Mwapp = 43.9 kDa
Obs: 47709 Da
Exp: 47706 Da
+178 Da
D
AS75
AP
RA
CA
OV
CO
Log10 Molecular Weight (Da)
AS200
CC
BSA
ADH
BA
TG
Log10 Molecular Weight
E
MmpE_KS_M214A
IMAC M214A
SEC M214A
Ve = 9.52 ml
Mwapp = 66.7 kDa
Obs: 68301 Da
Exp: 68306 Da
F
Interaction
ACP 3a
KS0
MmpA3a: MmpE_KS0 (1:4) 288K
15N (ppm)
1H (ppm)

Figure S4. Protein purification and characterisation. *A)* ***MmpA3a****. SDS-PAGE following the purification after sonication, IMAC and SEC. Analytical SEC (AS75) showing MmpA3a.6His eluting as a monomer and non-native ESMS confirming the correct sequence of MmpA3a. B)* ***MmpE_KS****. As A) SDS-PAGE following the purification after sonication, IMAC and SEC, with analytical SEC (AS75) confirming a monomeric species of MmpE_KS and non-native ESMS confirming the correct sequence. C)* ***MmpE_OR****. As B). SDS-PAGE following the purification after sonication, IMAC and SEC. Analytical SEC (AS200) showing MmpE_OR eluting as a monomer and non-native ESMS confirming the correct sequence. D) Calibration curve for the Analytical S75 and Analytical S200 column using the following calibrants: Aprotinin (AP), Ribonuclease A (RA), Carbonic Anhydrase (CA), Ovalbumin (OV), Conalbumin (CO), Cytochrome C (CC), Bovine Serum Albumin (BSA), Alcohol Dehydrogenase (ADH), β-Amylase (BA) and Thyroglobulin (TG). E)* ***MmpE_KS_M214A****, As A) SDS-PAGE following the purification after sonication, IMAC and SEC, with analytical SEC (AS75) confirming a monomeric species of MmpE_KS_M214A and non-native ESMS confirming the correct sequence. F) ^{1}H-^{15}N HSQC NMR spectra of ^{15}N labelled MmpA3a ('ACP3a') before (blue correlations) and after (red correlations) addition of MmpE_KS^{0}.*

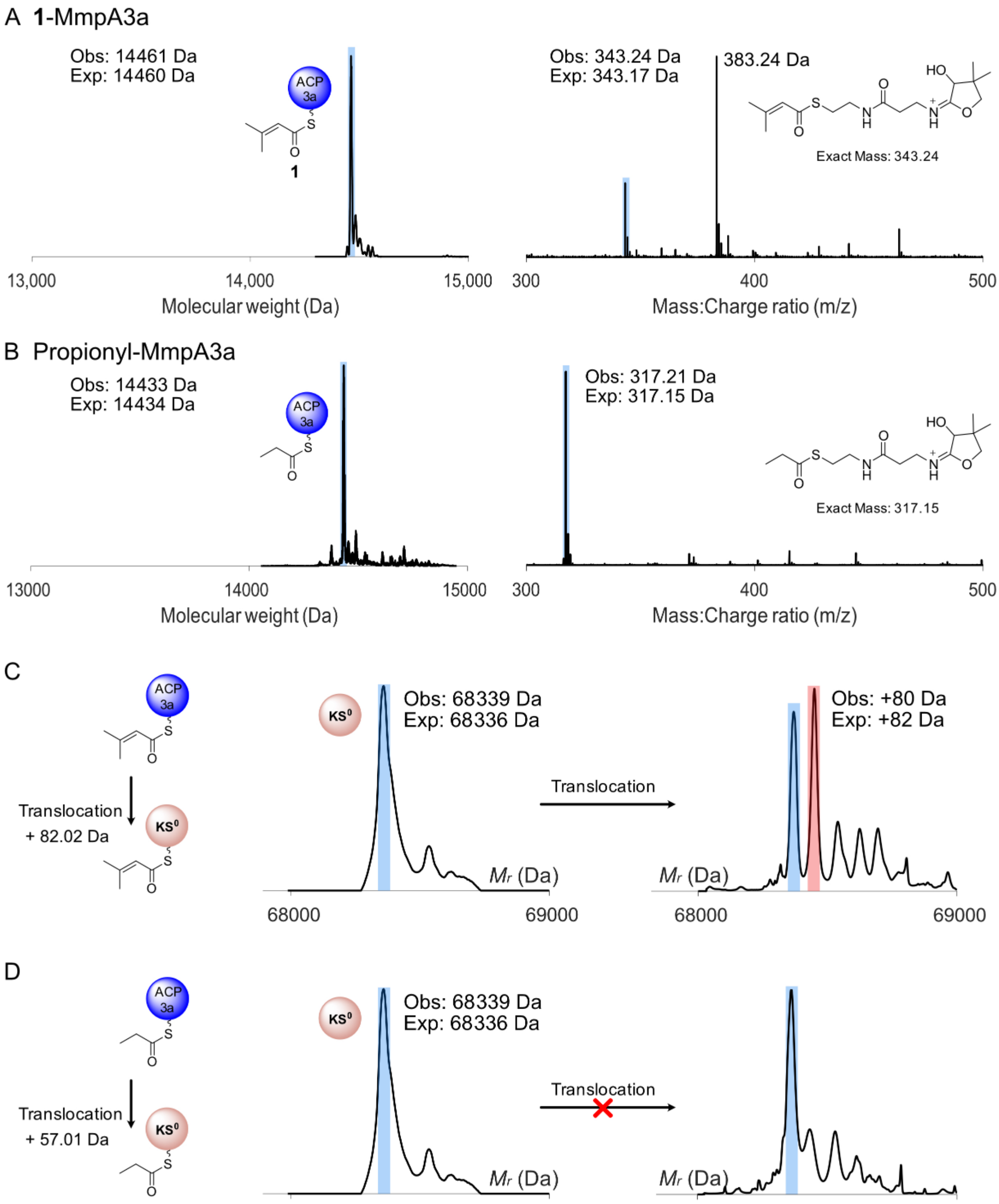

Figure S5. Translocation of 1 and propionyl between MmpA3a and MmpE_KS. *A) Deconvoluted spectrum of **1**-MmpA3a (obs: 14,461 Da, exp: 14,460 Da) and the Ppant ejection for this species (obs: 343.24 Da, exp: 343.17 Da). B) Deconvoluted spectrum of Prop-MmpA3a (obs: 14,433 Da, exp: 14,434 Da) and the Ppant ejection for this species (obs: 317.21 Da, exp: 317.15 Da) C) Translocation of **1** from MmpA3a to MmpE_KS - Deconvolution spectra of apo-MmpE_KS (left (Obs: 68,339 Da, exp: 68,336 Da)) and when incubated with **1**-MmpA3a (right): MmpE_KS (note assay conditions in salt reduced mass accuracy: Obs 68369 Da, exp 68336 Da) and **1**-MmpE_KS (Obs: 68,449 Da (+80 Da), Exp: 68,418 Da (+82 Da)). D) Translocation of propionyl from MmpA3a to MmpE_KS - Deconvolution spectra of apo-MmpE_KS (left) and when incubated with Prop-MmpA3a (right): MmpE_KS (Obs: 68,339 Da, exp: 68,336 Da) and Prop-MmpE_KS (Exp: 68,393 Da, this was not observed).*

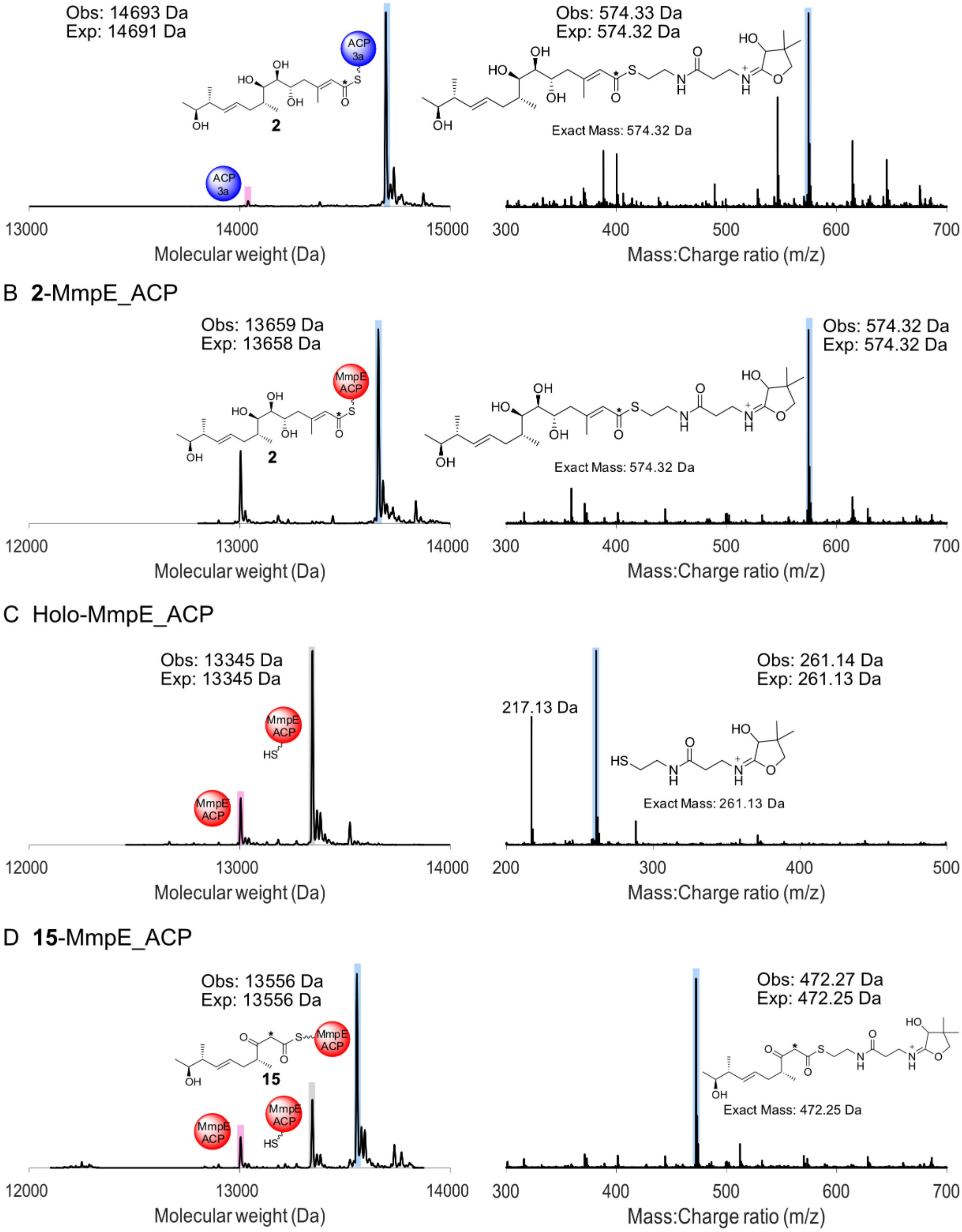

***Figure S6**. **MmpE_ACP upgrades for epoxidation assays** A) Deconvoluted spectrum of **2**-MmpA3a (obs: 14,693 Da, exp: 14,691 Da) and the Ppant ejection for this species (obs: 574.33 Da, exp: 574.32 Da). B) Deconvoluted spectrum of **2**-MmpE_ACP (obs: 13,659 Da, exp: 13,658 Da) and the Ppant ejection for this species (obs: 574.32 Da, exp: 574.32 Da). C) Deconvoluted spectrum of holo-MmpE_ACP (obs: 13,345 Da, exp: 13,345 Da) and the Ppant ejection for this species (obs: 261.14 Da, exp: 261.13 Da). D) Deconvoluted spectrum of **15**-MmpE_ACP (obs: 13,556 Da, exp: 13,556 Da) and the Ppant ejection for this species (obs: 472.27 Da, exp: 472.25 Da). * = ^{13}C label.*

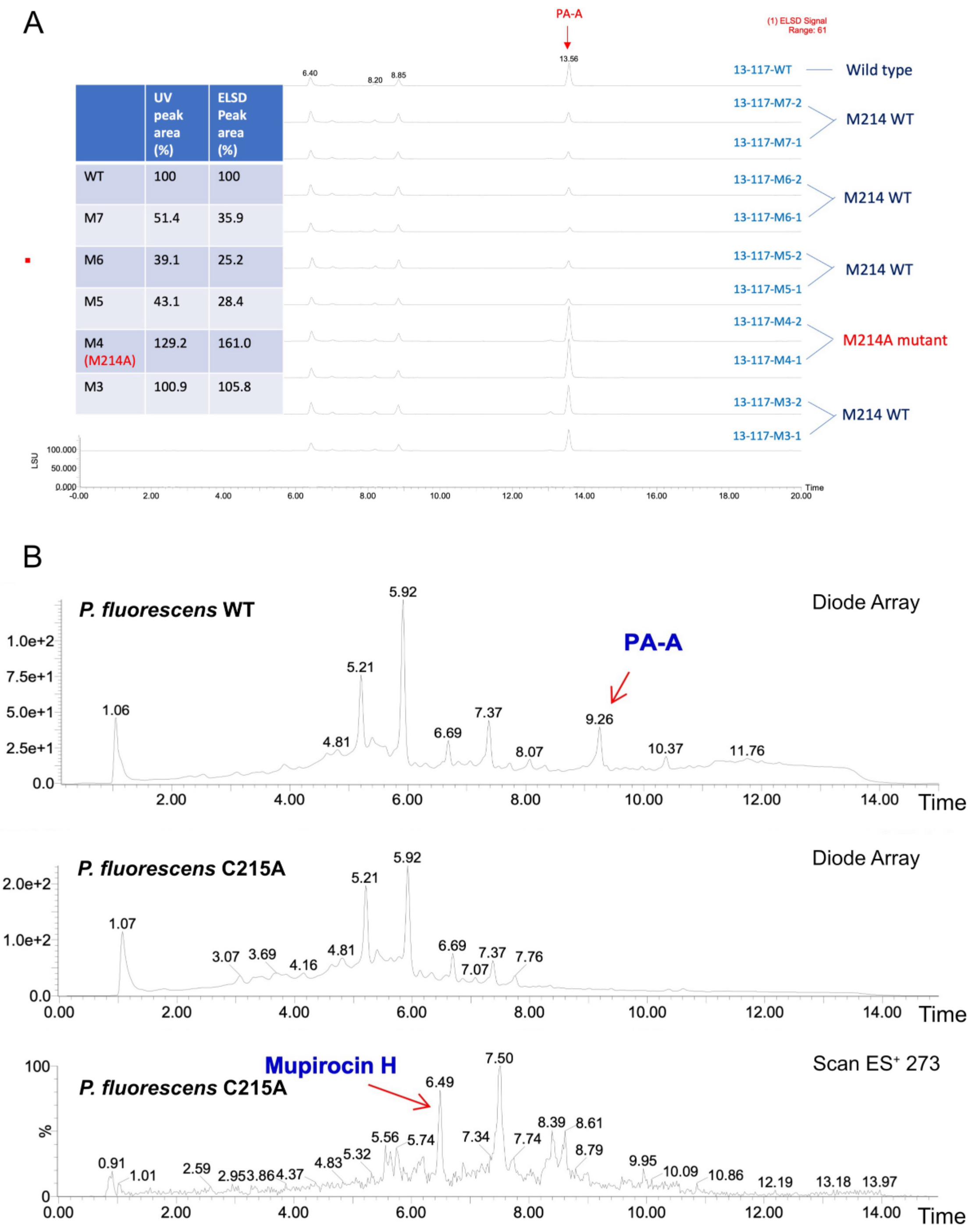

Figure S7*. *Fermentation of M214A and C215A mutants. A) PA-A production by *P. fluorescens* WT, off-target mutants and M214A. WT: wild type strain of *P. fluorescens*, M214A mutant confirmed by sequencing. 2 transformant colonies were picked and examined (transformant M4-1 and M4-2). M214WT: false M214A transformants confirmed by sequencing. The mutation occurred off target (not M214A). These include M3-1, M3-2, M5-1, M5-2, M6-1, M6-2, M7-1 and M7-2). B Metabolite analysis for WT and C215A. Mupirocin H was detected in the C215A fermentation and no PA-A. Trace for mupirocin H ES$^+$ 273 is shown. (also in ES$^+$: 255 [M-H2O+H]$^+$; 273 [M+H]$^+$; 295 [M+Na]$^+$.

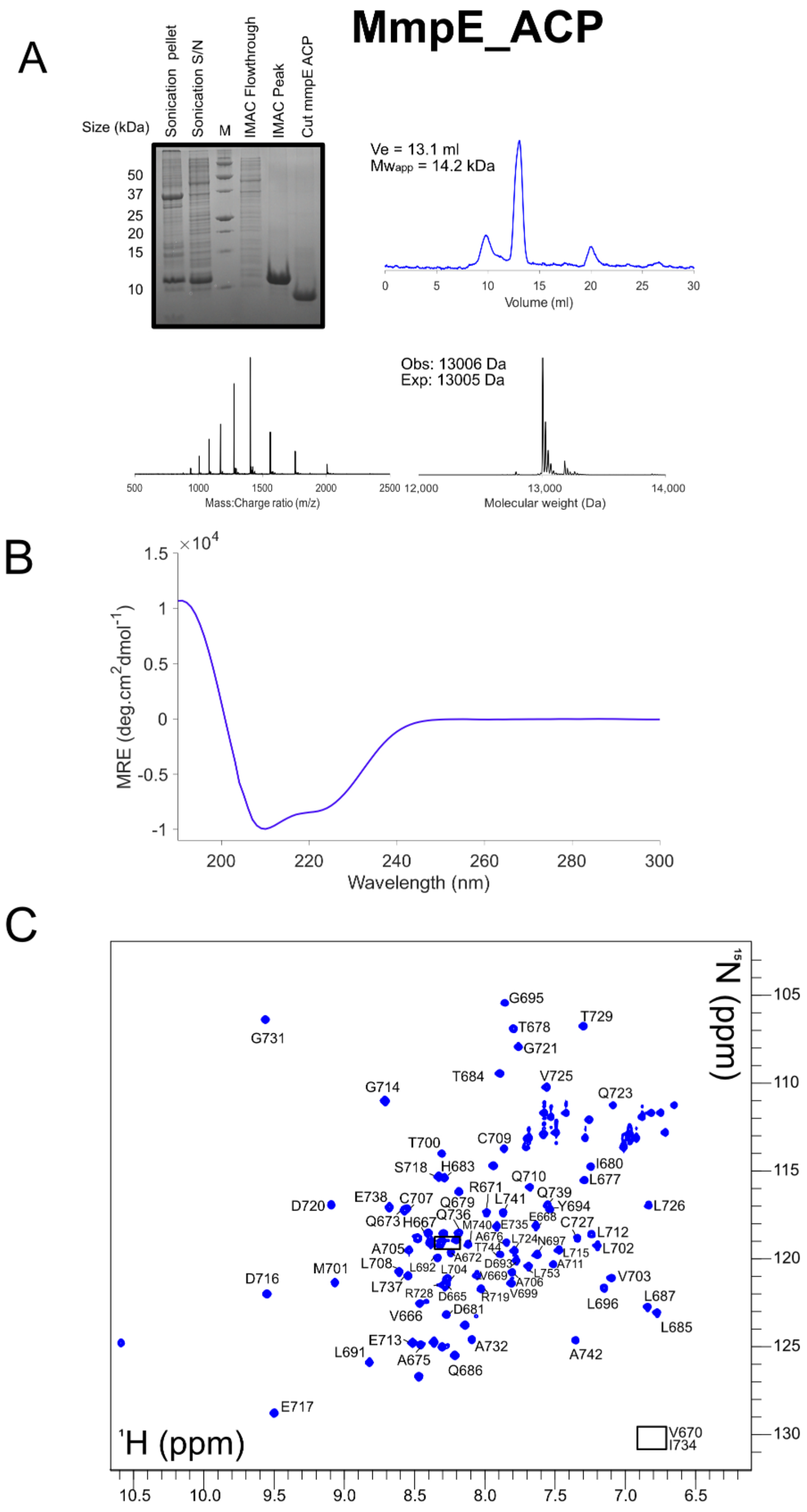

Figure S8: Characterisation of MmpE_ACP. *A) SDS-PAGE following the purification after sonication, IMAC, before cleavage of the His6 tag and SEC post cleavage. Analytical SEC showing MmpE_ACP eluting as a monomeric species and non-native ESMS confirming the correct sequence of MmpE_ACP. B) CD spectra of MmpE_ACP displaying an α-helical profile. C) ^{1}H-^{15}N HSQC spectra of cut ^{15}N-apo-MmpE_ACP with assignment numbering set to the amino acid sequence of full-length MmpE.*

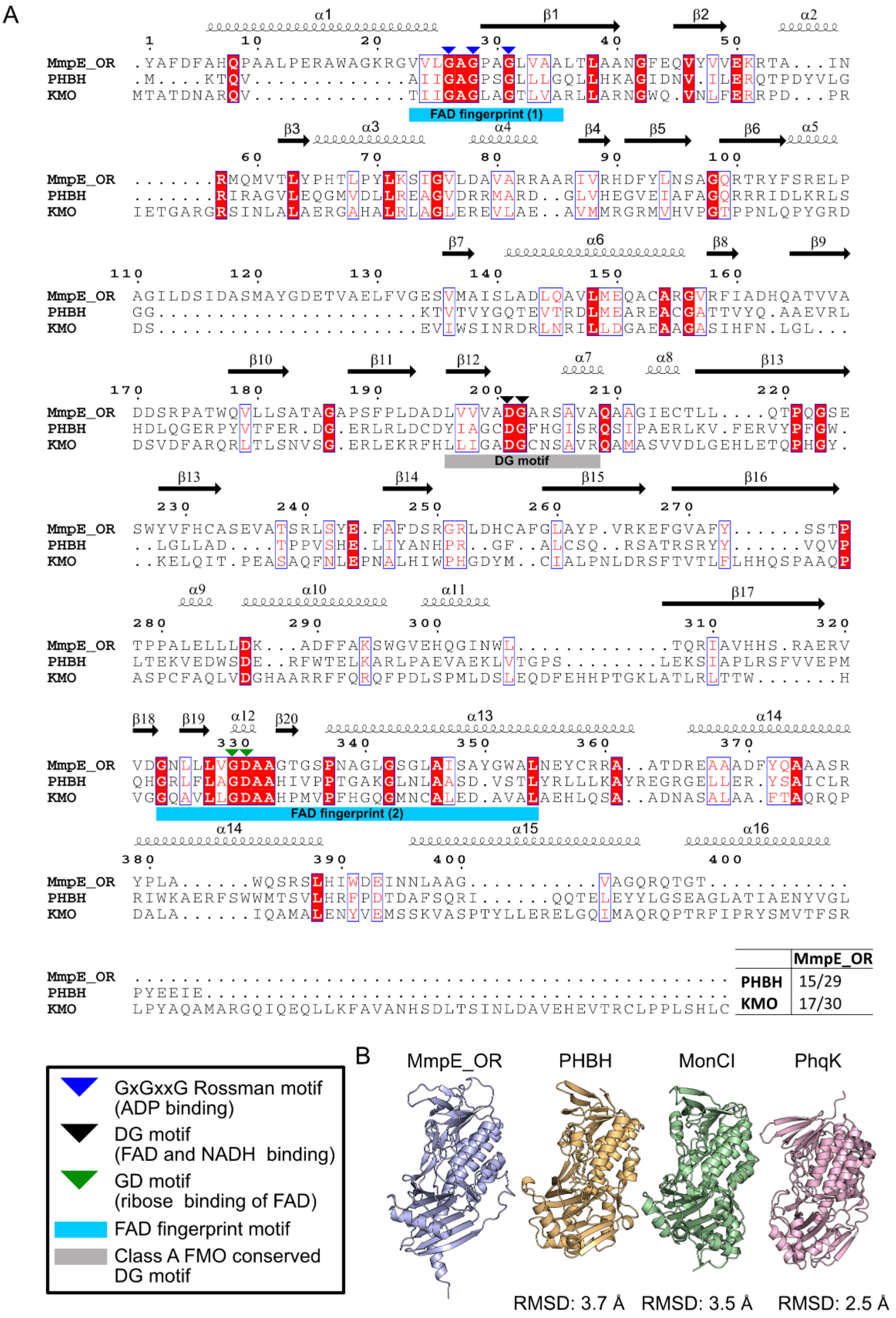

Figure S9. Multiple sequence alignment of MmpE_OR (uniprot: Q8RL60) with Class A Flavin dependent monooxygenases. *A) Sequences from* Pseudomonas fluorescens (*p*-hydroxybenzoate hydroxylase (PHBH), uniprot: P00438) and kynureine 3-monooxygenase (KMO, uniprot: Q84HF5). (Inset): Sequence identity/similarity (%) of MmpE_OR to PHBH and KMO. The FAD fingerprint motifs highlighting the rossman fold (blue arrow) and GD motif (green arrow) that are responsible for ADP and ribose binding of FAD are highlighted. The DG motif observed in class A FMOs that interacts with FAD and NADH is also highlighted (black arrow).[9] B) Structural comparison of an *ab initio* homology model of MmpE_OR (light blue) with the crystal structure of PHBH (light orange, PDB: 1PBE, RMSD: 3.7 Å), MonCI (green, PDB:8T3P) and PhqK (pink, PDB:6PVG) indicating the presence of the PHBH fold.

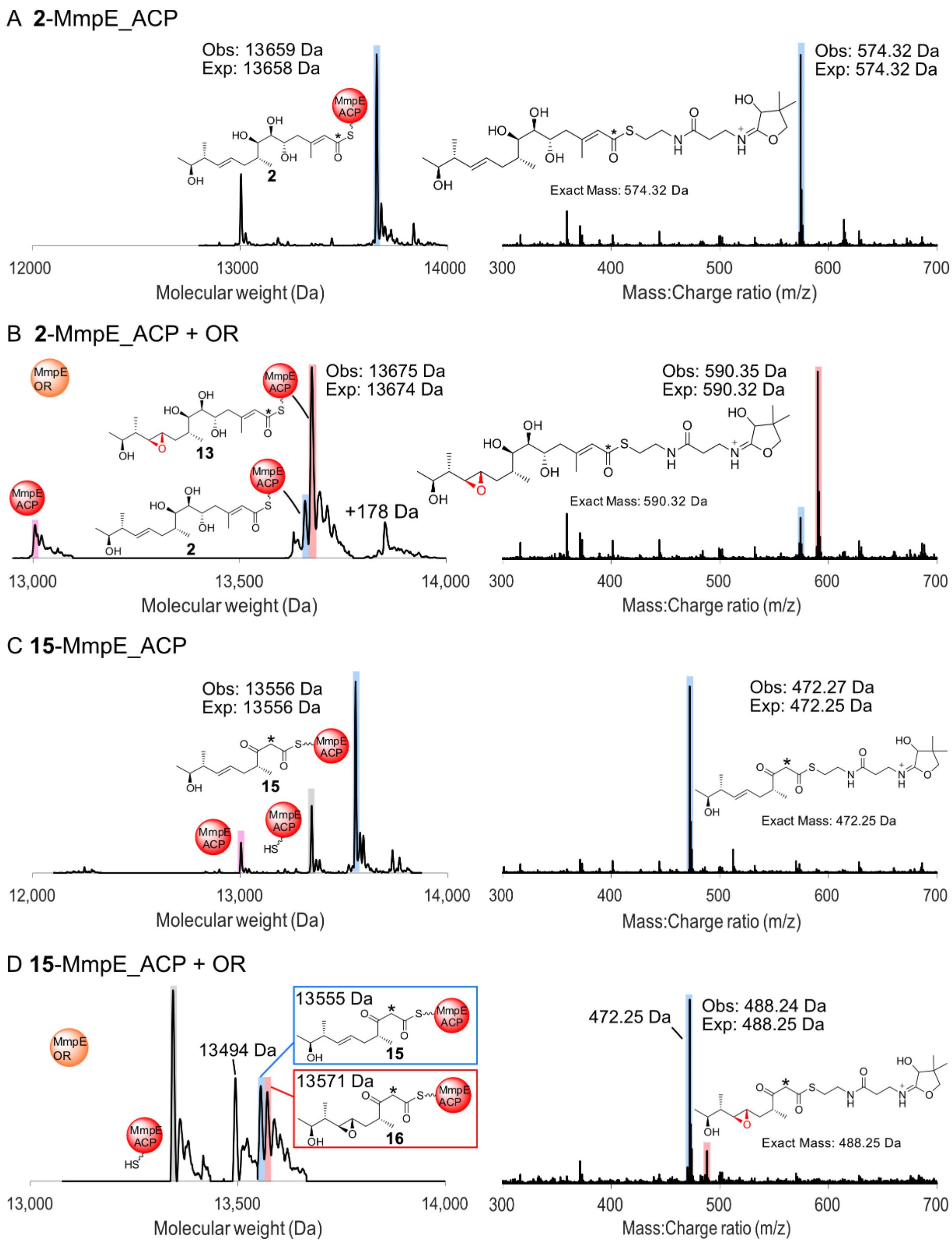

Figure S10. Epoxidation assays utilising MmpE_OR. *A) Deconvoluted spectra of **2**-MmpE_ACP in the absence of MmpE_OR (Obs: 13,659 Da, Exp: 13,658 Da). (right): Ppant ejection for this species (obs: 574.32 Da, exp: 574.32 Da). B) Deconvoluted spectra of the epoxidation reaction when **2**-MmpE_ACP was incubated with MmpE_OR to give **13**-MmpE_ACP (Obs:13,675 Da, Exp: 13,674 Da). (right): Ppant ejection ion (Obs 590.35 Da, Exp: 590.32 Da). C) Deconvoluted spectra of **15**-MmpE_ACP in the absence of MmpE_OR (Obs: 13,556 Da, Exp: 13,556 Da). (right): Ppant ejection ion of the **15**-MmpE_ACP species (Obs: 472.27 Da, Exp: 472.25 Da). D) Deconvoluted spectra of **15**-MmpE_ACP when incubated with MmpE_OR (Obs: 13,344 Da (Holo-ACP), 13,555 Da (**15**-MmpE_ACP) and 13,571 Da (**16**-MmpE_ACP), Exp: 13,572 Da). The signal at 13494 was not identified. (right): Ppant ejection ion of **16**-MmpE_ACP. (Obs: 488.24 Da, Exp: 488.25 Da). Species are highlighted as apo (pink), holo (grey), alkene (**2** or **15)** (blue), epoxide (**13** or **16)** (red) for MmpE_ACP. * = ^{13}C label.*

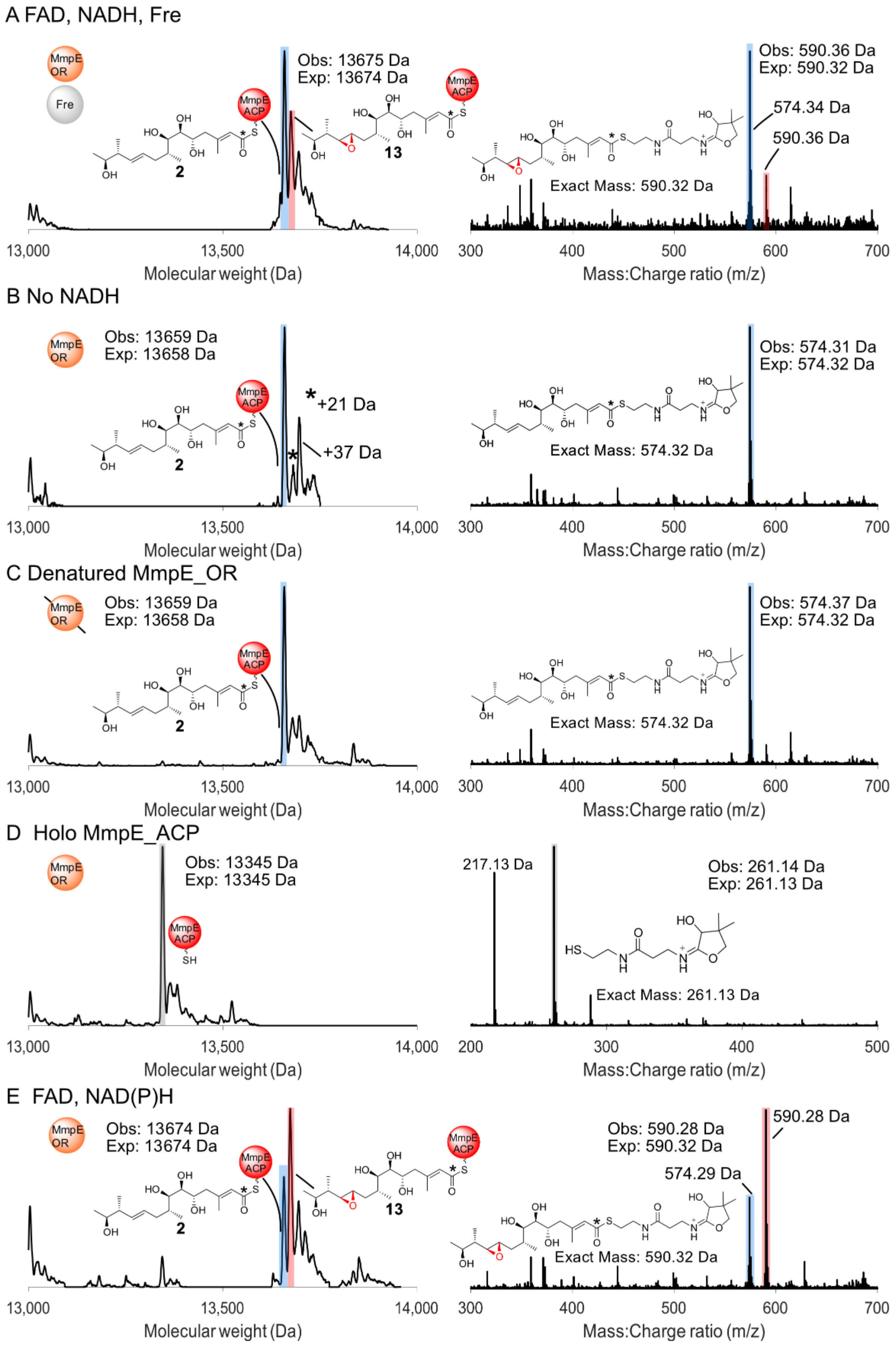

Figure S11. Control reactions of the epoxidation reaction 2-MmpE_ACP by MmpE_OR *A) Deconvoluted spectra of the epoxidation reaction **2**-MmpE_ACP with MmpE_OR in the presence of FAD, NADH and Fre to promote formation of FADH$_2$ (Obs:13,675 Da, Exp: 13,674 Da). (right): Ppant ejection ion (Obs 590.36 Da, Exp: 590.32 Da). B) Deconvoluted spectra of the epoxidation reaction with **2**-MmpE_ACP when NADH was omitted (Obs: 13,659 Da, Exp: 13,658 Da). (right): Ppant ejection (Obs: 574.31 Da, Exp: 574.32 Da). C) Deconvoluted spectra of the epoxidation reaction with **2**-MmpE_ACP when MmpE_OR was denatured (Obs: 13,659 Da, Exp: 13,658 Da). (right): Ppant ejection ion (Obs:574.37 Da, Exp: 574.32 Da). D) Deconvoluted spectra of the epoxidation reaction when MmpE_OR was incubated with holo-MmpE_ACP (Obs: 13,345 Da, Exp: 13,345 Da). (right) Ppant ejection ion (Obs: 261.14 Da, Exp: 261.13 Da). E) Deconvoluted spectra of the reaction of **2**-MmpE_ACP with MmpE_OR when incubated with FAD and NAD(P)H (Obs: 13,674 Da, Exp: 13,674 Da). (right): Ppant ejection ion (Obs: 590.28 Da, Exp: 590.32 Da). Species are highlighted as apo (pink), holo (grey), alkene/**2** (blue), epoxide/**13** (red) for MmpE_ACP. * = ^{13}C label.*

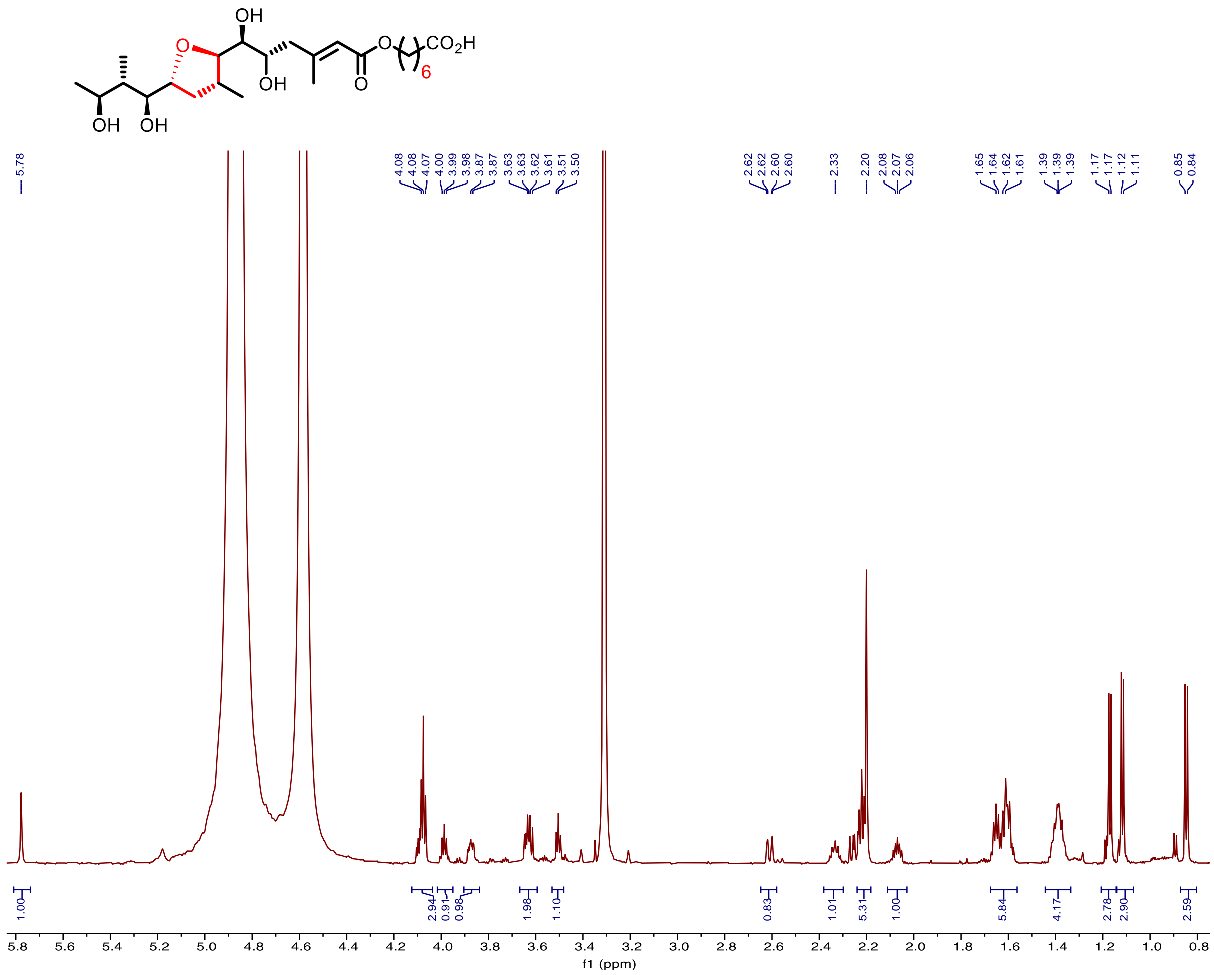

Figure S12. ^{1}H NMR spectrum of Mupirocin W2. Mupirocin W2 isolated from MmpE_OR assays measured in CD_3OD at 700 MHz.

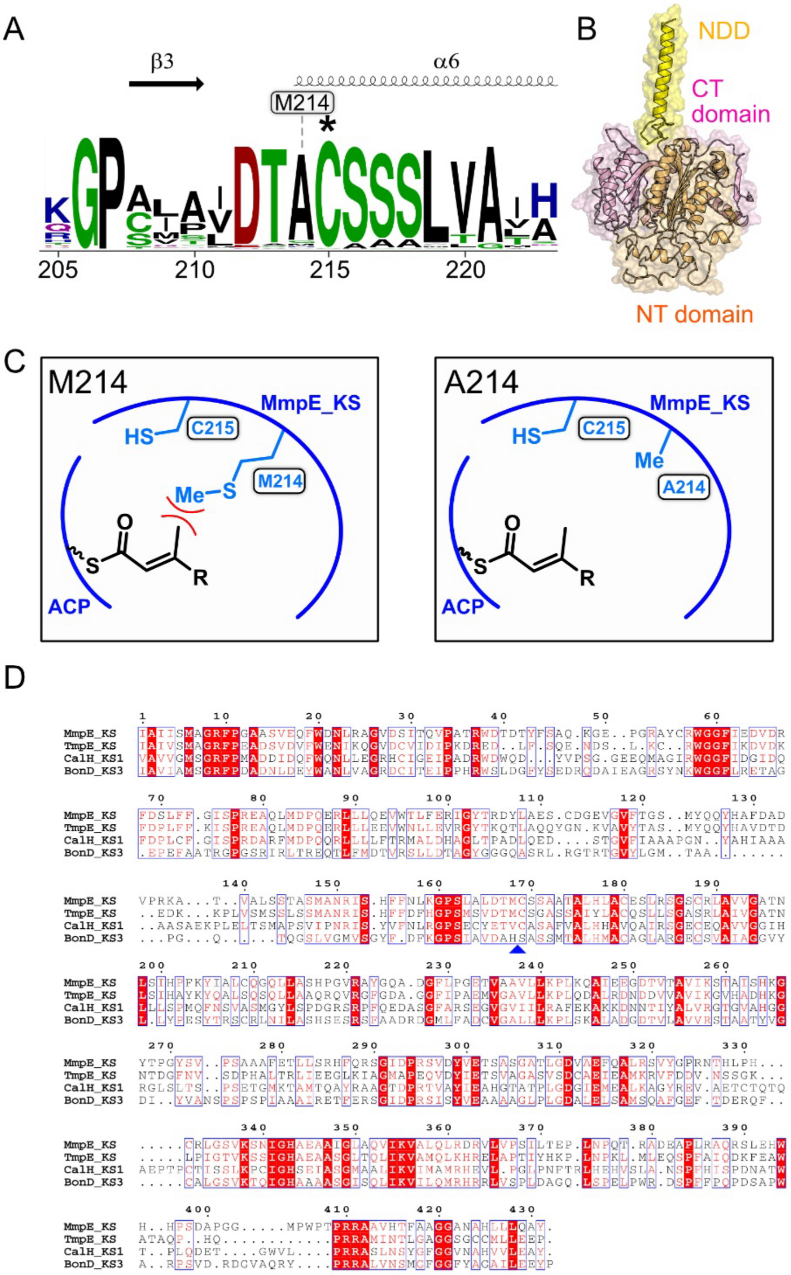

***Figure S13. Analysis of the X-Cys motif in Trans-AT KS domains immediately downstream of acceptor ACPs**. A) Web logo of sequences of 64 trans-AT KS domains immediately downstream of acceptor ACPs. The immediate sequence flanking the active site cysteine is highlighted. Predicted secondary structure of MmpE_KS is highlighted as well as the M214 residue. * refers to the conserved active site cysteine. B) Ab initio homology model of MmpE_KS depicting the ^{N}DD motif, N-terminal KS core domain and C-terminal adapter domain in yellow, orange and pink respectively. C) Cartoon schematic depicting how a methionine residue preceding the active site cysteine may block or occlude a β-branched intermediate, in comparison to the less bulky alanine residue within the KS active site. D) Multiple sequence alignment of MmpE_KS, TmpE_KS, CalH_KS1 and BonD_KS3. The active site X-Cys motif is highlighted by a blue arrow.*

NMR spectra to support synthesis:

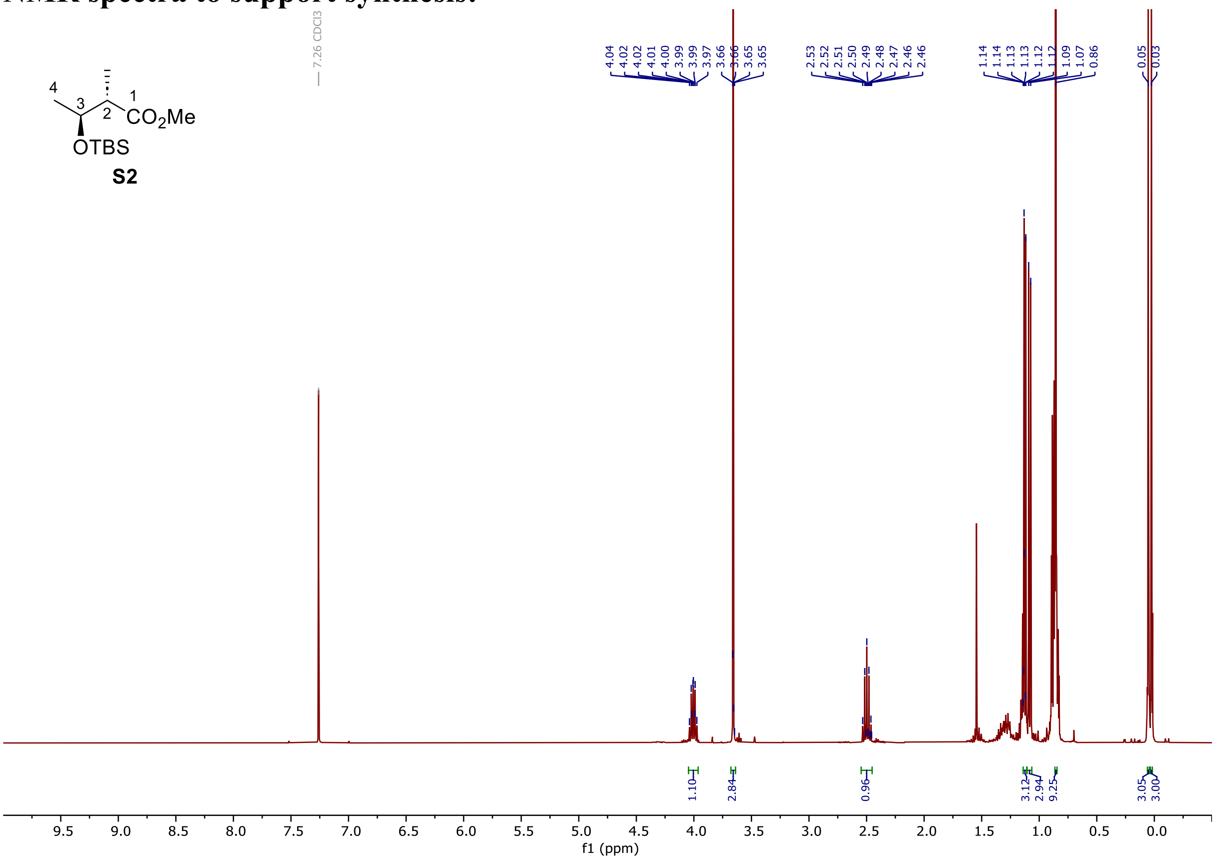

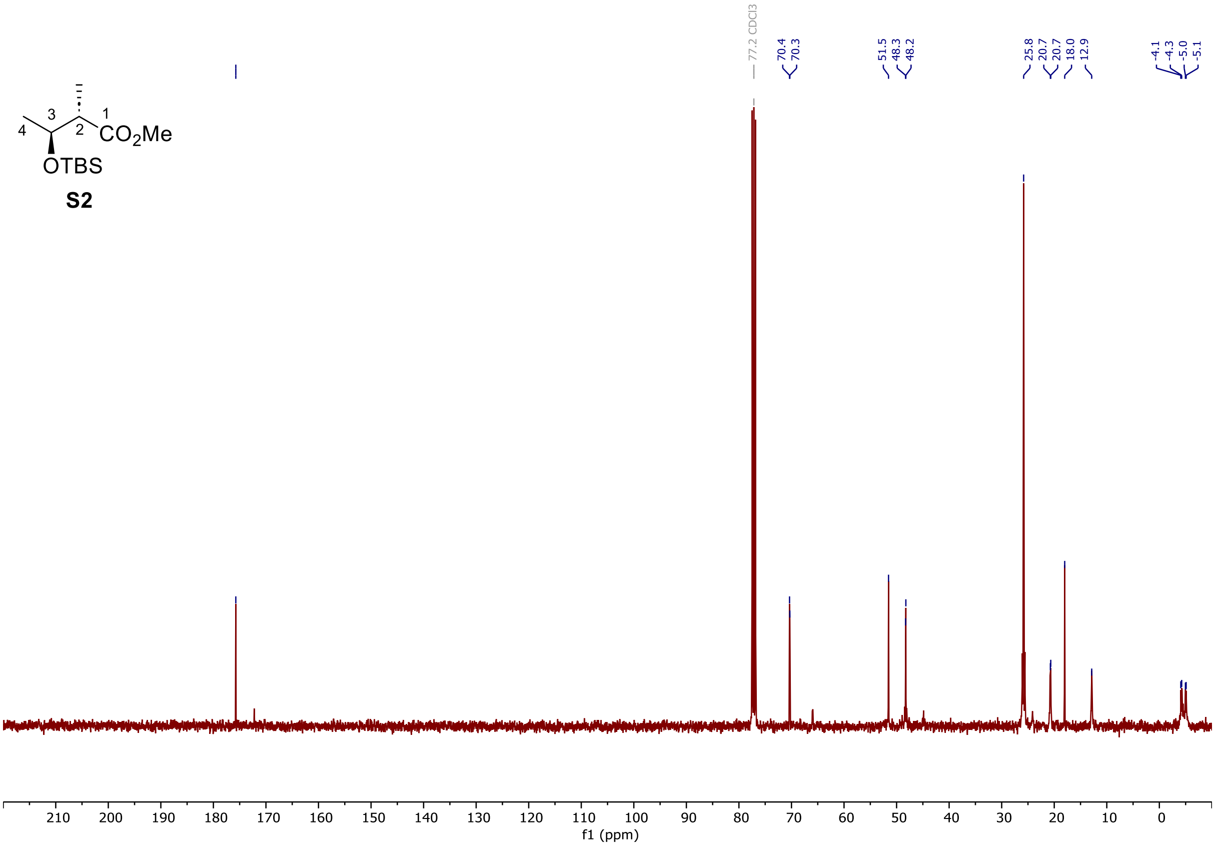

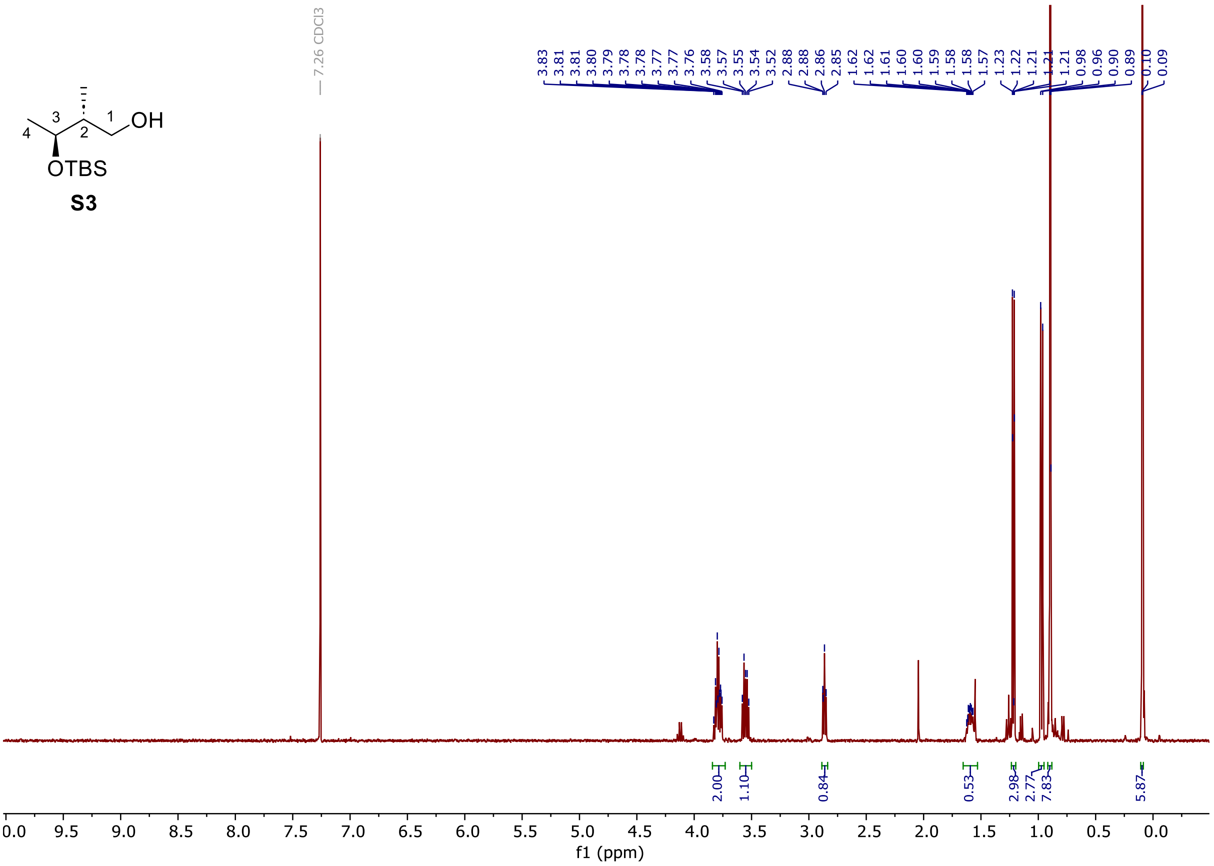

4
3
2
1
OH
OTBS
7.26 CDCl3
3.83 3.81 3.81 3.80 3.79 3.78 3.78 3.77 3.77 3.76 3.58 3.57 3.55 3.54 3.52 2.88 2.88 2.86 2.85 1.62 1.62 1.61 1.60 1.60 1.59 1.58 1.58 1.57 1.23 1.22 1.21 1.21 1.21 0.98 0.96 0.90 0.89 0.10 0.09
2.00 1.10 0.84 0.53 2.98 2.77 7.83 5.87
f1 (ppm)

S3

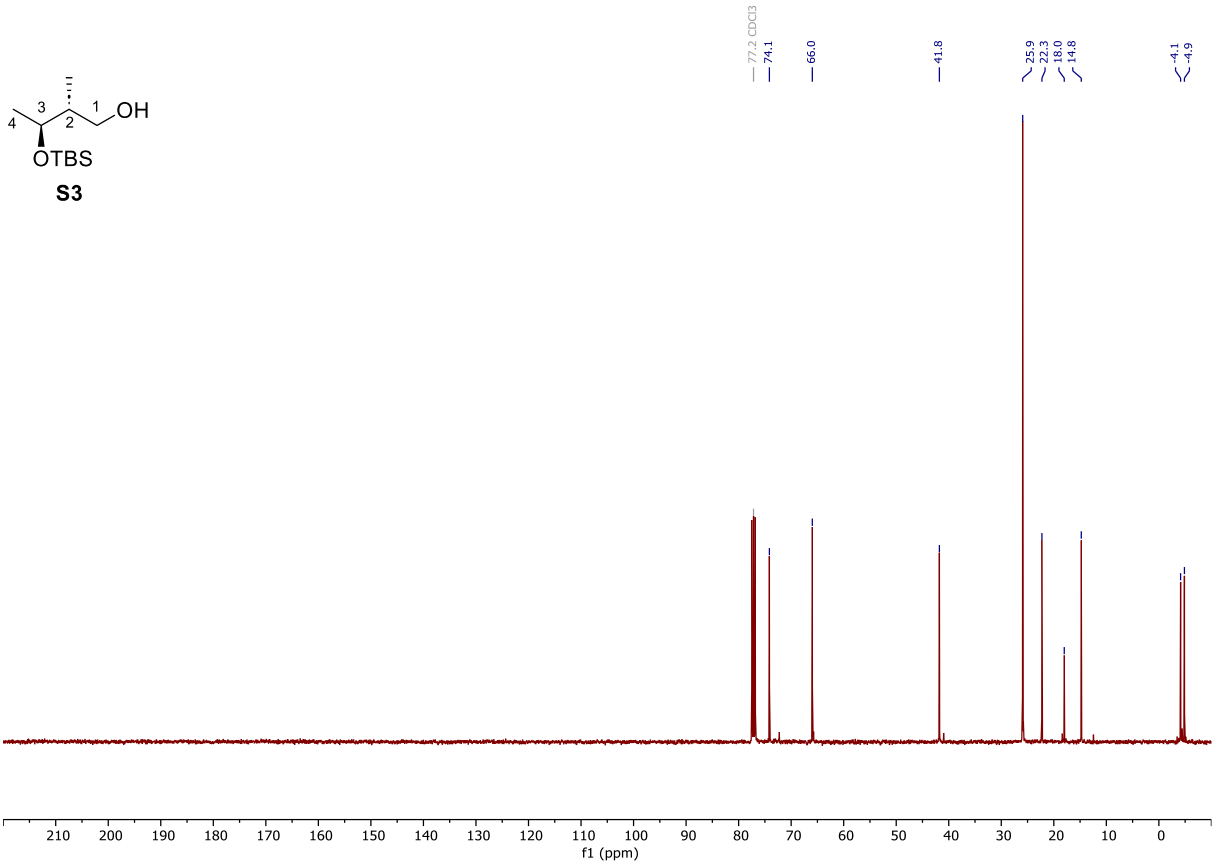

4
3
2
1
OH
OTBS
77.2 CDCl3
74.1
66.0
41.8
25.9
22.3
18.0
14.8
-4.1
-4.9
f1 (ppm)

S3

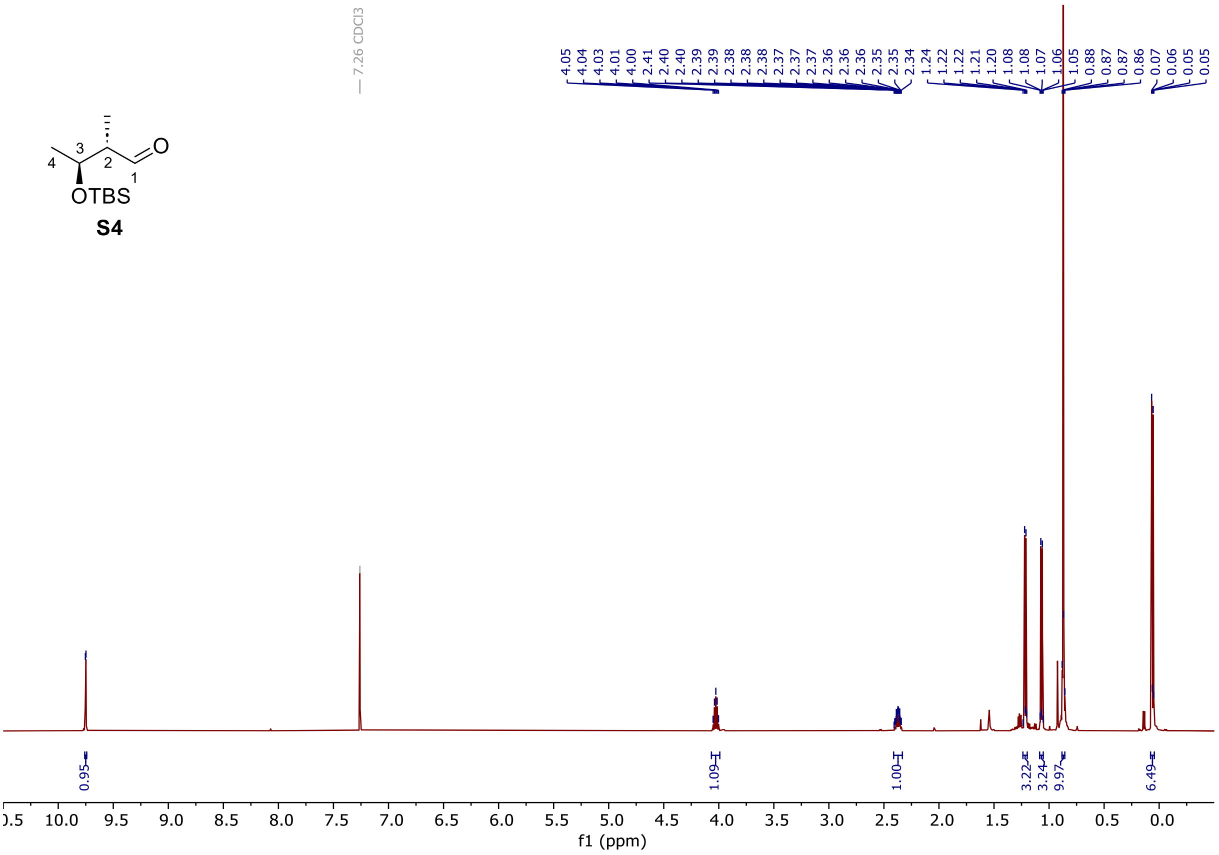
S4
OTBS
f1 (ppm)
7.26 CDCl3

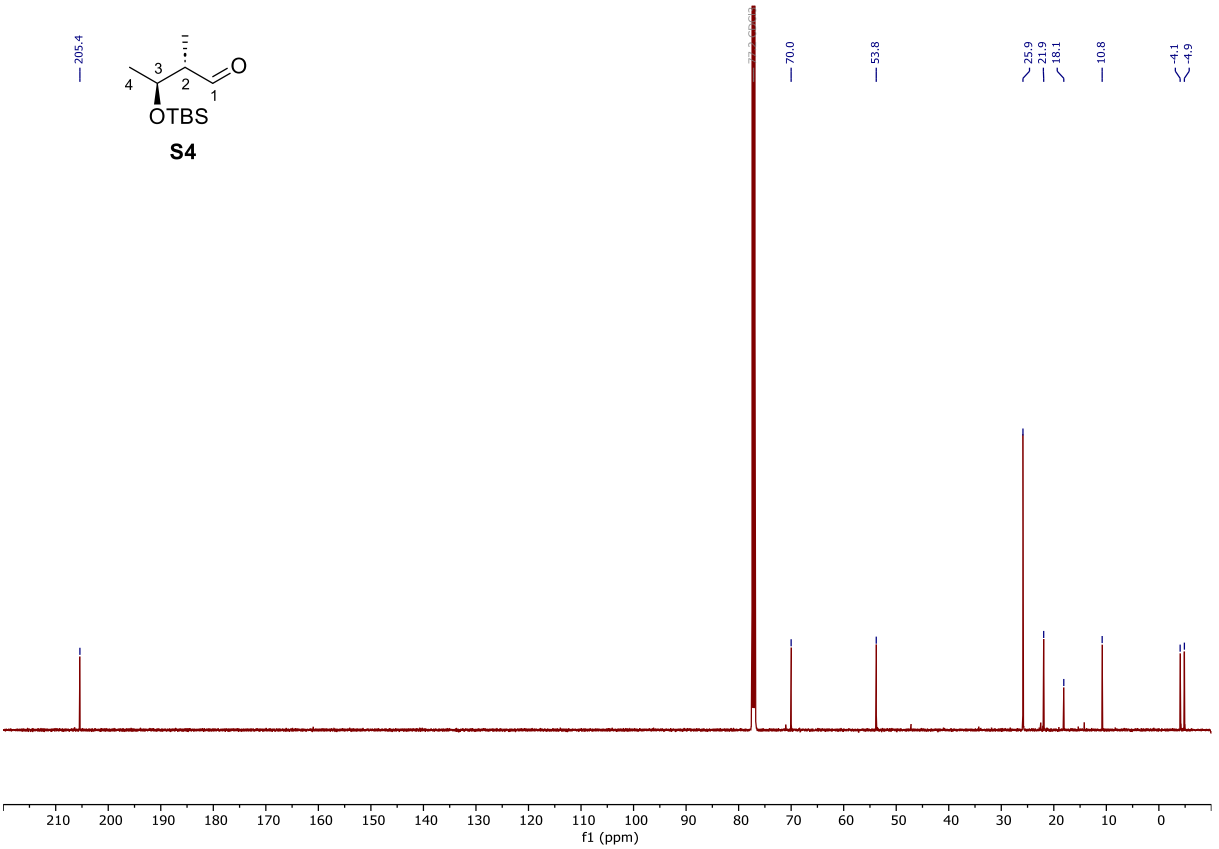
S4
OTBS
f1 (ppm)

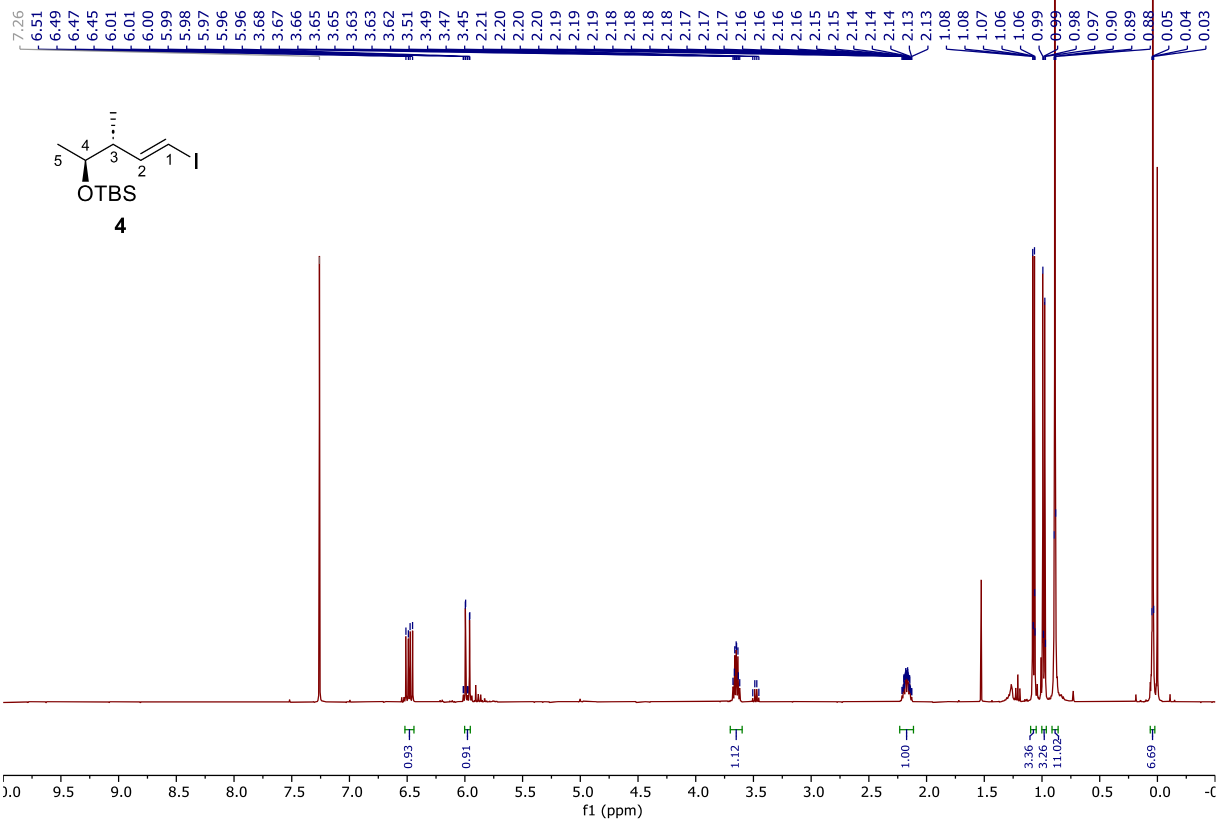

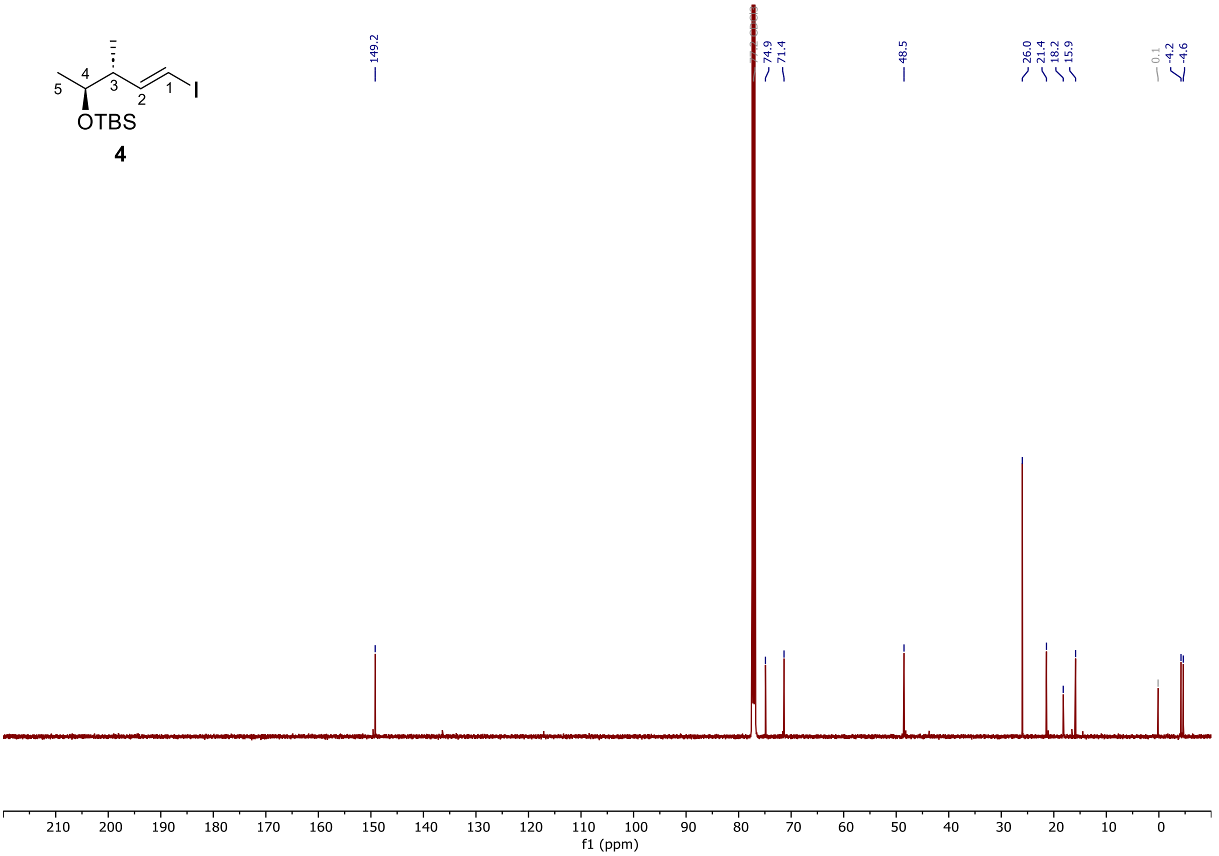

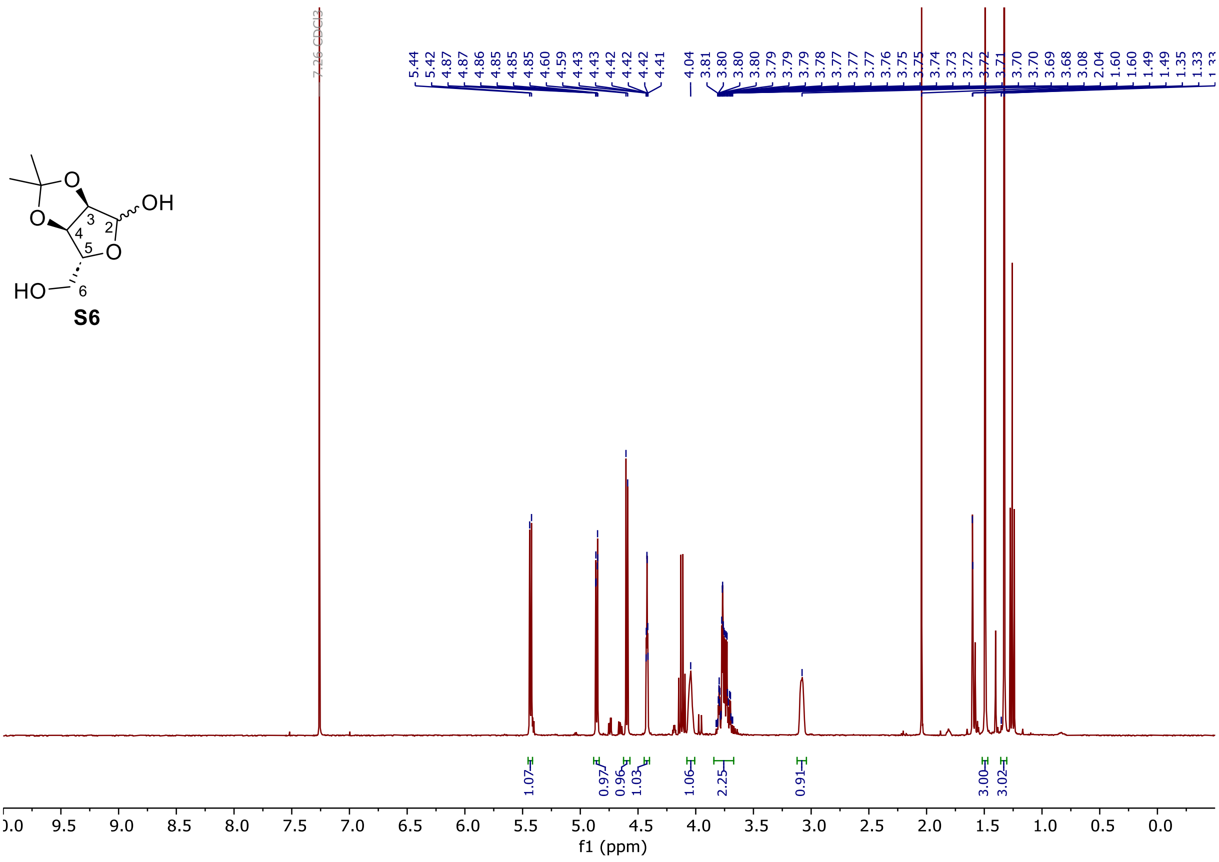
S6
7.26 CDCl3
5.44 5.42 4.87 4.87 4.86 4.85 4.85 4.85 4.60 4.59 4.43 4.43 4.42 4.42 4.42 4.41 4.04 3.81 3.80 3.80 3.80 3.79 3.79 3.79 3.78 3.77 3.77 3.77 3.76 3.75 3.75 3.74 3.73 3.72 3.72 3.71 3.70 3.70 3.69 3.68 3.08 2.04 1.60 1.60 1.49 1.35 1.33
1.07 0.97 0.96 1.03 1.06 2.25 0.91 3.00 3.02
f1 (ppm)

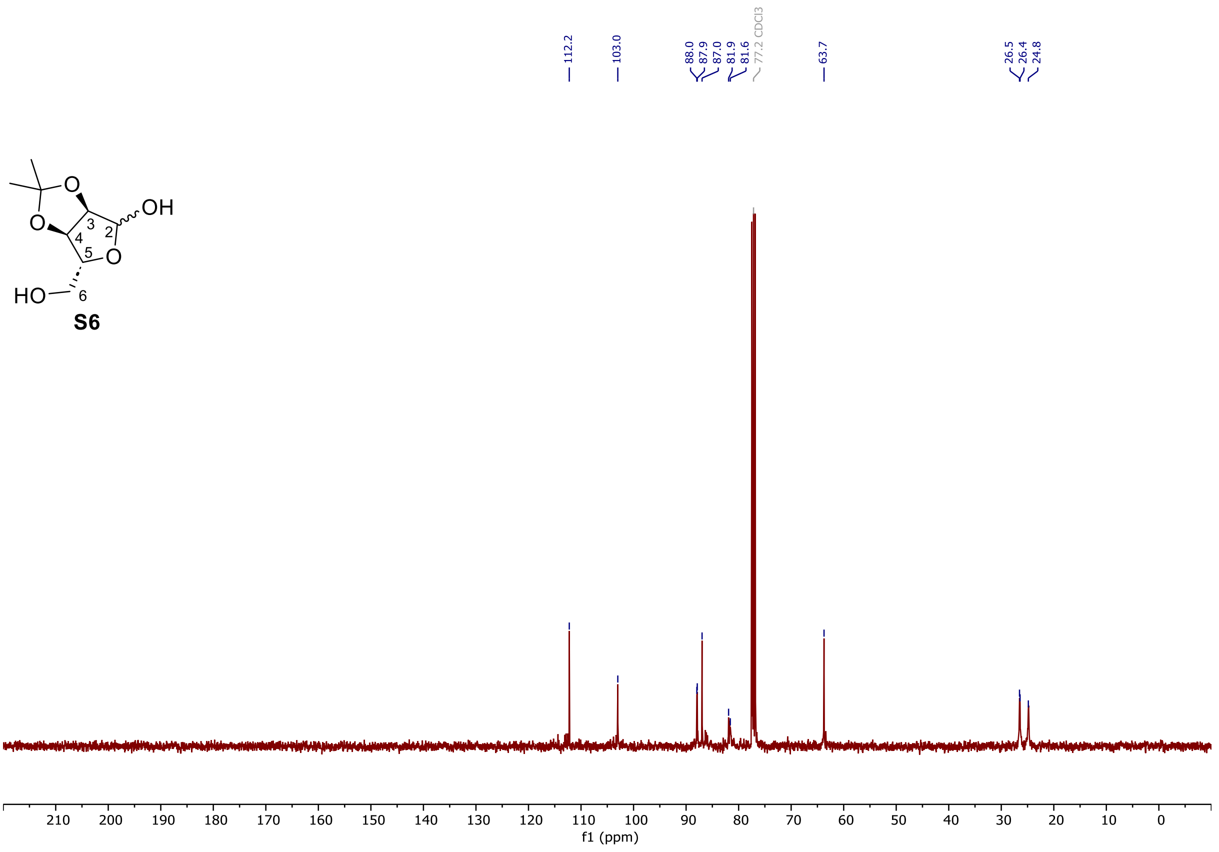
S6
112.2 103.0 88.0 87.9 87.0 81.9 81.6 77.2 CDCl3 63.7 26.5 26.4 24.8
f1 (ppm)

δ$_H$ (500 MHz, $CDCl_3$)

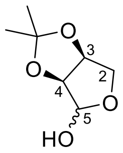

S8

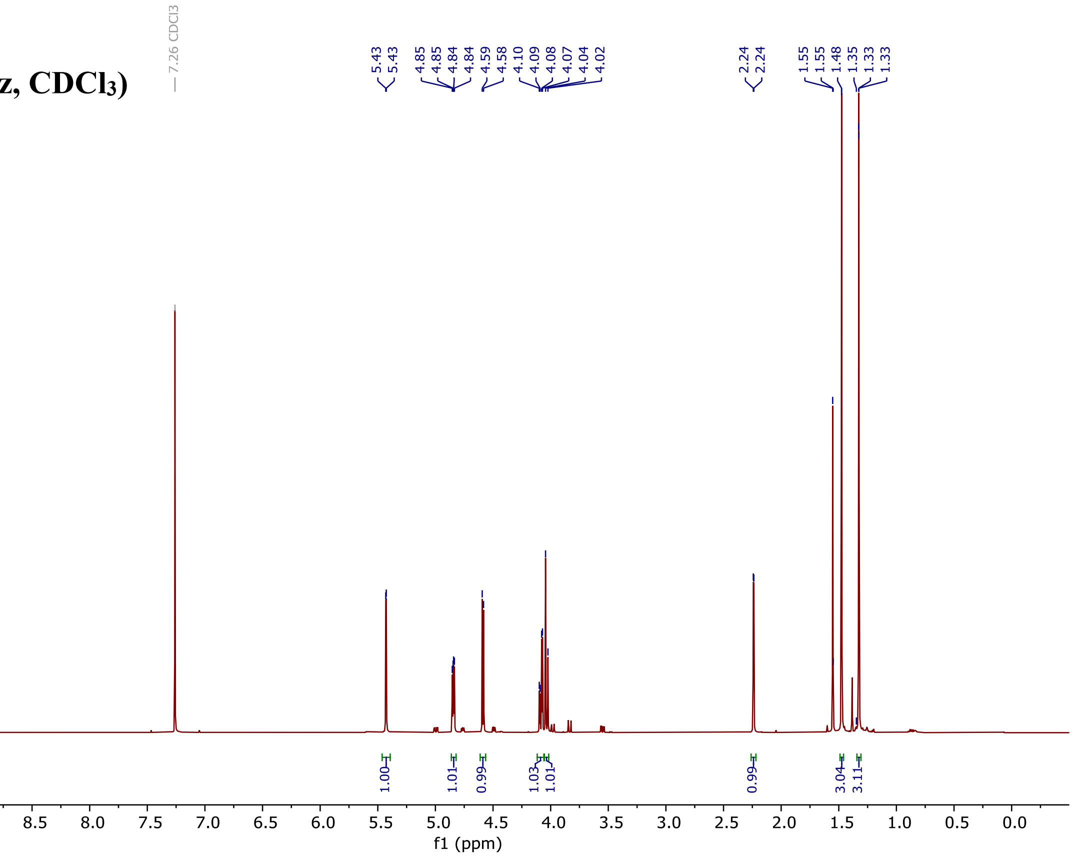

δ$_C$ (126 MHz, $CDCl_3$)

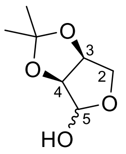

S8

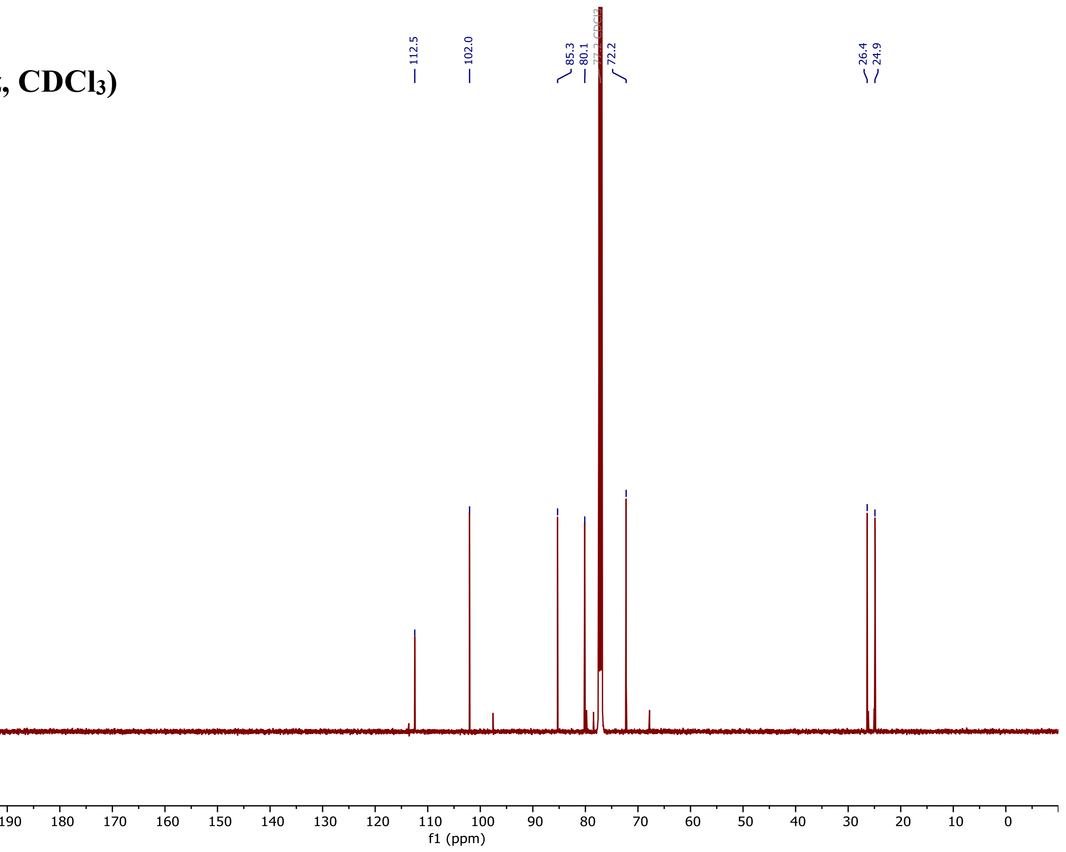

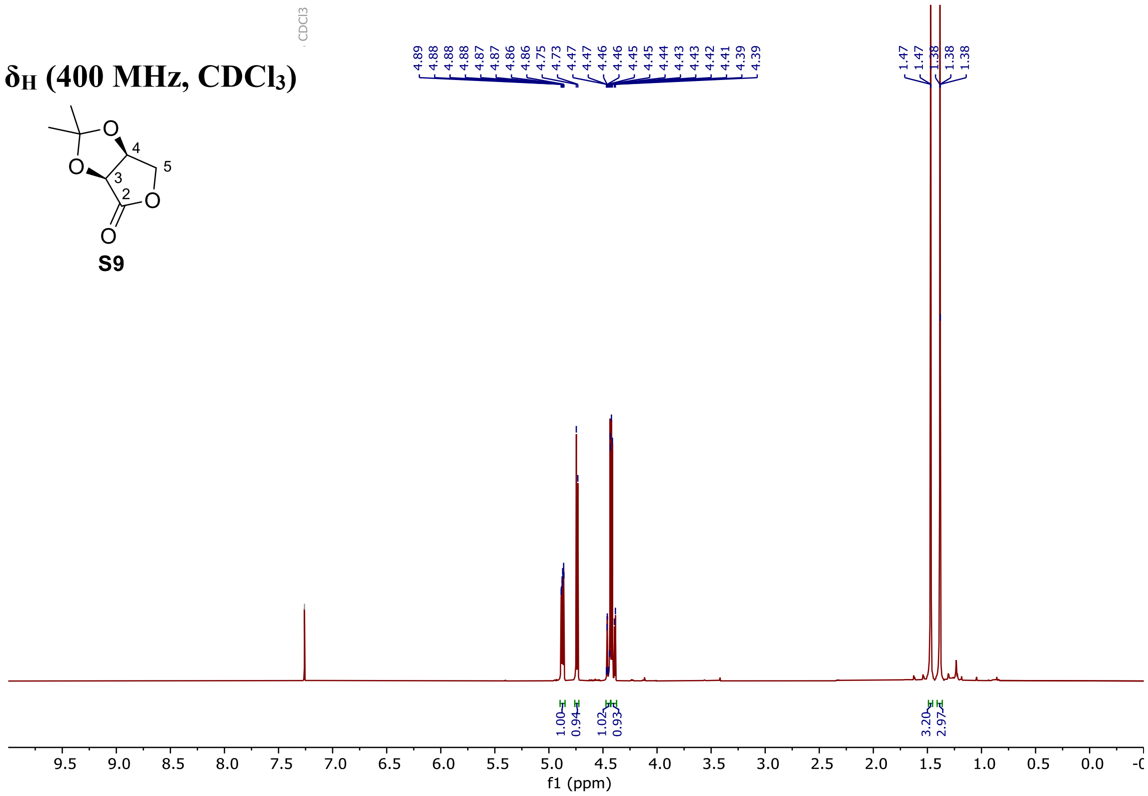
δH (400 MHz, CDCl3)
S9
CDCl3
4.89 4.88 4.88 4.88 4.87 4.87 4.86 4.75 4.73 4.47 4.47 4.46 4.46 4.45 4.44 4.43 4.43 4.42 4.41 4.39 4.39
1.47 1.47 1.38 1.38 1.38
1.00 0.94 1.02 0.93 3.20 2.97
9.5 9.0 8.5 8.0 7.5 7.0 6.5 6.0 5.5 5.0 4.5 4.0 3.5 3.0 2.5 2.0 1.5 1.0 0.5 0.0
f1 (ppm)

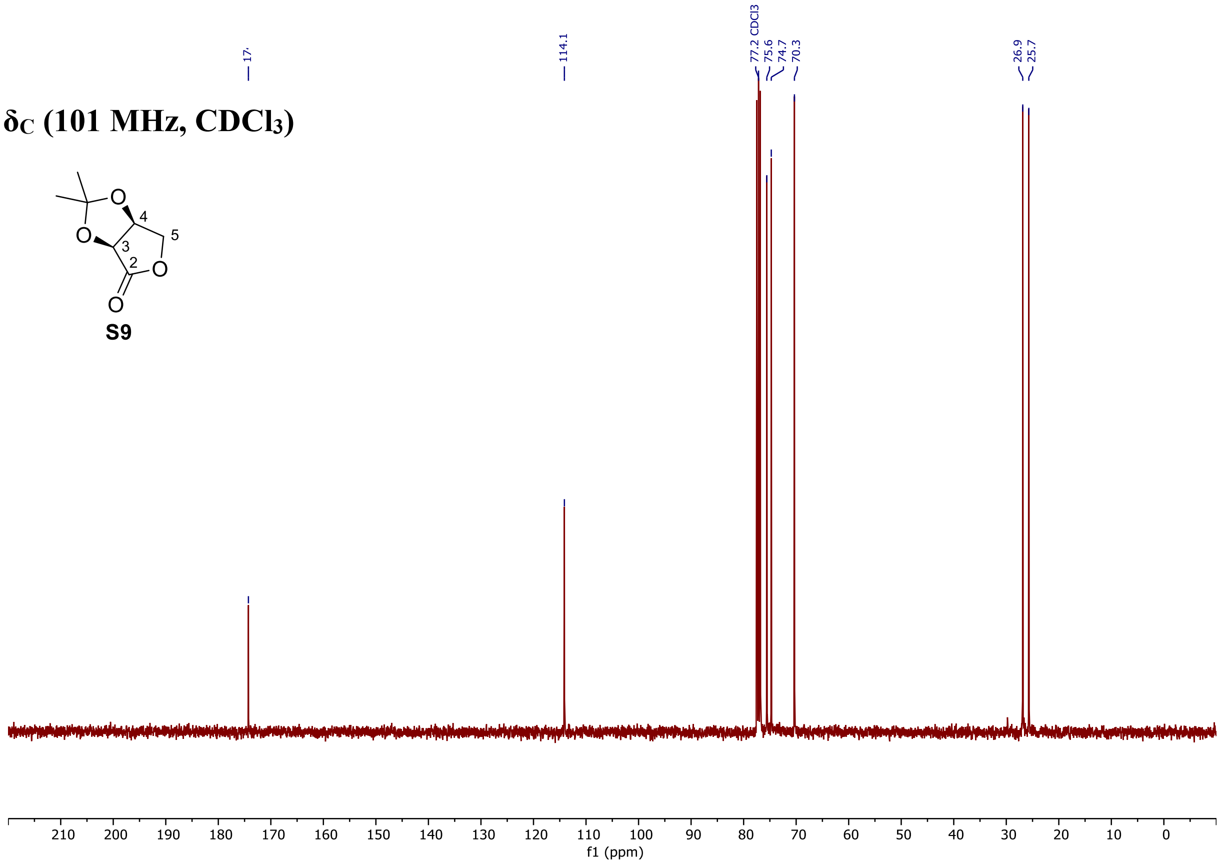
δC (101 MHz, CDCl3)
S9
114.1
77.2 CDCl3
75.6
74.7
70.3
26.9
25.7
210 200 190 180 170 160 150 140 130 120 110 100 90 80 70 60 50 40 30 20 10 0
f1 (ppm)

δH (500 MHz, CDCl3)

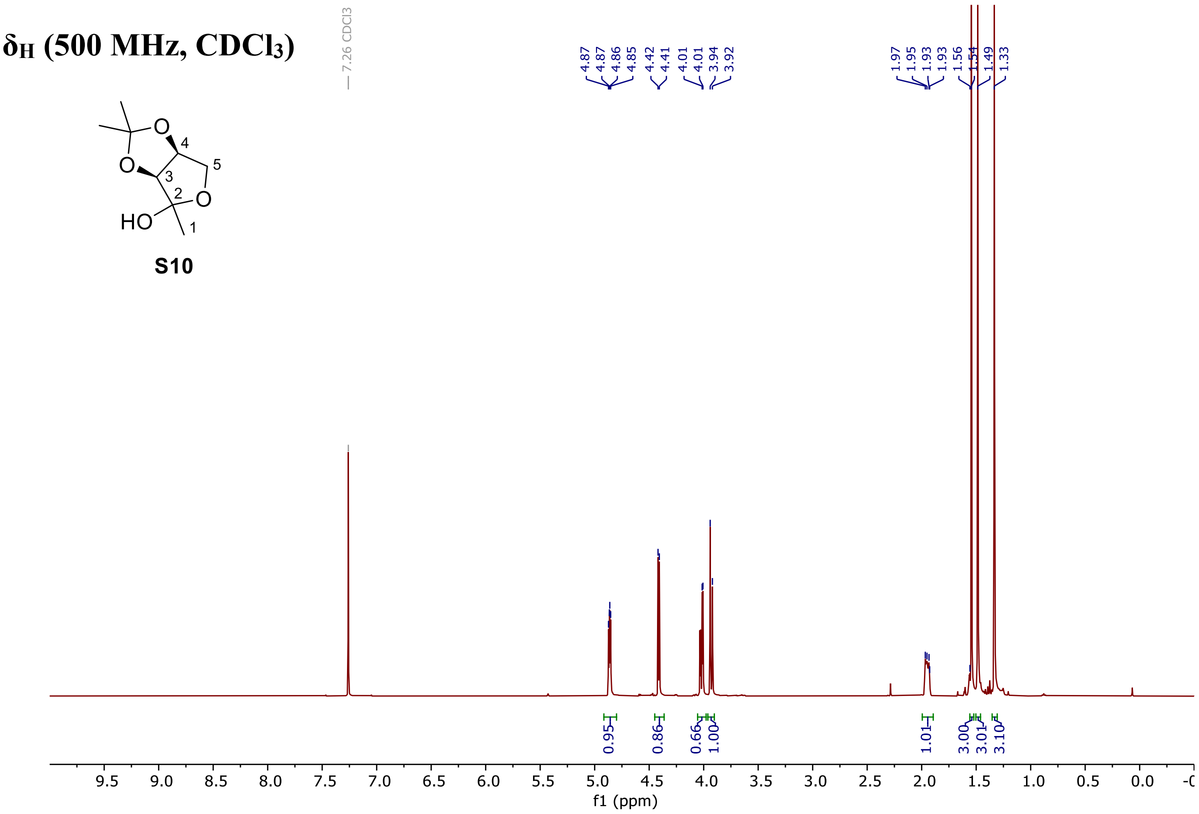

δC (126 MHz, CDCl3)

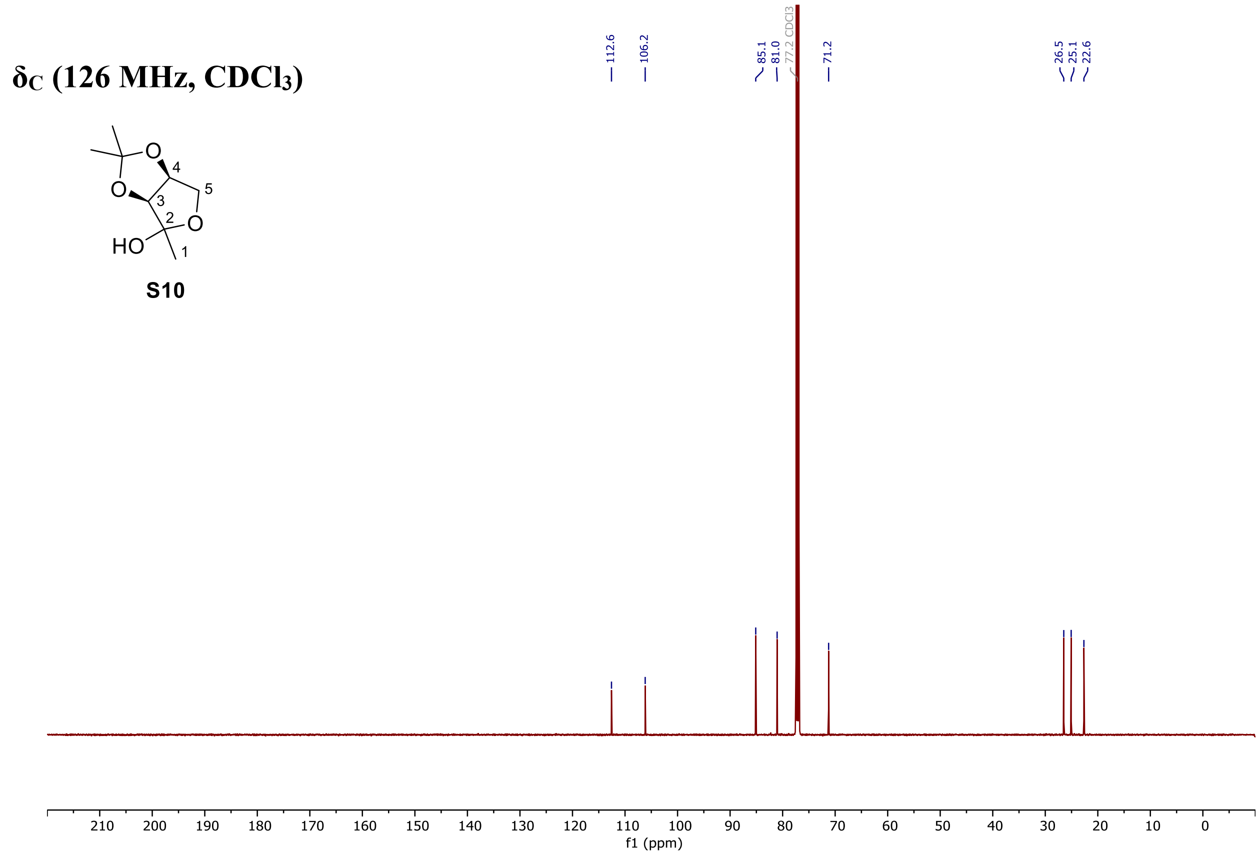

δ$_H$ (500 MHz, CDCl$_3$)

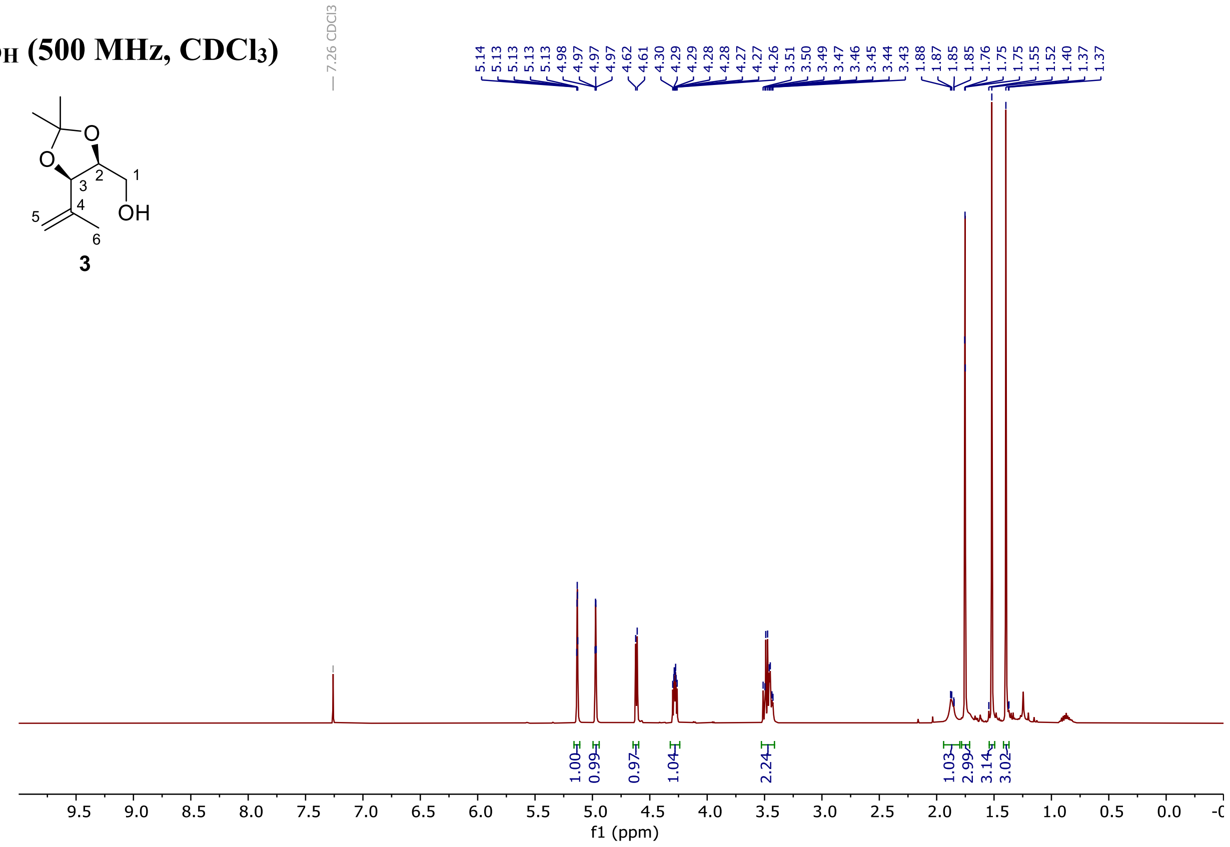

δ$_C$ (126 MHz,

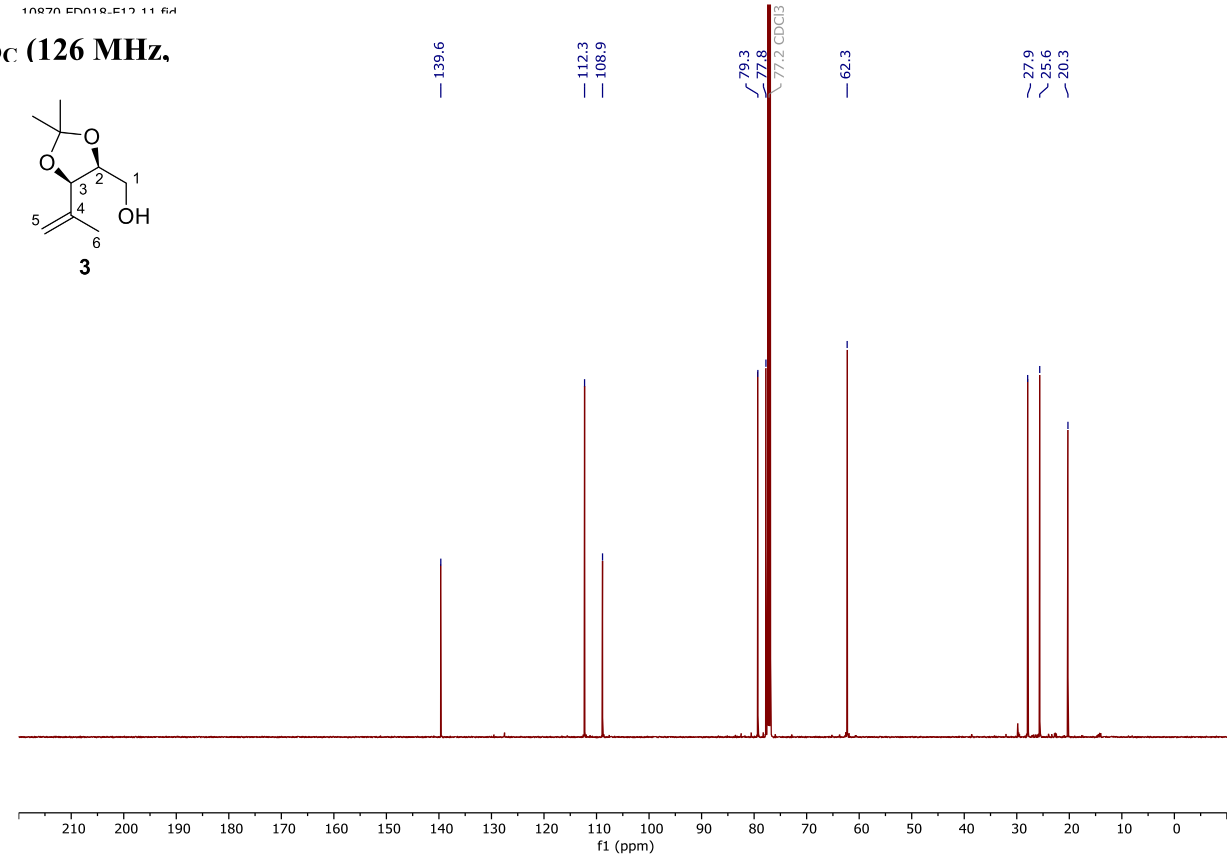

δ$_H$ (400 MHz, $CDCl_3$)

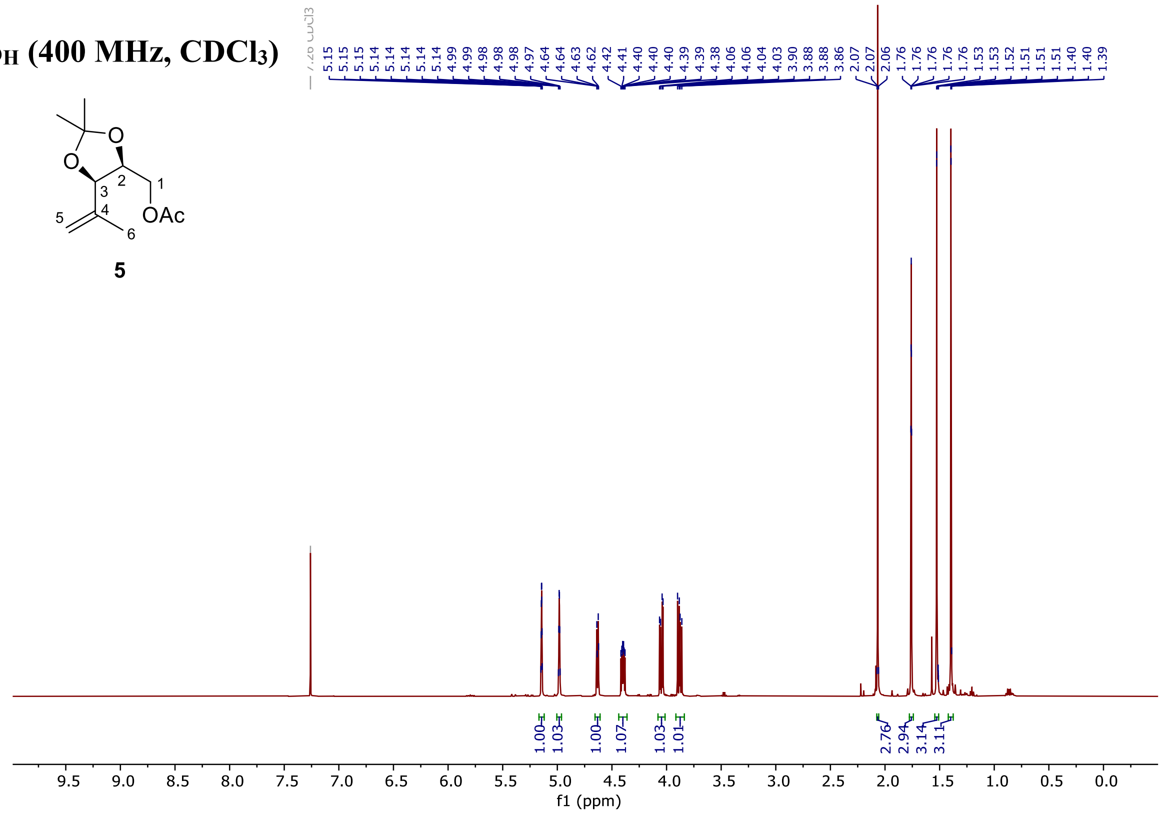

δ$_C$ (101 MHz, $CDCl_3$)

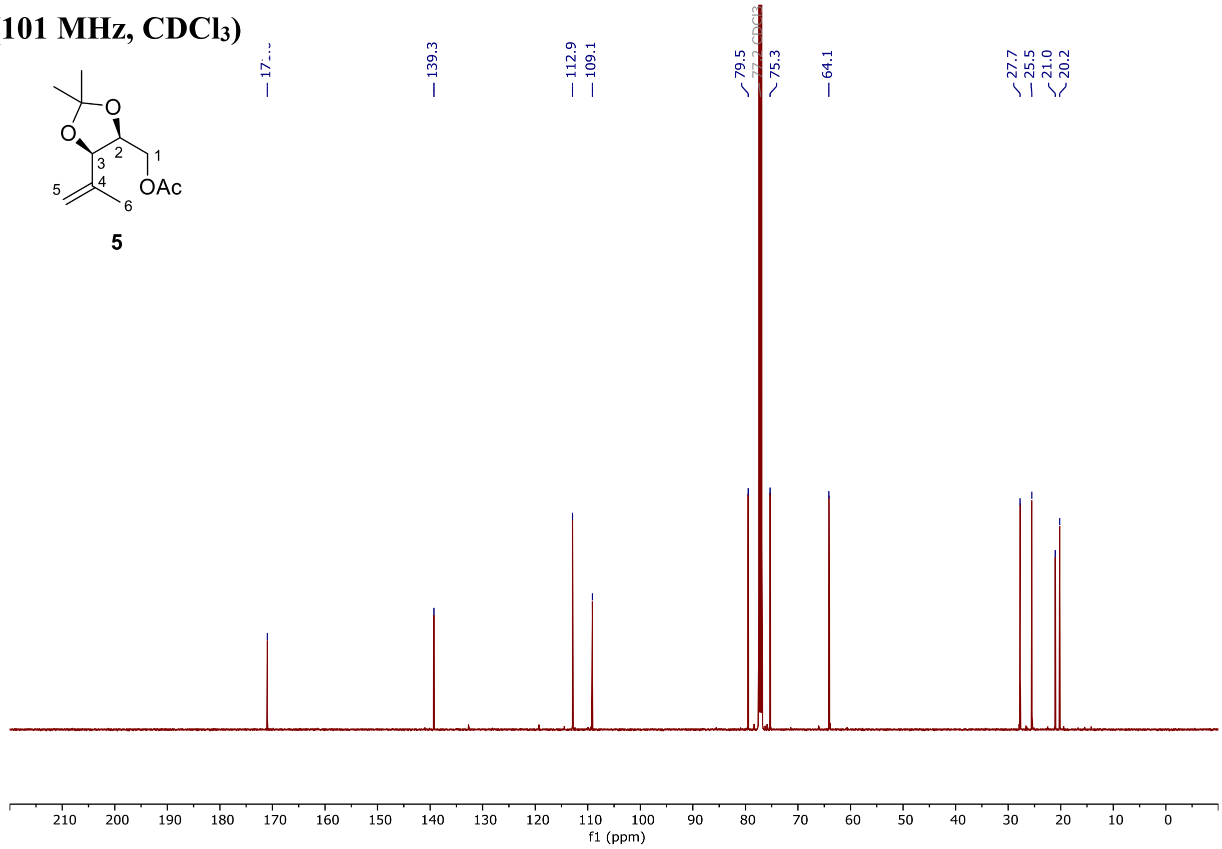

δH (500 MHz, CDCl3)

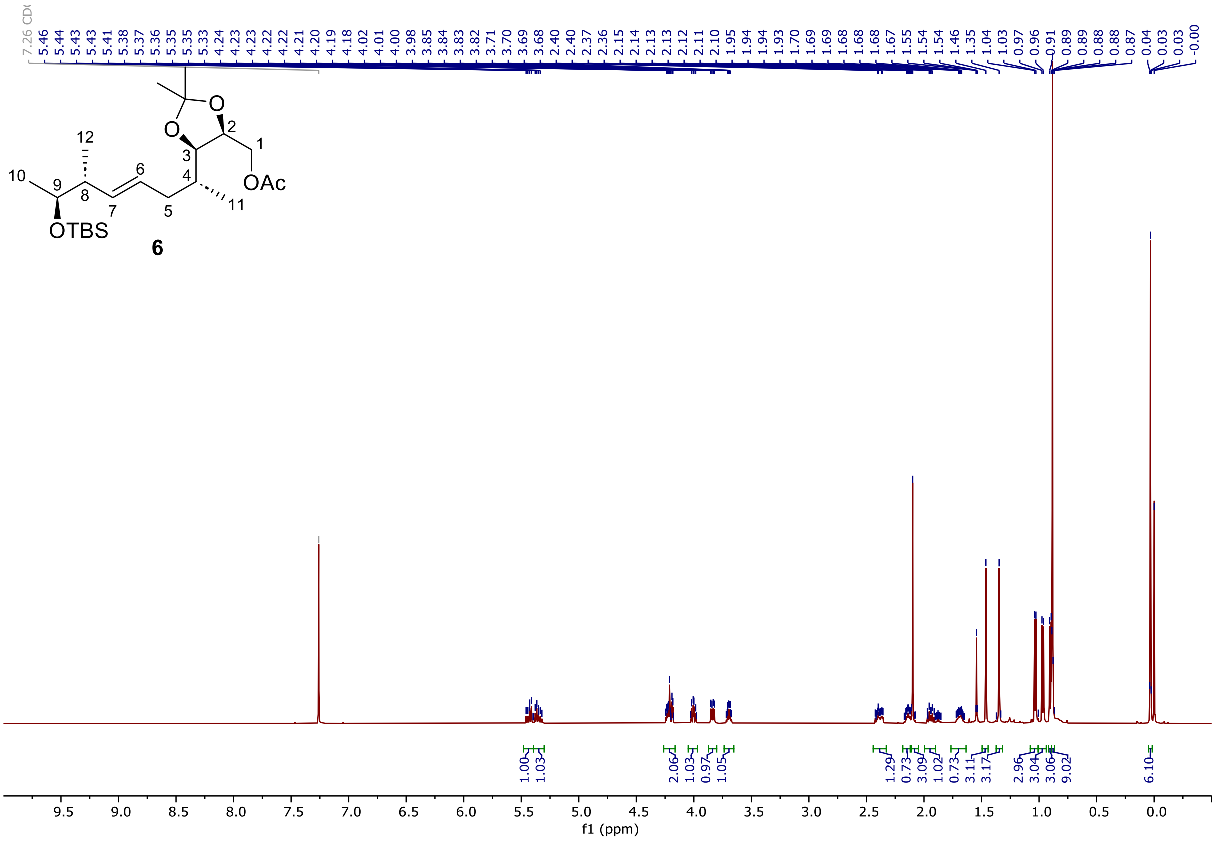

δC (126 MHz, CDCl3)

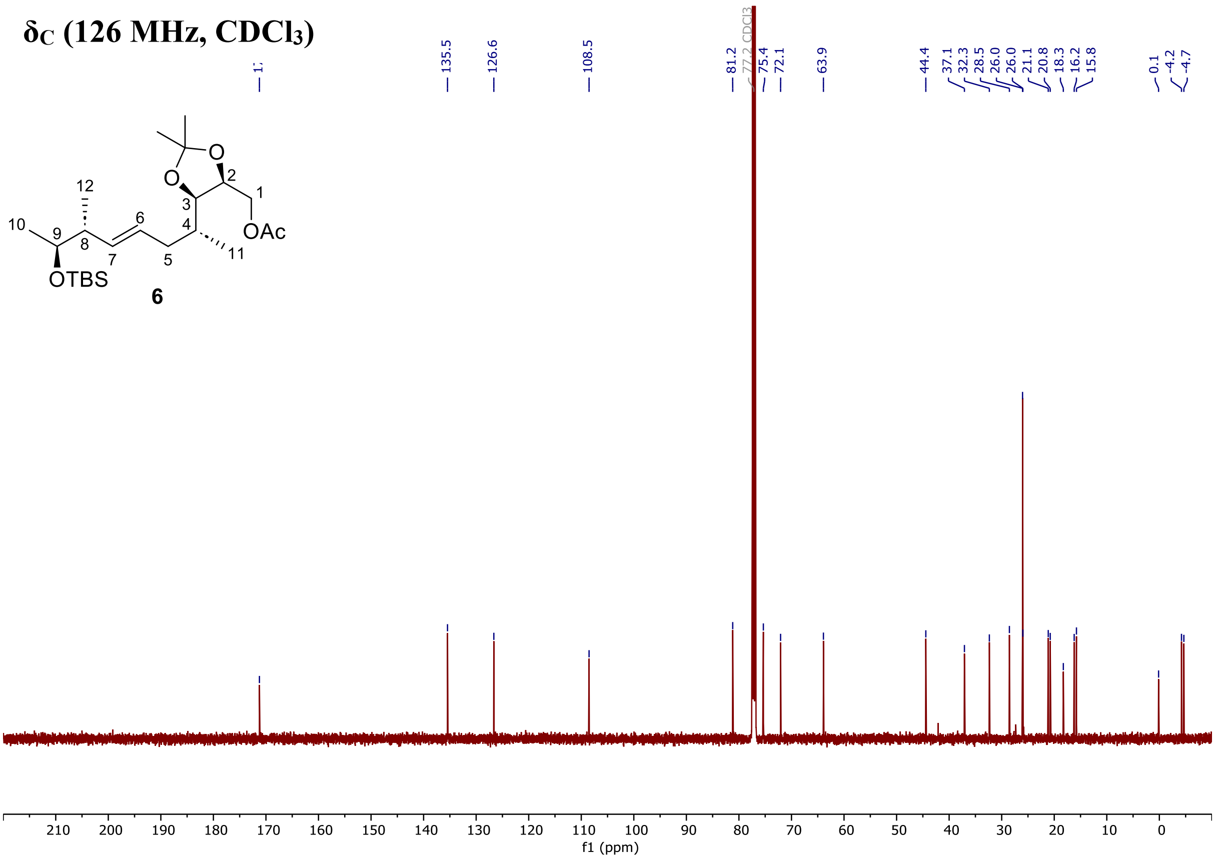

δH (500 MHz, CDCl3)

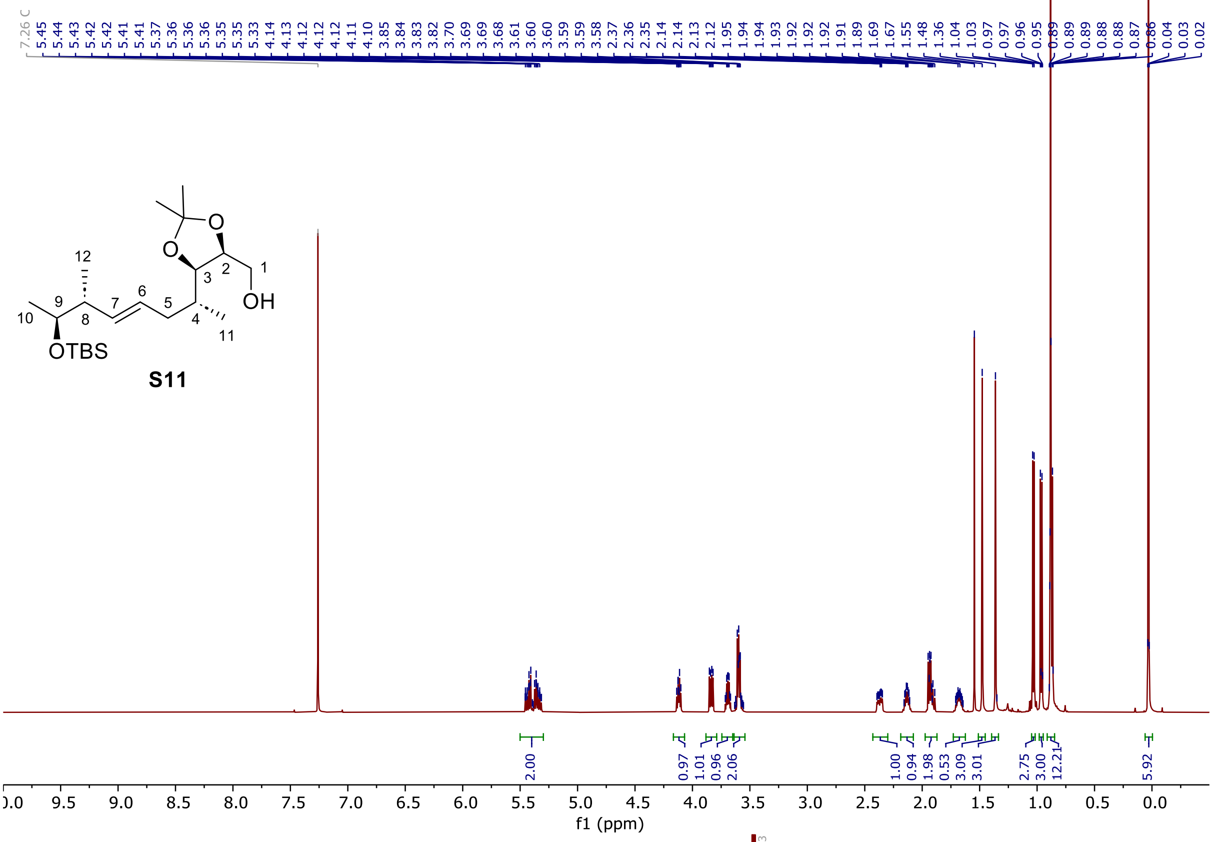

δc (126 MHz, CDCl3)

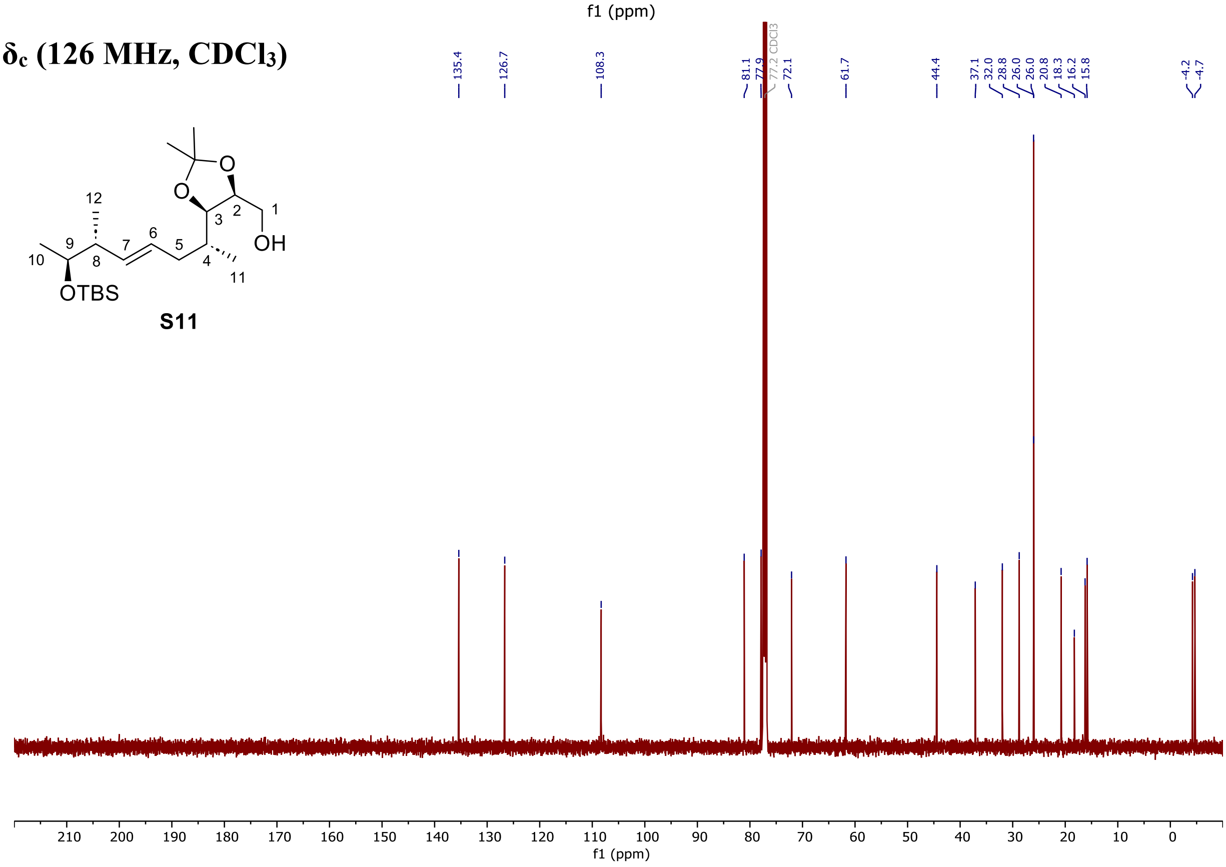

δ_H (500 MHz, $CDCl_3$)

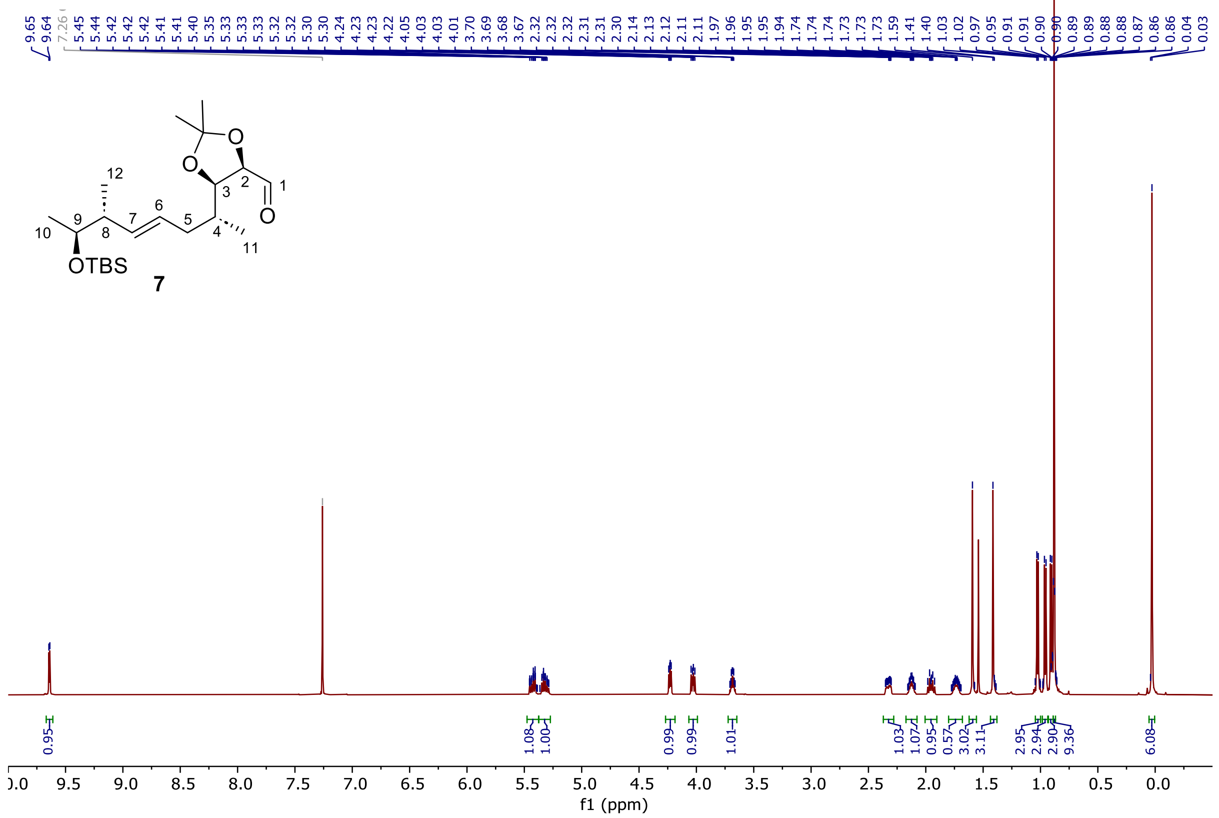

δ_C (126 MHz, $CDCl_3$)

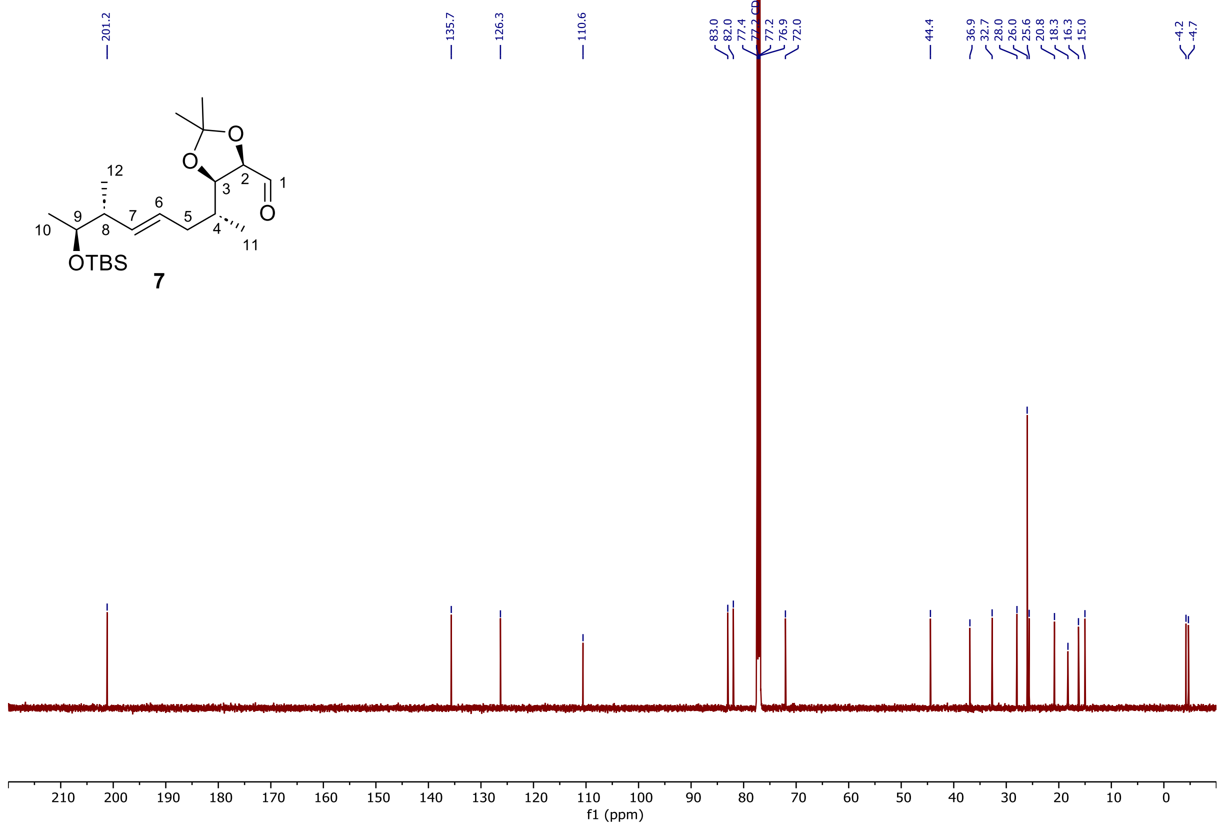

δ$_H$ (500 MHz, CDCl$_3$)

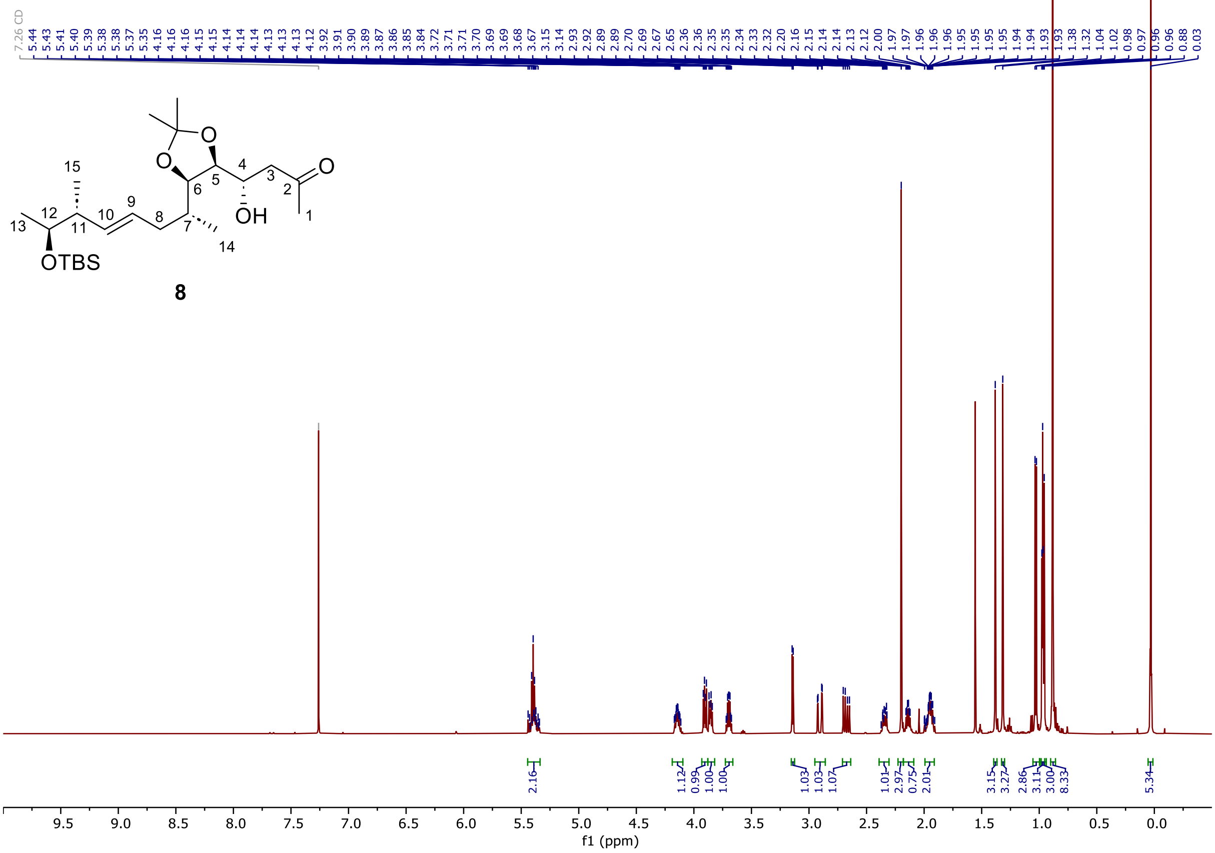

δ$_c$ (126 MHz, CDCl$_3$)

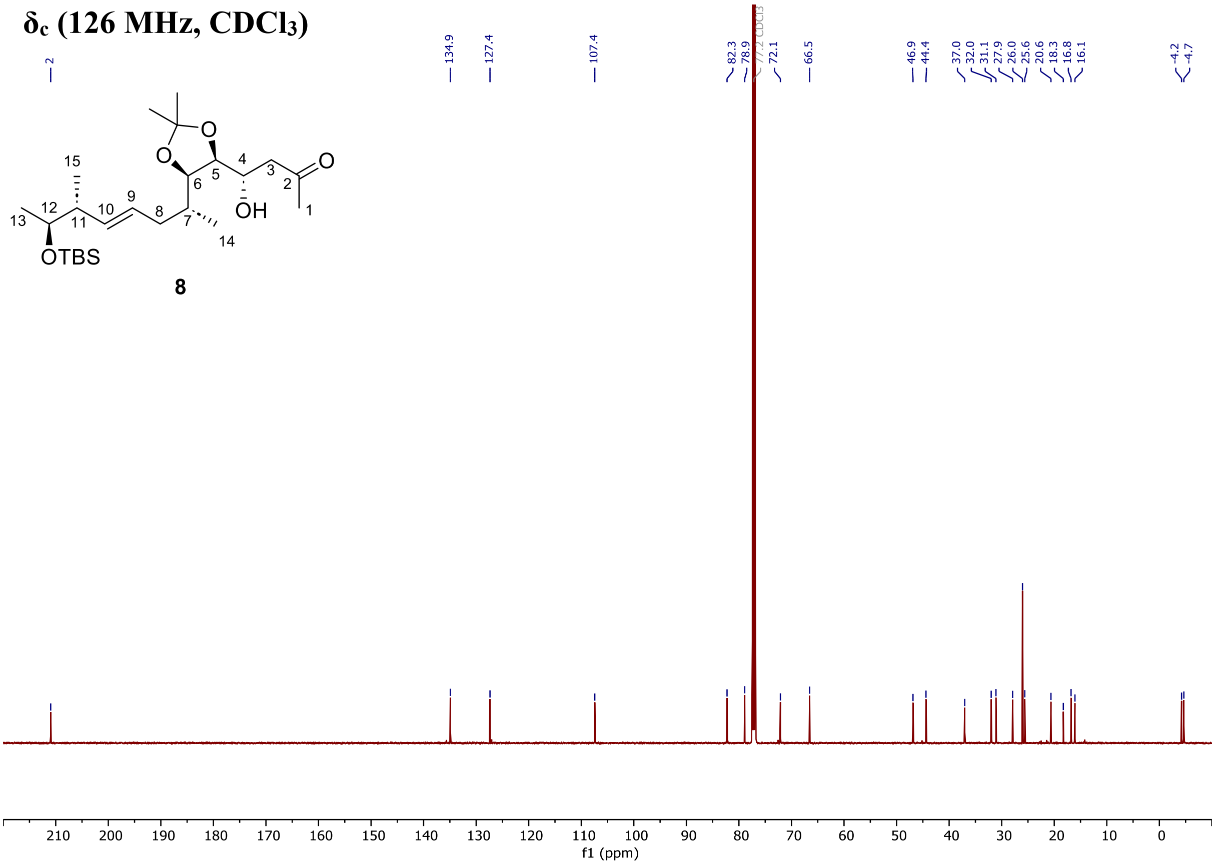

δH (600 MHz, CDCl3)

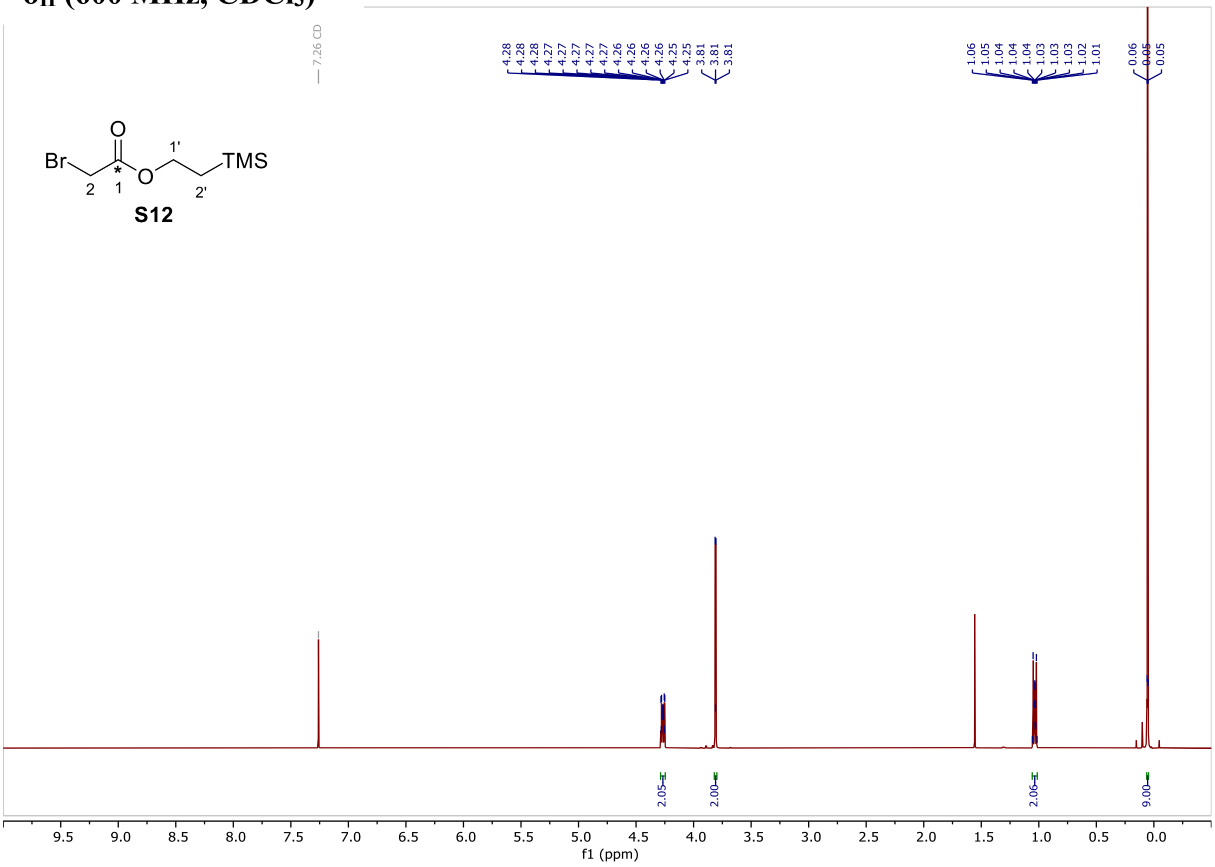

δH (151 MHz, CDCl3)

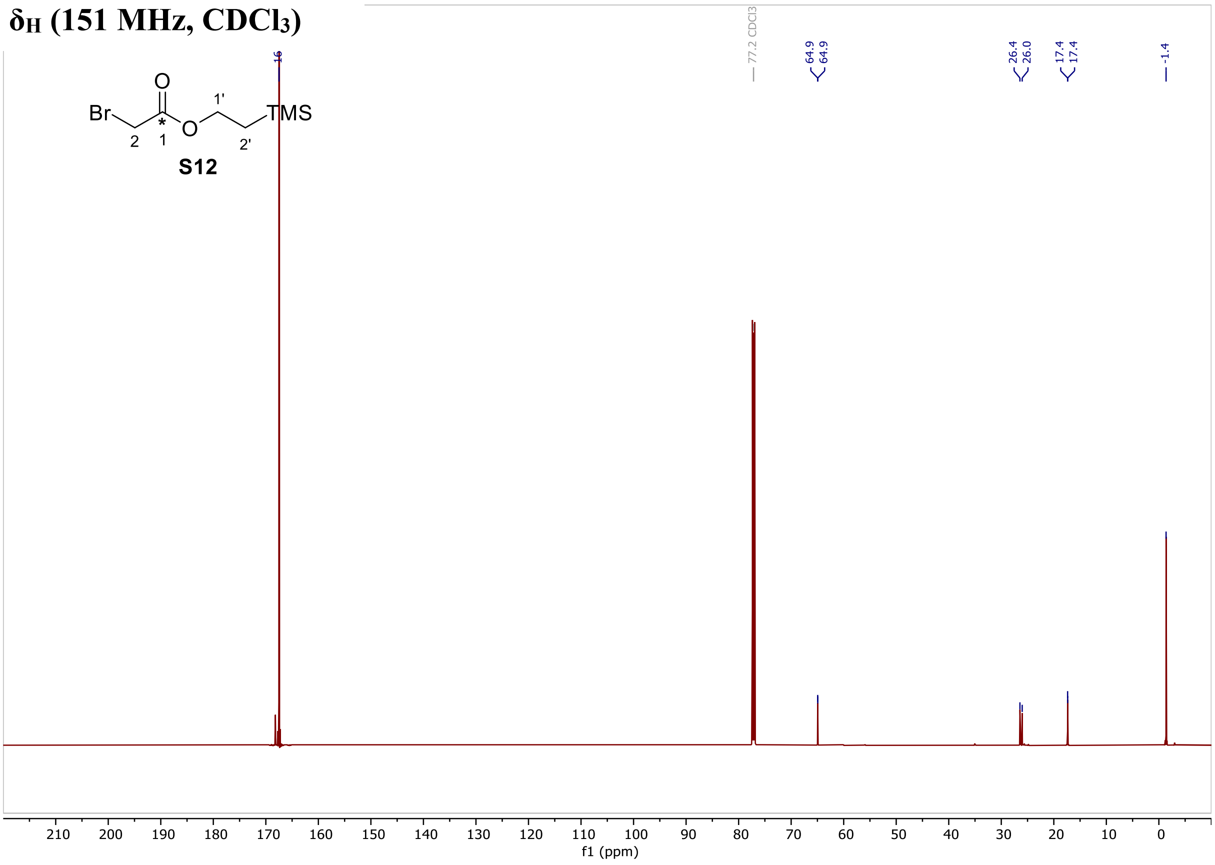

δ_H (600 MHz, $CDCl_3$)

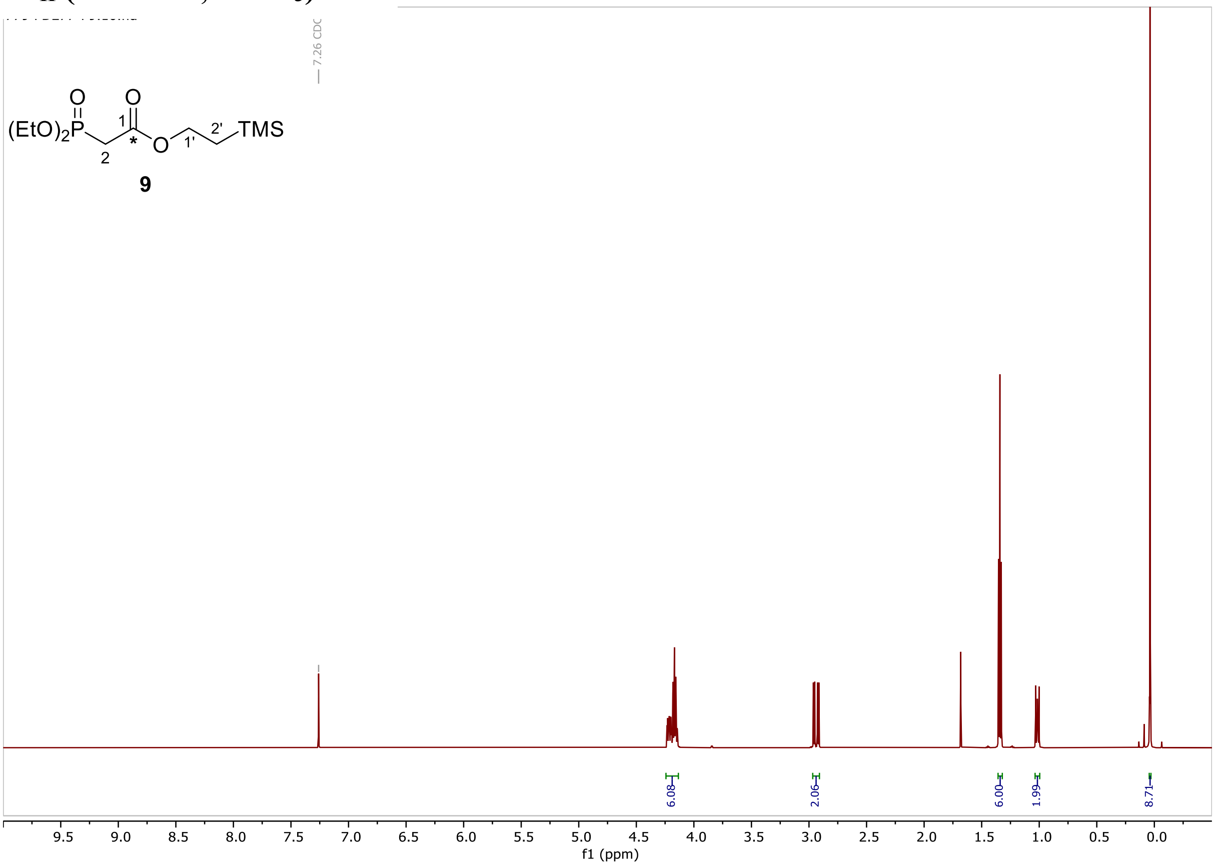

δ_C (151 MHz, $CDCl_3$)

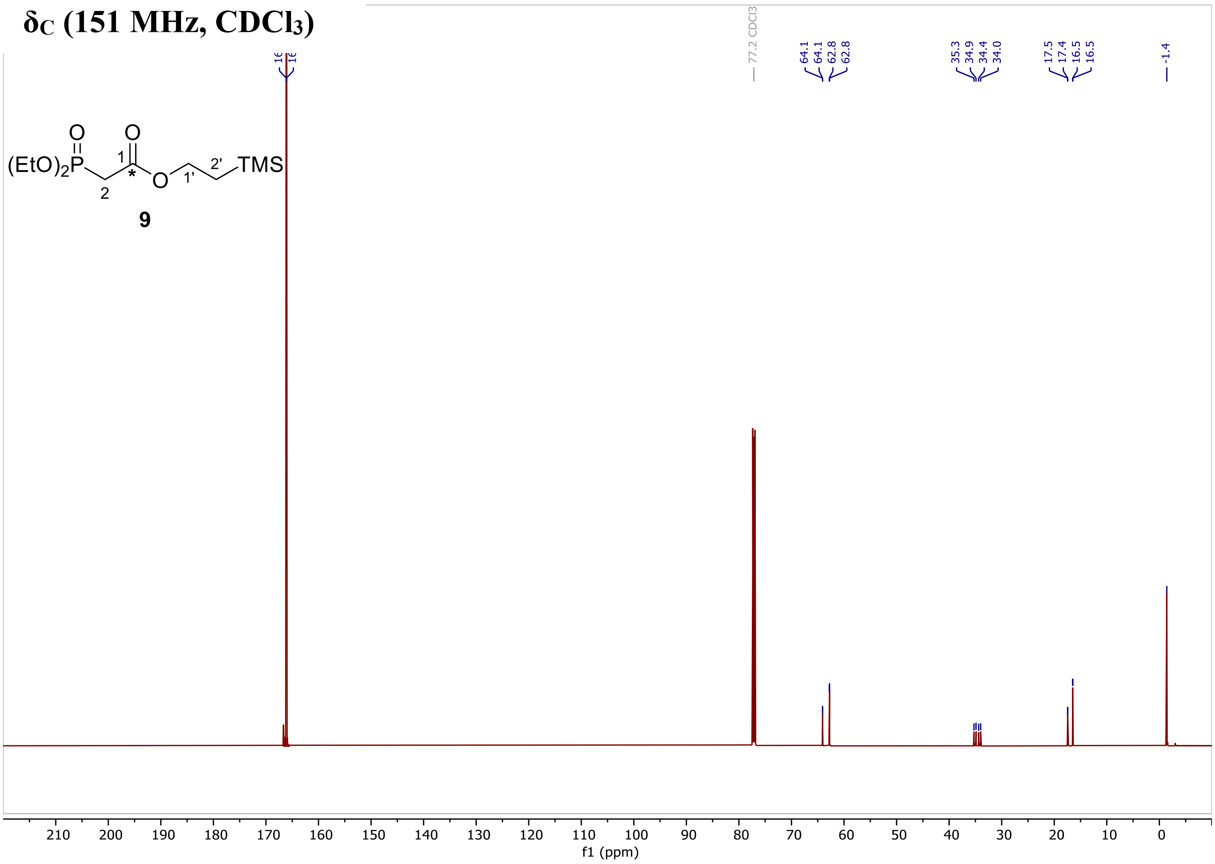

δ_P (162 MHz, $CDCl_3$)

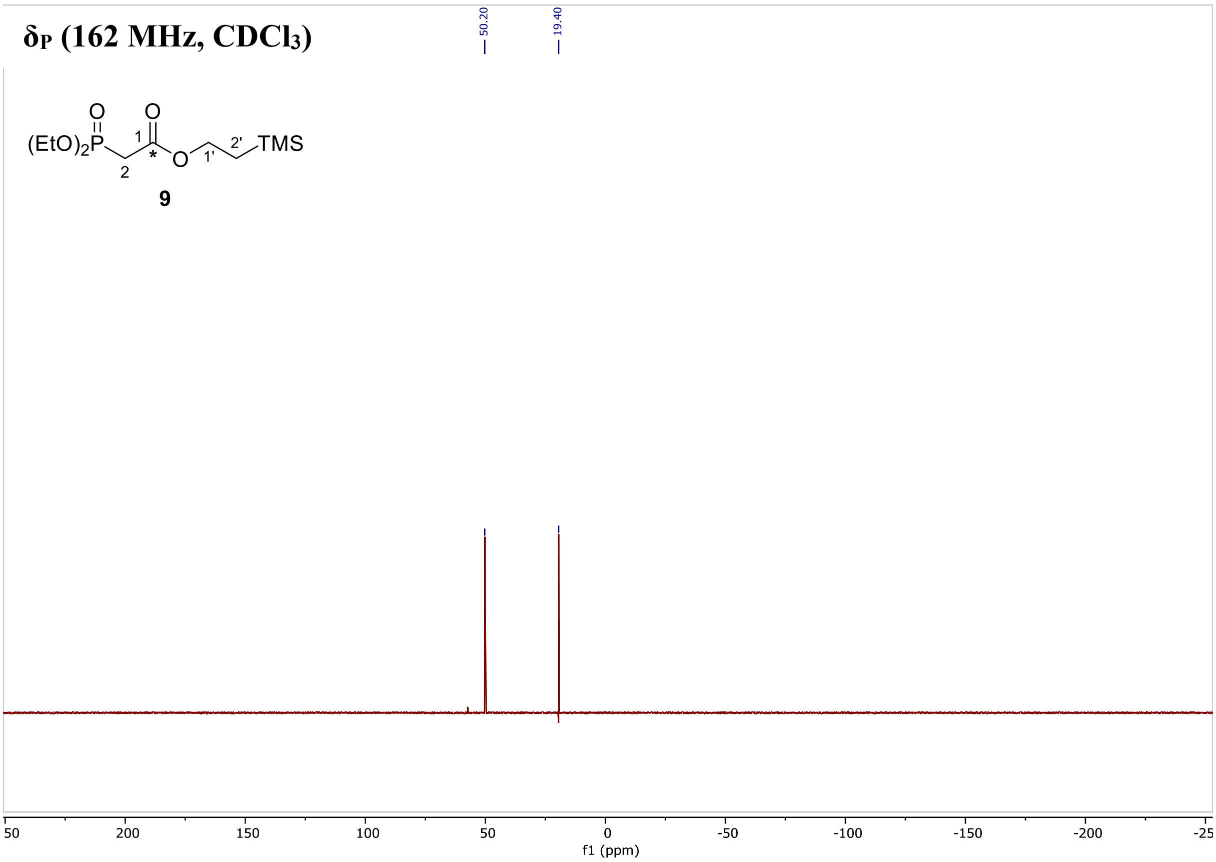

δ$_H$ (600 MHz, CDCl$_3$)

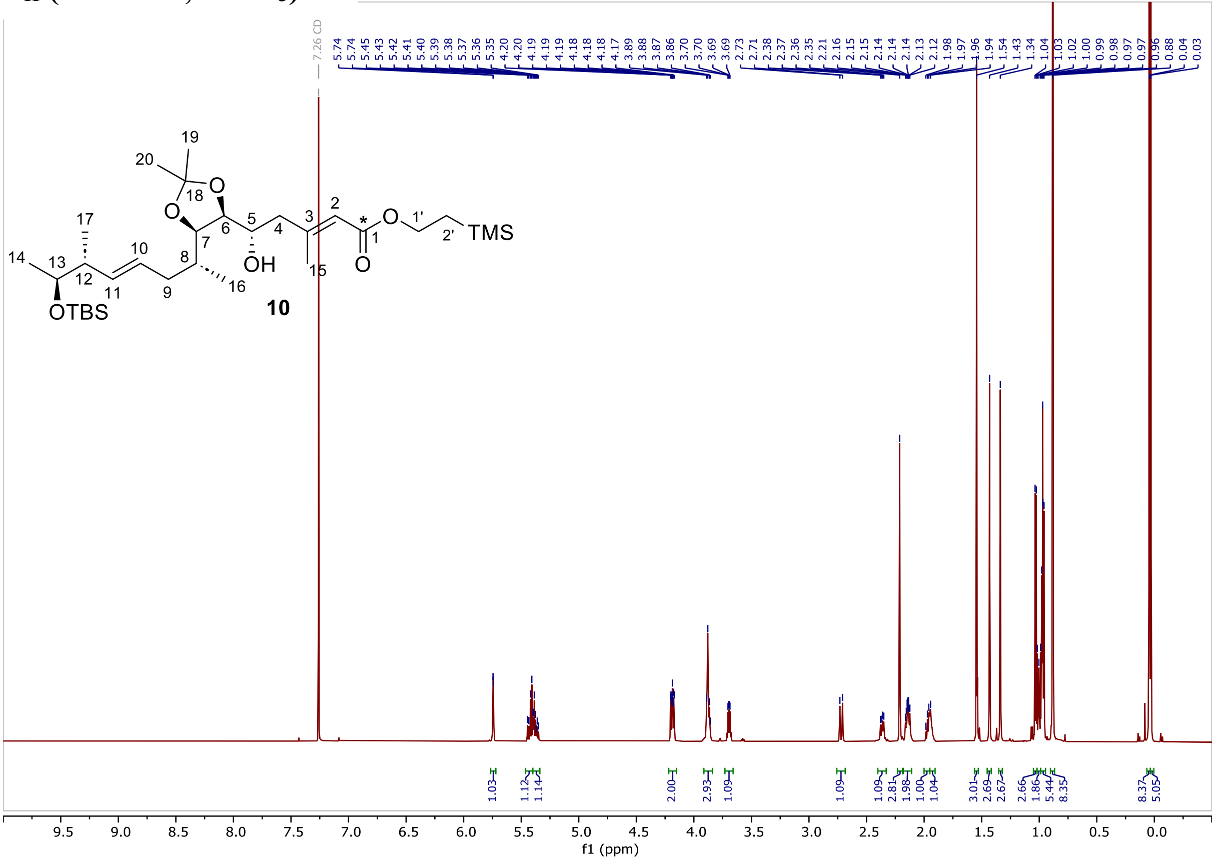

δ$_C$ (151 MHz, CDCl$_3$)

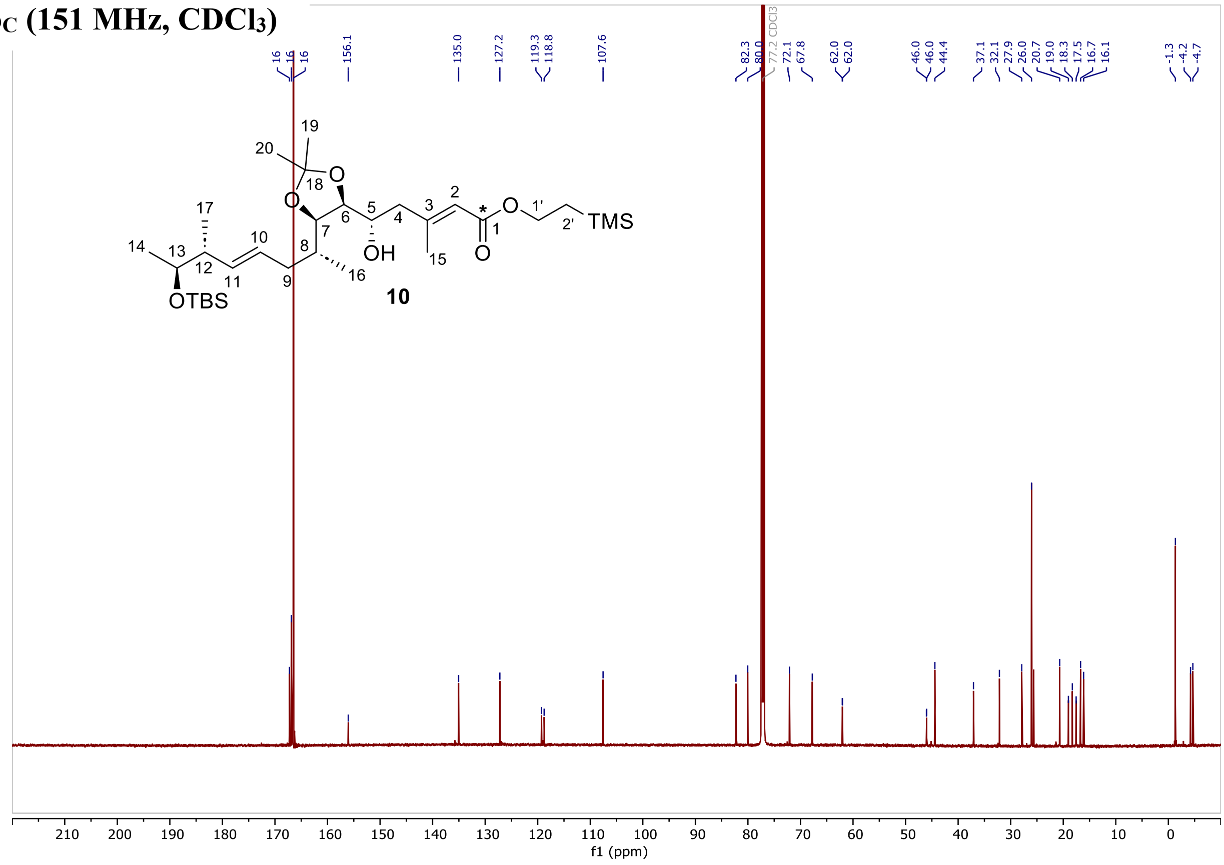

δ$_H$ (400 MHz, $CDCl_3$)

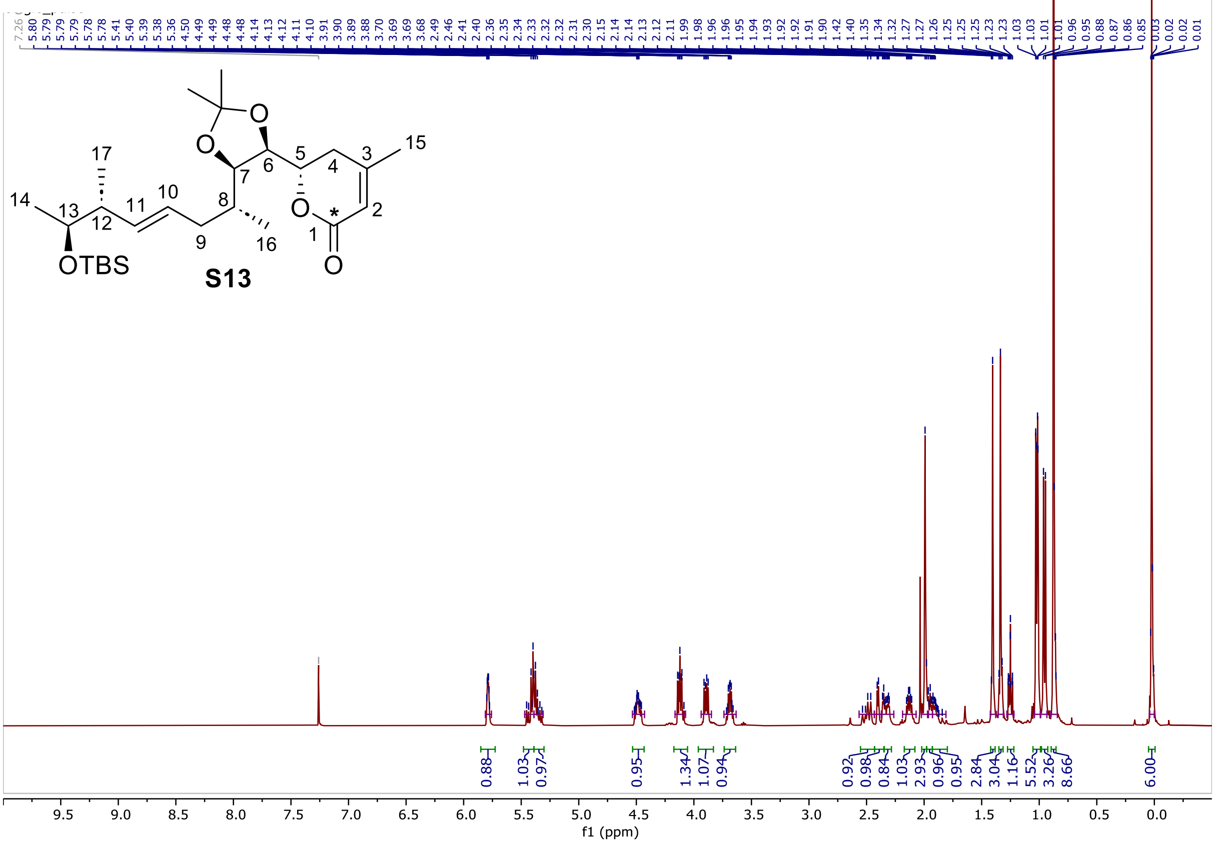

δ$_C$ (101 MHz, $CDCl_3$)

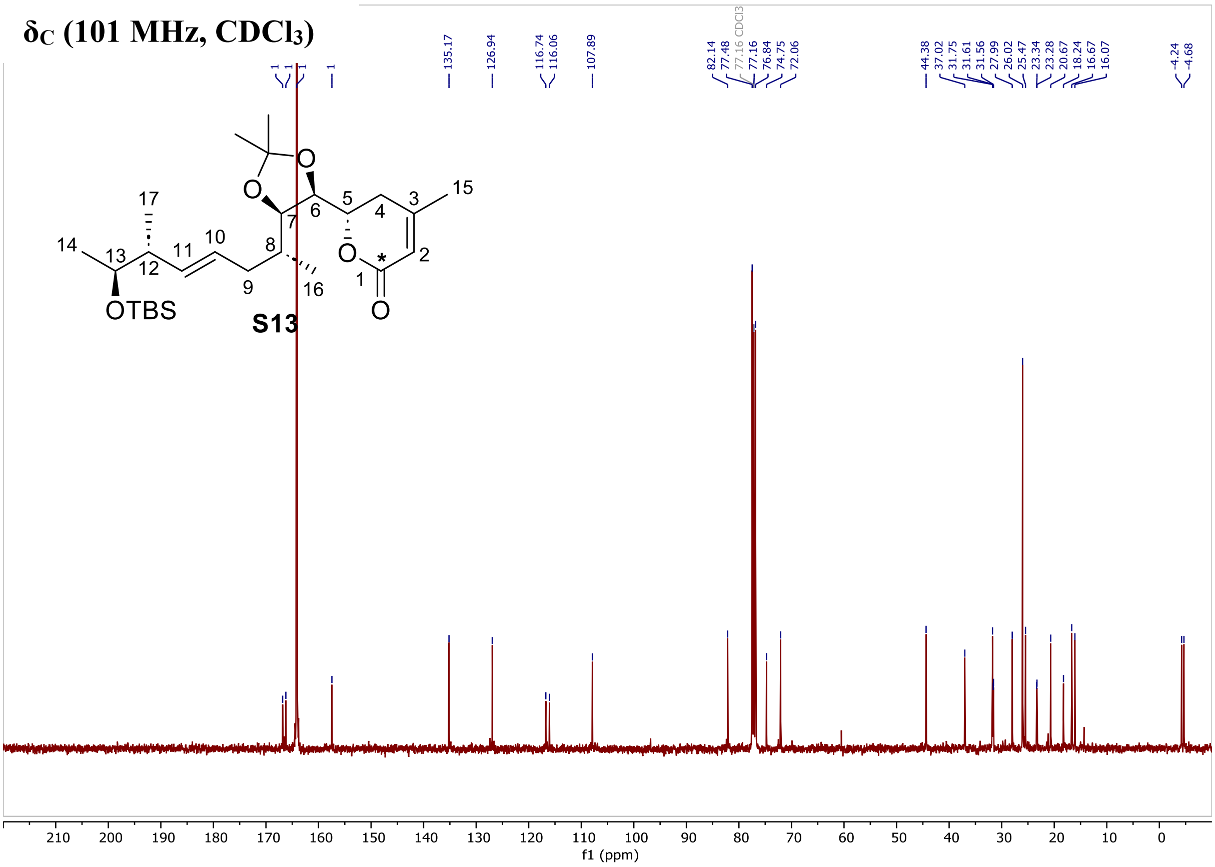

δ$_H$ (600 MHz, CDCl$_3$)

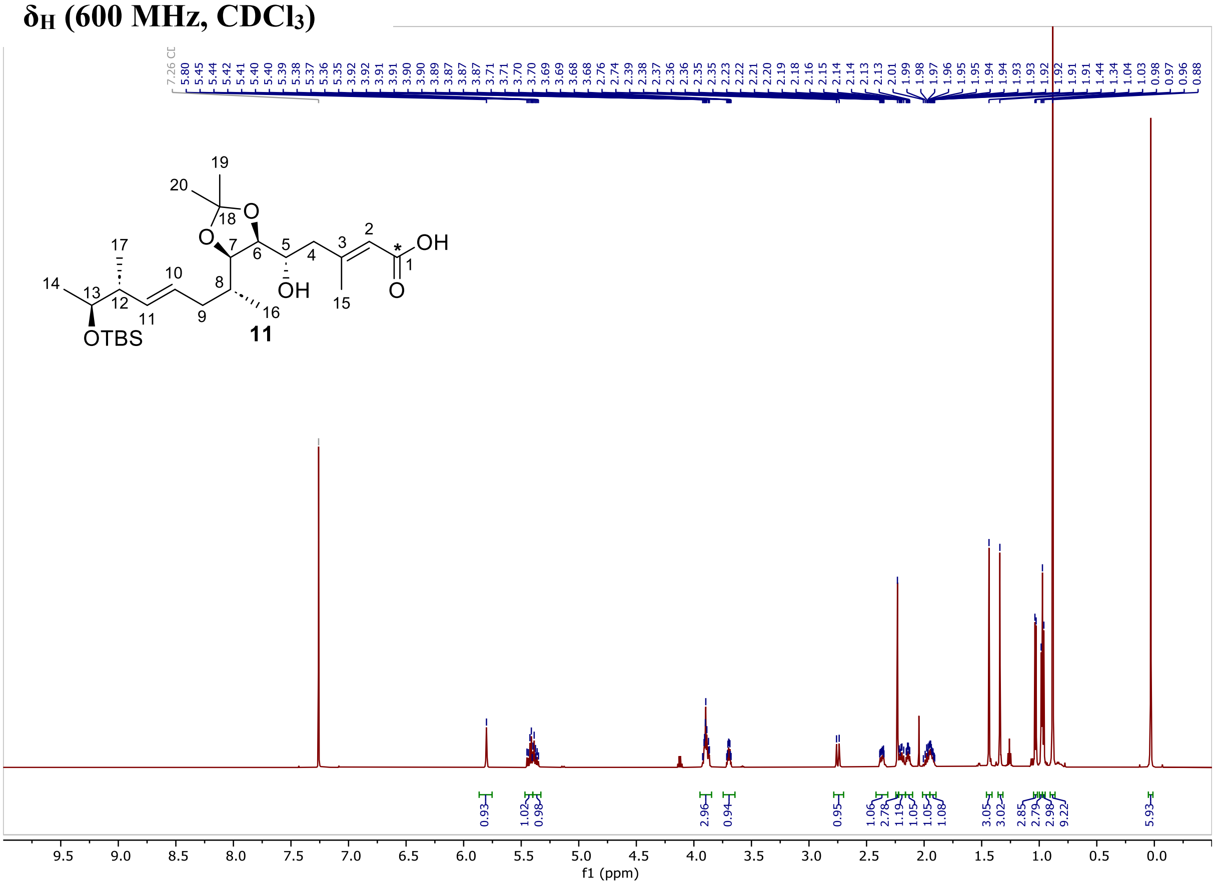

δ$_C$ (151 MHz, CDCl$_3$)

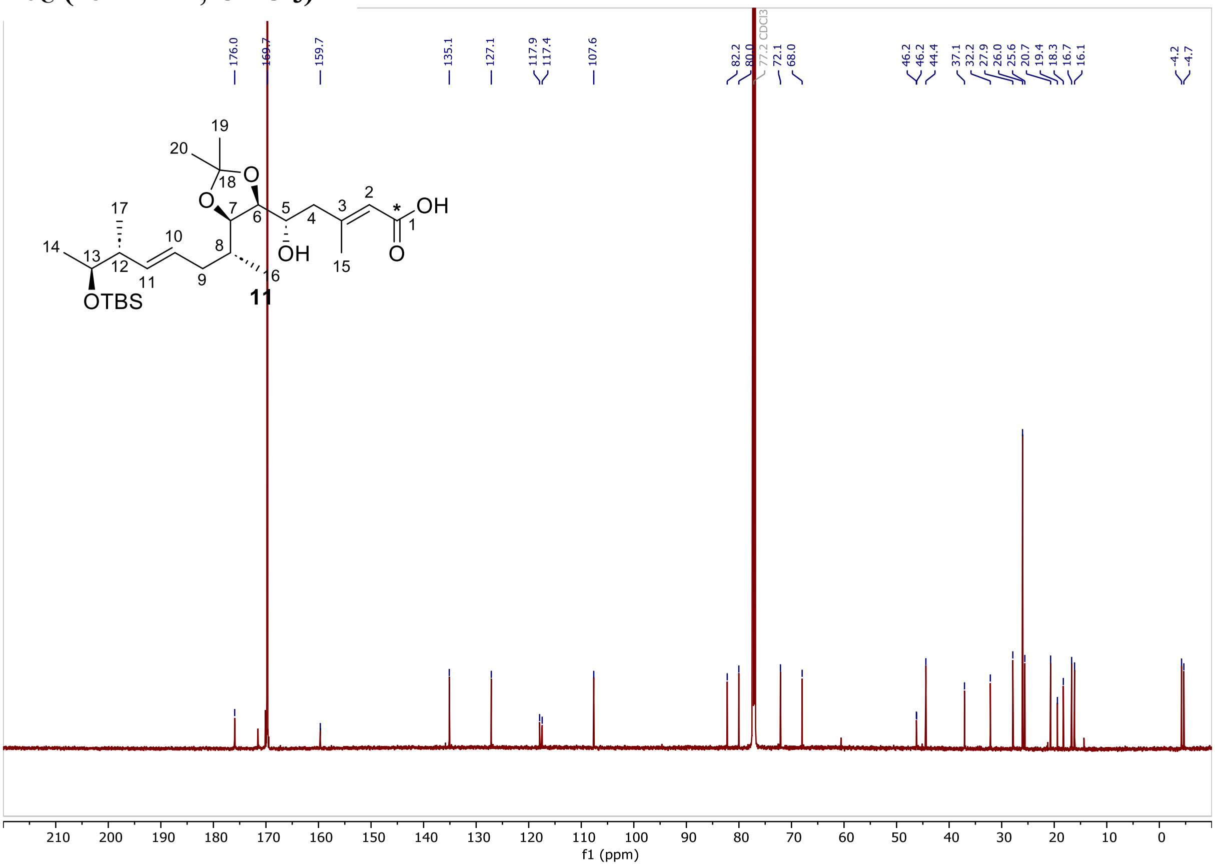

δ_H (600 MHz, $CDCl_3$)

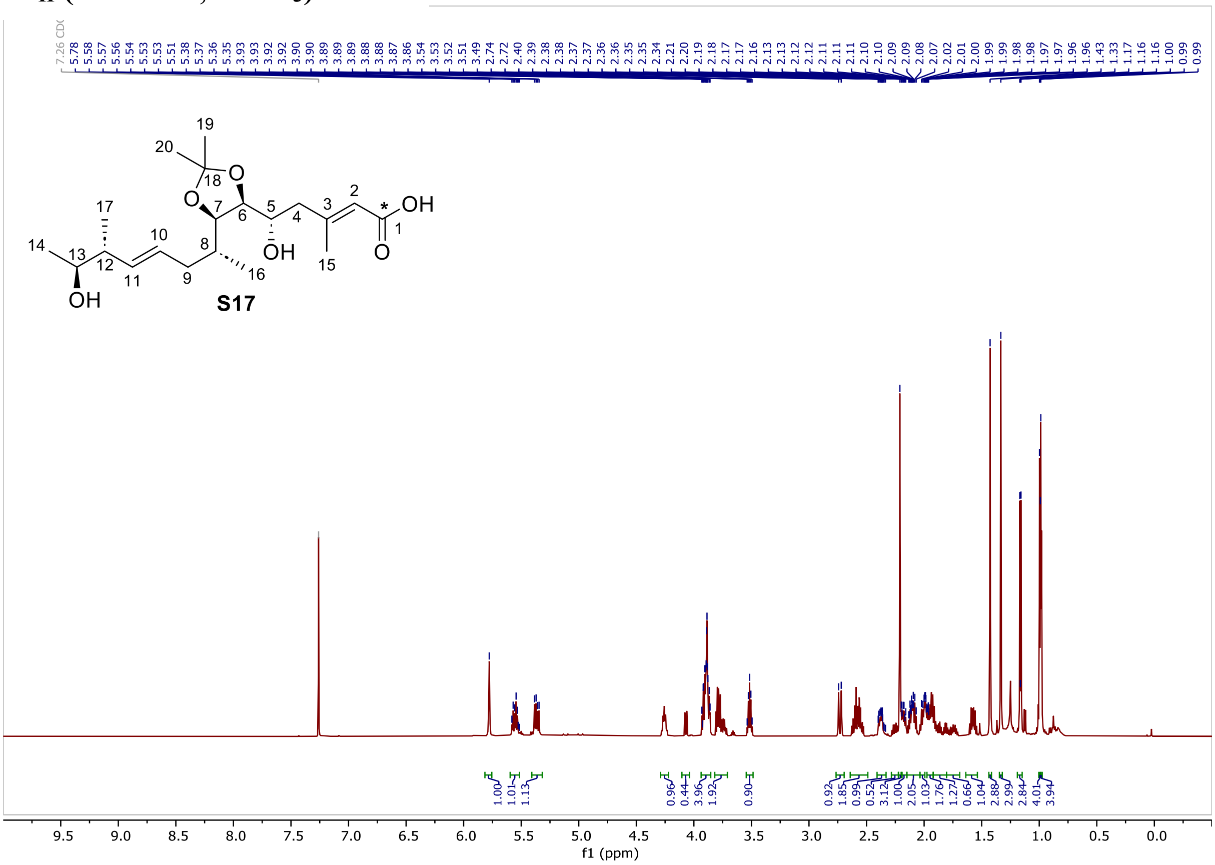

δ_C (151 MHz, $CDCl_3$)

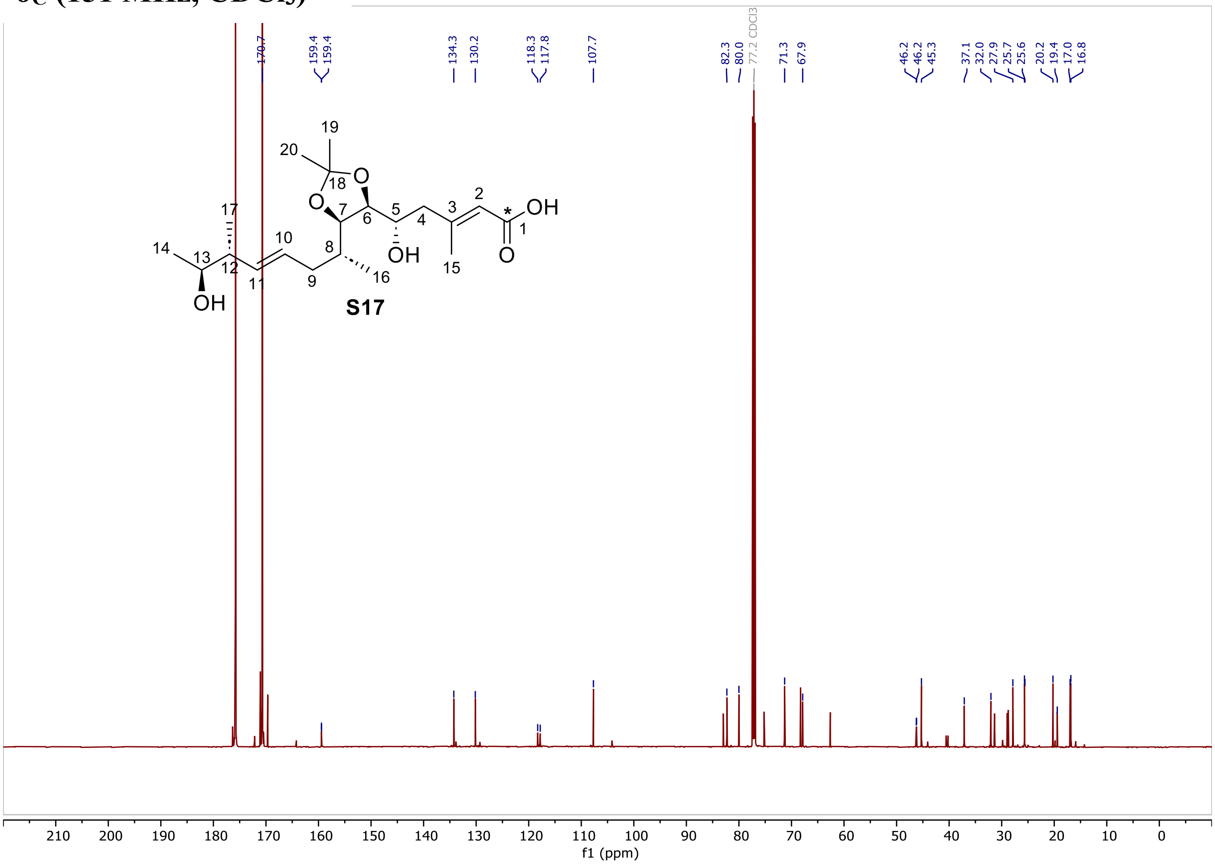

δH (600 MHz, CDCl3)

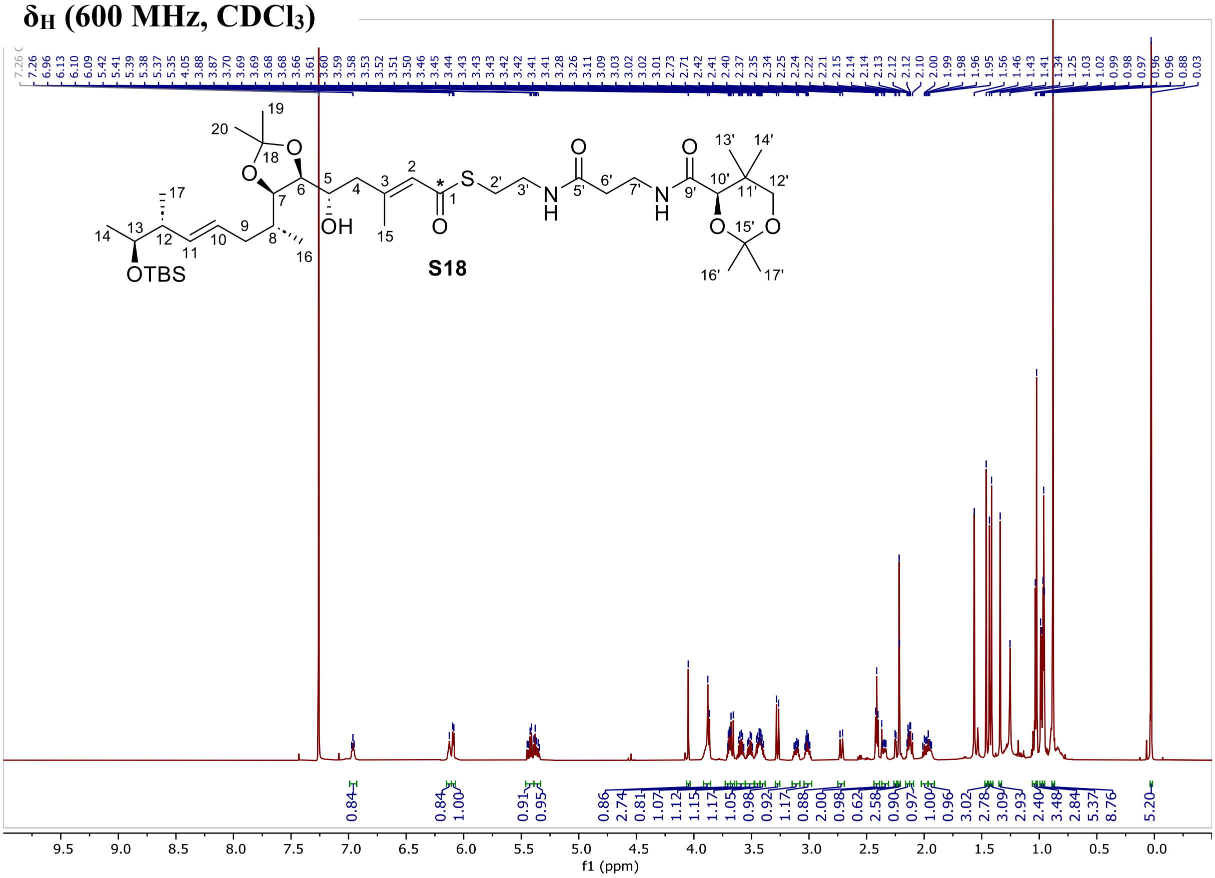

δC (151 MHz, CDCl3)

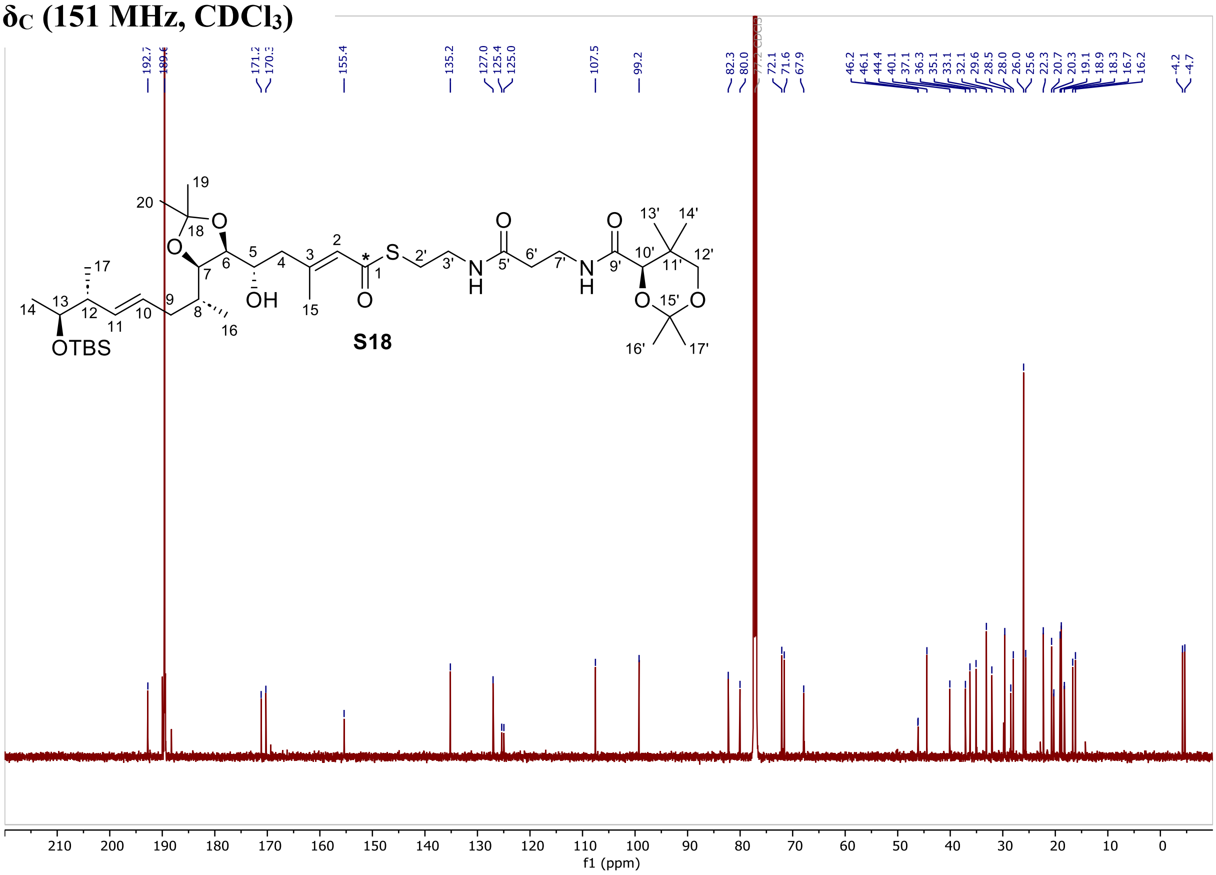

δ$_H$ (600 MHz, CD_3OD)

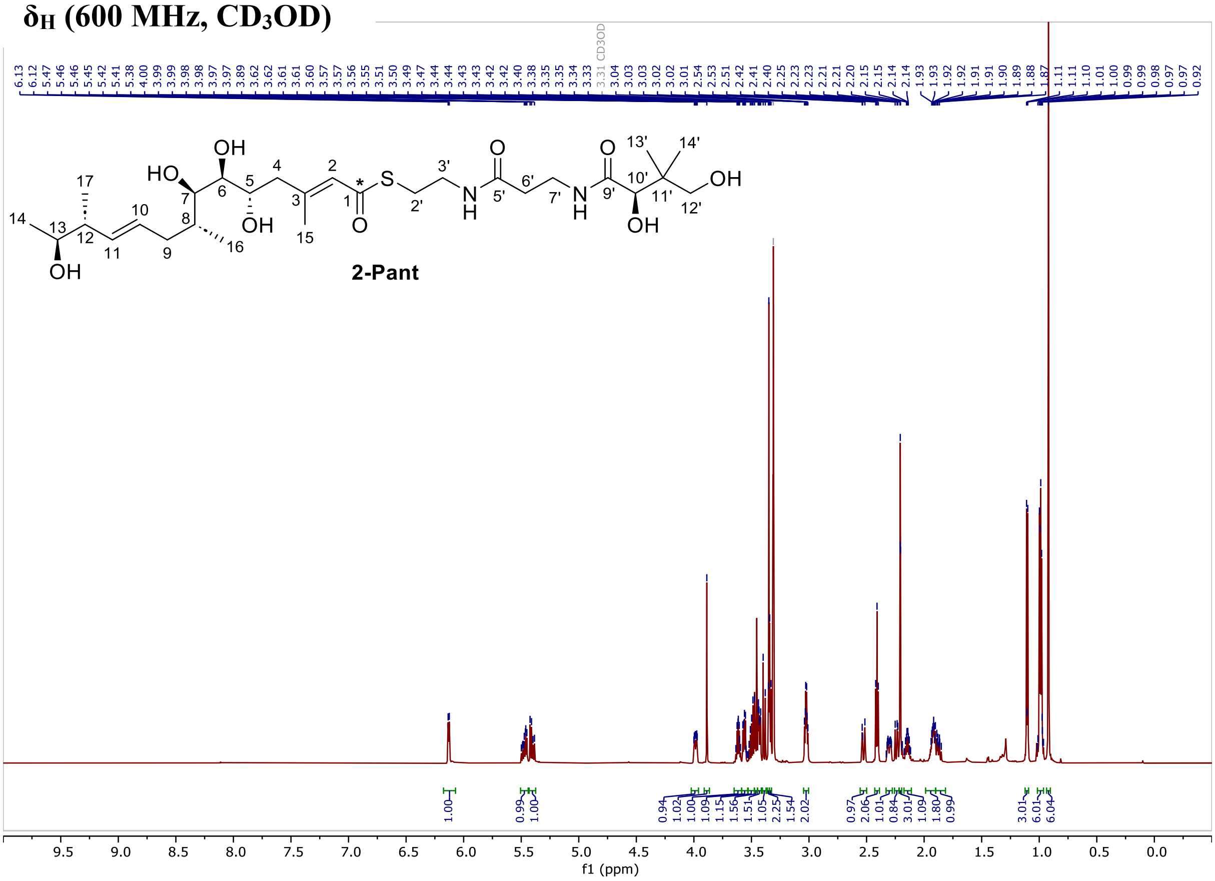

δ$_C$ (151 MHz, CD_3OD)

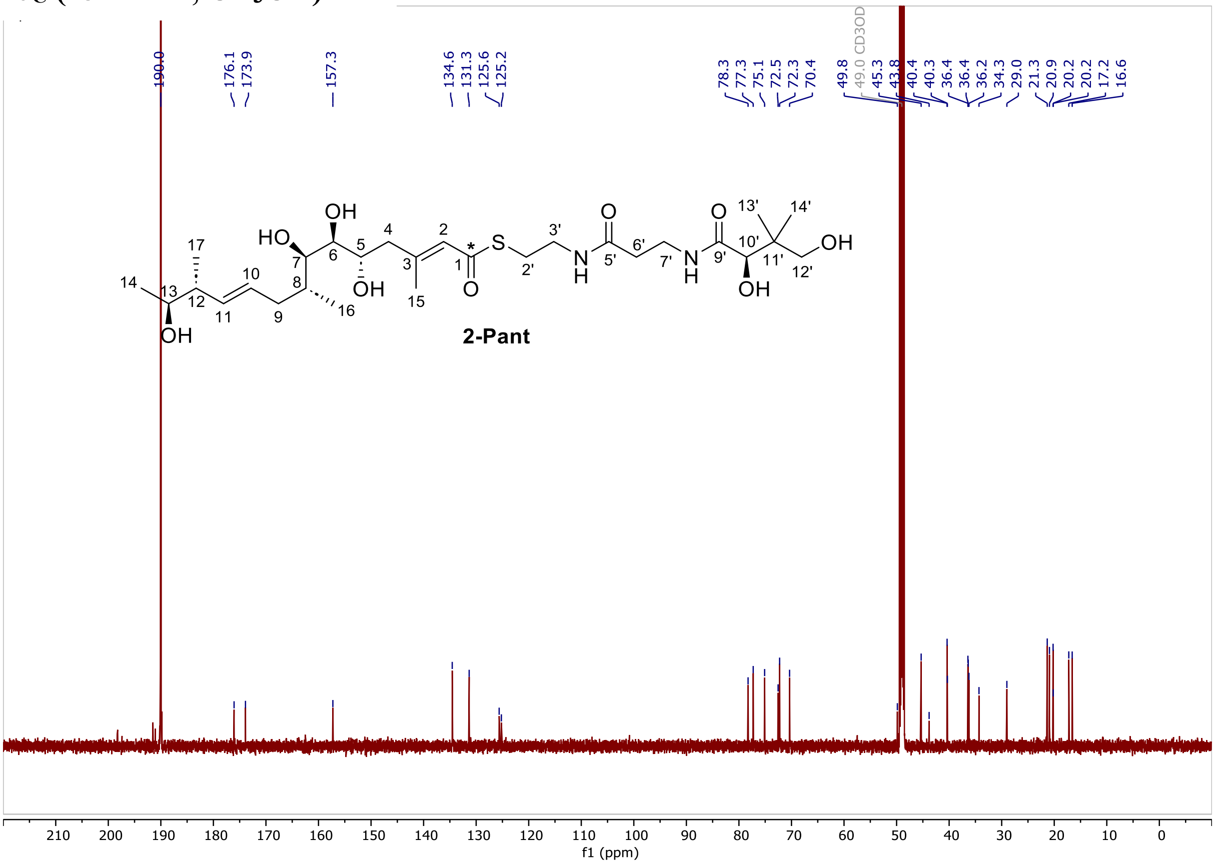

δ$_H$ (700 MHz, CD_3OH)

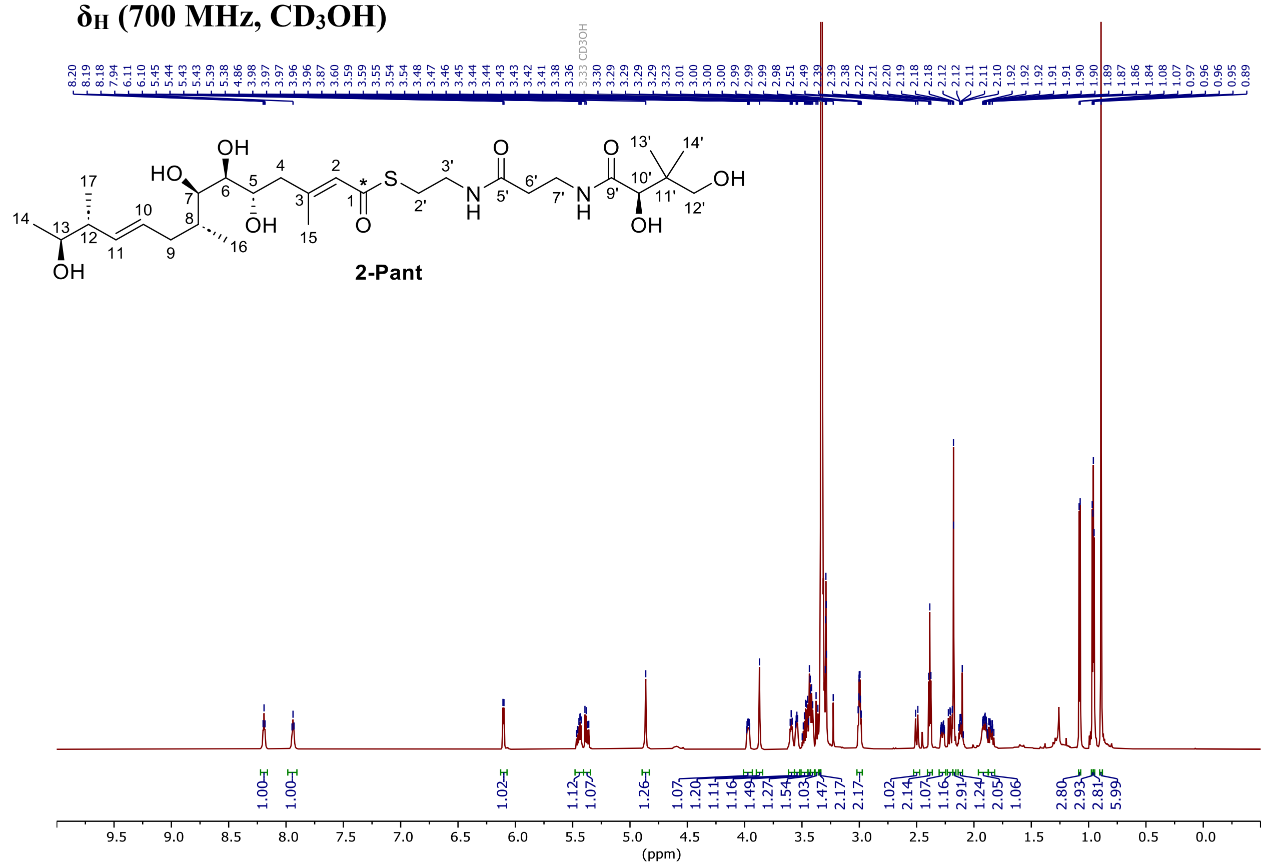

δ$_H$ (700 MHz, CD_3OH)

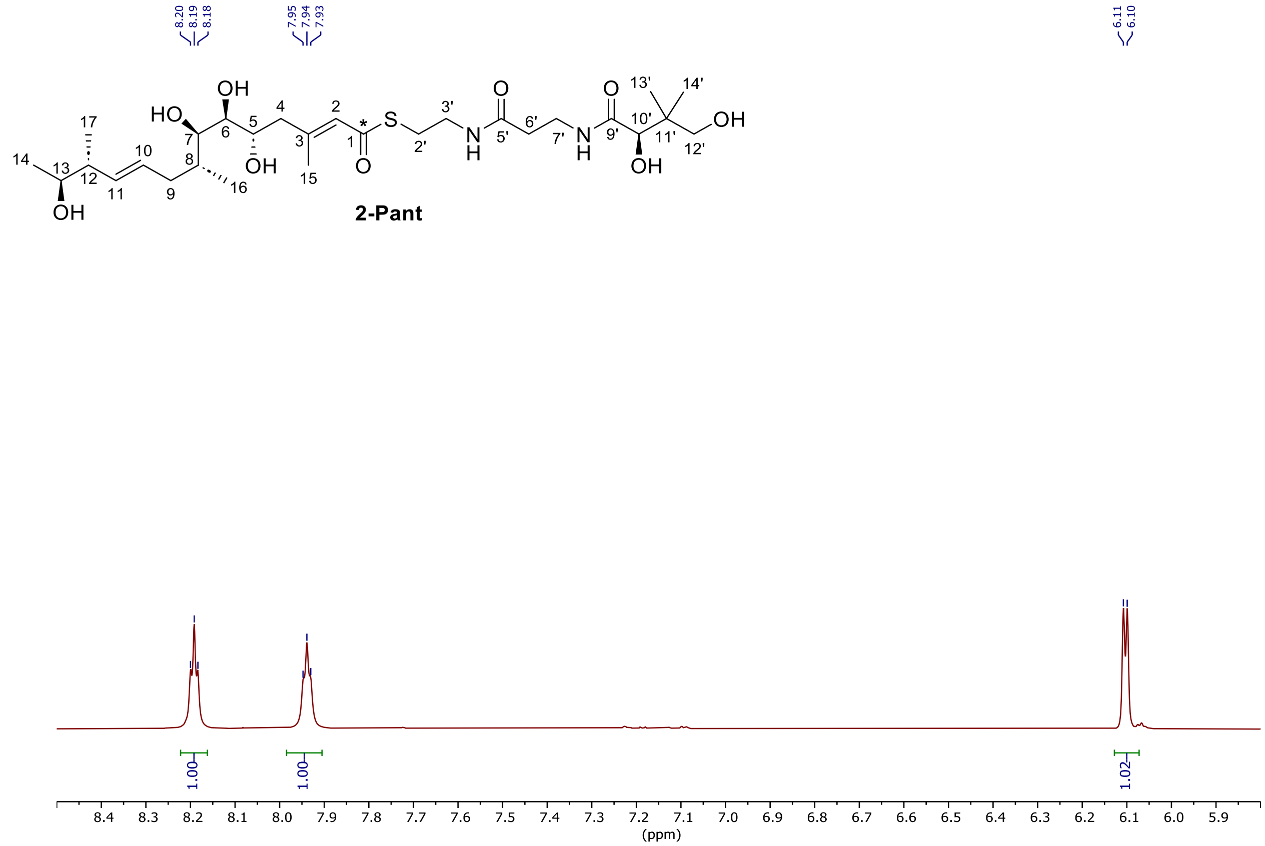

δH (400 MHz, CDCl3)

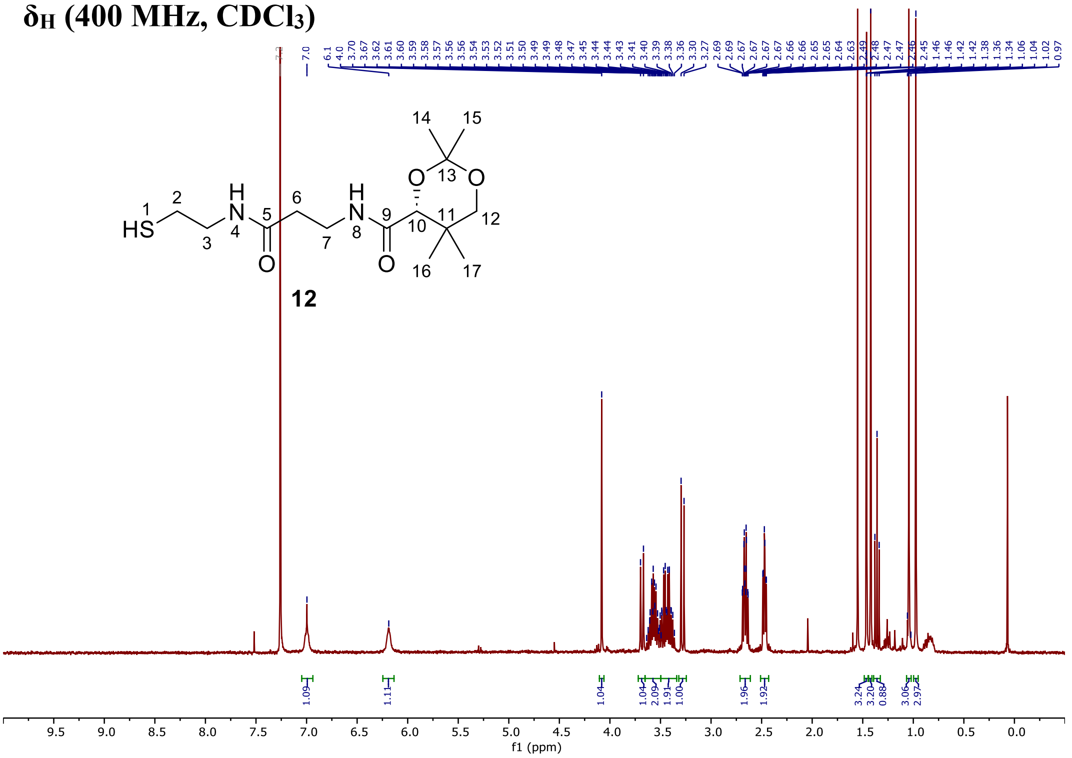

δc (101 MHz, CDCl3)

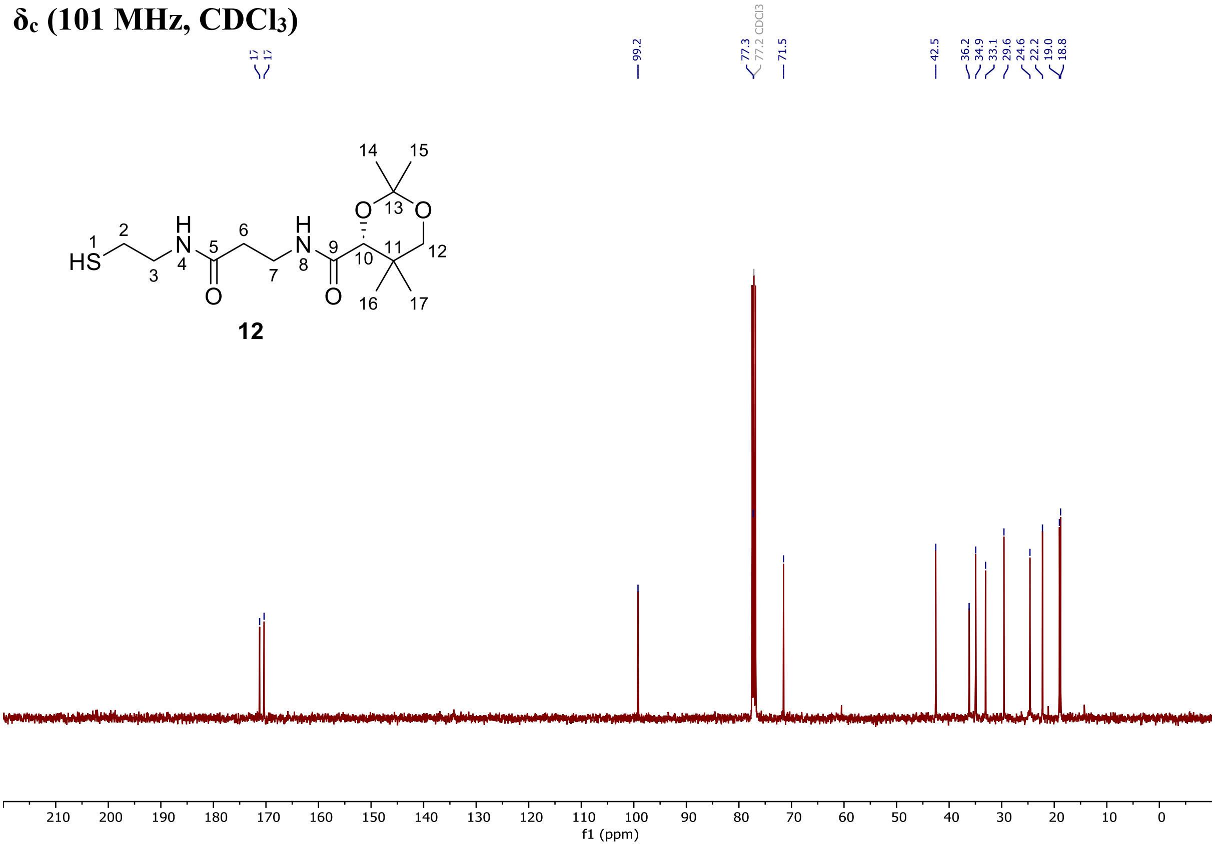

List of Constructs

MmpA3a
MHHHHHHGKPIPNPLLGLDSTENLYFQGIDPFTMAPLAAKAAPVVPVADDECAQFLRQSLAA
MLYCEPGQIRDGSRFLELGLDSVIAAQWIREINKHYQLKIPADGIYTYPVFKAFTQWVGTQL
Q
Calc Mw: 14038.12 Da

MmpAE.6His

MHHHHHHGKPIPNPLLGLDSTENLYFQGIDPFTHSSDDLLDTILNQVQSGALAADSACAVVE
DVLAQRGGGSGGGSMTLKTILDELREGRLSIEQAKARLRMLPAPQP
Calc Mw: 11584.06 Da

MmpAE (His$_6$ cleaved)
GIDPFTHSSDDLLDTILNQVQSGALAADSACAVVEDVLAQRGGGSGGGSMTLKTILDELREG
RLSIEQAKARLRMLPAPQP
Calc Mw: 8431.51 Da (^{15}N: 8537.51 Da)

MmpE_KS
MHHHHHHGKPIPNPLLGLDSTENLYFQGIDPFTGDIAIISMAGRFPGAASVEQFWDNLRAGV
DSITQVPATRWDTDTYFSAQKGEPGRAYCRWGGFIEDVDRFDSLFFGISPREAQLMDPQERL
LLQEVWTLFERIGYTRDYLAESCDGEVGVFTGSMYQQYHAFDADVPRKATVALSSTASMANR
ISHFFNLKGPSLALDTMCSSAATALHLACESLRSGSCRLAVVGATNLSIHPFKYIALCQGQL
LASHPGVRAYGQADGFLPGETVAAVLLKPLKQAIEEGDTVTAVIKSTAISHKGYTPGYSVPS
AAAFETLLSRHFQRSGIDPRSVDYVETSASGATLGDVAEFQALRSVYGPRNTHLPHCRLGSV
KSNIGHAEAAIGLAQVIKVALQLRDRVLVPSILTEPLNPQTRADEAPLRAQRSLEHWHHPSD
APGGMPWPTPRRAAVHTFAAGGANAHLLLQAYDAAPAAVEPVSTPGPWLLVFSAATNERLQA
VLGSARQALAATPSLTLDDLAFTLSQGREAMNCRVAWVQRQPGALLDTLDAALACVHRQQGS
VDGVVIHYGDLEEVEVPDTAPLERQTLSLEEQARAWVVGQISLQQVSRQASLRAGRRIVELP
TYPFAKVHCW
Calc Mw: 68366.36 Da

MmpE_KS_M214A

MHHHHHHGKPIPNPLLGLDSTENLYFQGIDPFTGDIAIISMAGRFPGAASVEQFWDNLRAGV
DSITQVPATRWDTDTYFSAQKGEPGRAYCRWGGFIEDVDRFDSLFFGISPREAQLMDPQERL
LLQEVWTLFERIGYTRDYLAESCDGEVGVFTGSMYQQYHAFDADVPRKATVALSSTASMANR
ISHFFNLKGPSLALDTACSSAATALHLACESLRSGSCRLAVVGATNLSIHPFKYIALCQGQL
LASHPGVRAYGQADGFLPGETVAAVLLKPLKQAIEEGDTVTAVIKSTAISHKGYTPGYSVPS
AAAFETLLSRHFQRSGIDPRSVDYVETSASGATLGDVAEFQALRSVYGPRNTHLPHCRLGSV
KSNIGHAEAAIGLAQVIKVALQLRDRVLVPSILTEPLNPQTRADEAPLRAQRSLEHWHHPSD
APGGMPWPTPRRAAVHTFAAGGANAHLLLQAYDAAPAAVEPVSTPGPWLLVFSAATNERLQA
VLGSARQALAATPSLTLDDLAFTLSQGREAMNCRVAWVQRQPGALLDTLDAALACVHRQQGS
VDGVVIHYGDLEEVEVPDTAPLERQTLSLEEQARAWVVGQISLQQVSRQASLRAGRRIVELP
TYPFAKVHCW
Calc Mw: 68306.25 Da

MmpE_ACP.6His

```
MHHHHHHGKPIPNPLLGLDSTENLYFQGIDPFTLEANAEDVHEVVRAQIAALTQIDPHTLQL
GKPLLDYGLNSVTMLVLAACLCQALEGLDESRDGPQLVLCRTAGALIEQLEQMLAPT
Calc Mw: 13005 Da
```

MmpE_ACP (His_6 cleaved)

```
GIDPFTLEANAEDVHEVVRAQIAALTQIDPHTLQLGKPLLDYGLNSVTMLVLAACLCQALEG
LDESRDGPQLVLCRTAGALIEQLEQMLAPT
```

Calc Mw: 9852.34 Da (^{15}N: 9967.34 Da)

MmpE_OR

```
MGSSHHHHHHSSGLVPRGSHMASMTGGQQMGRGSEFYAFDFAHQPAALPERAWAGKRGVVLG
AGPAGLVAALTLAANGFEQVYVVEKRTAINRMQMVTLYPHTLPYLKSIGVLDAVARRAARIV
RHDFYLNSAGQRTRYFSRELPAGILDSIDASMAYGDETVAELFVGESVMAISLADLQAVLME
QACARGVRFIADHQATVVADDSRPATWQVLLSATAGAPSFPLDADLVVVADGARSAVAQAAG
IECTLLQTPQGSESWYVFHCASEVATSRLSYEFAFDSRGRLDHCAFGLAYPVRKEFGVAFYS
STPTPPALELLLDKADFFAKSWGVEHQGINWLTQRIAVHHSRAERVVDGNLLLVGDAAGTGS
PNAGLGSGLAISAYGWALNEYCRRAATDREAAADFYQAAASRYPLAWQSRSLHIWDEINNLA
AGVAGQRQTGT
Calc Mw (-M): 47705.71 Da
```


[1] A. J. Winter, R. N. Khanizeman, A. M. C. Barker-Mountford, A. J. Devine, L. Y. Wang, Z. S. Song, J. A. Davies, P. R. Race, C. Williams, T. J. Simpson, C. L. Willis, M. P. Crump, *Angew. Chem. Int. Ed.* **2023**, *62*, e202312514.

[2] L. Nagarapu, S. Karnakanti, R. Bantu, *Tetrahedron* **2012**, *68*, 5829-5832.

[3] a C. G. Zhao, Z. Y. Yuan, Y. Y. Zhang, B. Ma, H. L. Li, S. C. Tang, X. G. Xie, X. G. She, *Org. Chem. Front.* **2014**, *1*, 105-108; bT. Hudlicky, H. Luna, J. D. Price, F. Rulin, *J. Org. Chem.* **1990**, *55*, 4683-4687; c S. Sengupta, T Sim, *Eur. Jour. Org. Chem.* **2014**, *2014*, 5063-5070.

[4] aV. Agarwal, S. Diethelm, L. Ray, N. Garg, T. Awakawa, P. C. Dorrestein, B. S. Moore, *Org. Lett.* **2015**, *17*, 4452-4455; b A. S. Haines, X. Dong, Z. Song, R. Farmer, C. Williams, J. Hothersall, E. Ploskon, P. Wattana-amorn, E. R. Stephens, E. Yamada, R. Gurney, Y. Takebayashi, J. Masschelein, R. J. Cox, R. Lavigne, C. L. Willis, T. J. Simpson, J. Crosby, P. J. Winn, C. M. Thomas, M. P. Crump, *Nat. Chem. Biol.* **2013**, *9*, 685-692.

[5] S. Tayyab, S. Qamar, M. Islam, *Biochem. Ed.* **1991**, *19*, 149-152.

[6] W. F. Vranken, W. Boucher, T. J. Stevens, R. H. Fogh, A. Pajon, P. Llinas, E. L. Ulrich, J. L. Markley, J. Ionides, E. D. Laue, *Proteins: Struct. Funct. Bioinf.* **2005**, *59*, 687-696.

[7] J. Jumper, D. Hassabis, *Nat. Methods* **2022**, *19*, 11-12.

[8] a B. Mészáros, G. Erdos, Z. Dosztányi, *Nucl. Ac. Res.* **2018**, *46*, W329-W337; b L. J. McGuffin, K. Bryson, D. T. Jones, *Bioinformatics* **2000**, *16*, 404-405.

[9] M. H. M. Eppink, H. A. Schreuder, W. J. H. VanBerkel, *Protein Sci.* **1997**, *6*, 2454-2458.
